# Interferon regulatory factor 5 in myeloid cells promotes obstructive nephropathy-induced renal fibrosis in vivo

**DOI:** 10.1172/jci.insight.201877

**Published:** 2026-08-06

**Authors:** Jia Wei, Gengyu Du, Zixia Li, Min Yang, Ting Chen, Zihao Xu, Zhen Yuan, Yidan Zheng, Xiang Yan

**Affiliations:** 1 Department of Urology and; 2 Department of Pathology, Children’s Hospital, Zhejiang University School of Medicine, National Clinical Research Center for Child Health, Hangzhou, China.; 3 Department of Cardiology, The First Affiliated Hospital, Zhejiang University School of Medicine, Hangzhou, China.; 4 Department of cardiovascular surgery, Union Hospital, Tongji Medical College, Huazhong University of Science and Technology, Wuhan, China.

**Keywords:** Immunology, Nephrology, Fibrosis

## Abstract

Obstructive nephropathy is a significant and preventable contributor to chronic kidney disease, yet no disease-modifying antifibrotic agents are currently available. We hypothesized that interferon regulatory factor 5 (IRF5) functions as a macrophage transcriptional regulator that directly transactivates matrix metalloproteinase 9 (MMP9) to initiate early extracellular matrix (ECM) remodeling. Analysis of 30 human obstructive nephropathy biopsy specimens demonstrated that IRF5^+^CD68^+^ macrophage density increased progressively with fibrosis severity and correlated significantly with α-smooth muscle actin–positive (α-SMA^+^) areas. In the murine unilateral ureteral obstruction (UUO) model, both global and myeloid-specific *Irf5* deletion significantly attenuated collagen deposition, immune cell infiltration, and fibrotic gene expression compared with WT controls. Cleavage under targets and tagmentation (CUT&Tag) analysis demonstrated that IRF5 directly binds the *Mmp9* enhancer region and increases chromatin accessibility. Consequently, myeloid-specific *Irf5* KO significantly reduced *Mmp9* mRNA and MMP9 protein levels. Pharmacological inhibition using the IRF5 inhibitor N5-1 mitigated established fibrosis, downregulated α-SMA and MMP9 expression, and reduced CD68^+^ macrophage infiltration. These findings identify the IRF5/MMP9 axis as a therapeutically targetable pathway driving macrophage-mediated ECM expansion and provide preclinical evidence supporting IRF5 inhibition as a potential treatment strategy for patients with obstructive nephropathy.

## Abstract 2

Macrophage IRF5 drives kidney fibrosis after obstruction, validated in vivo

## Graphical abstract

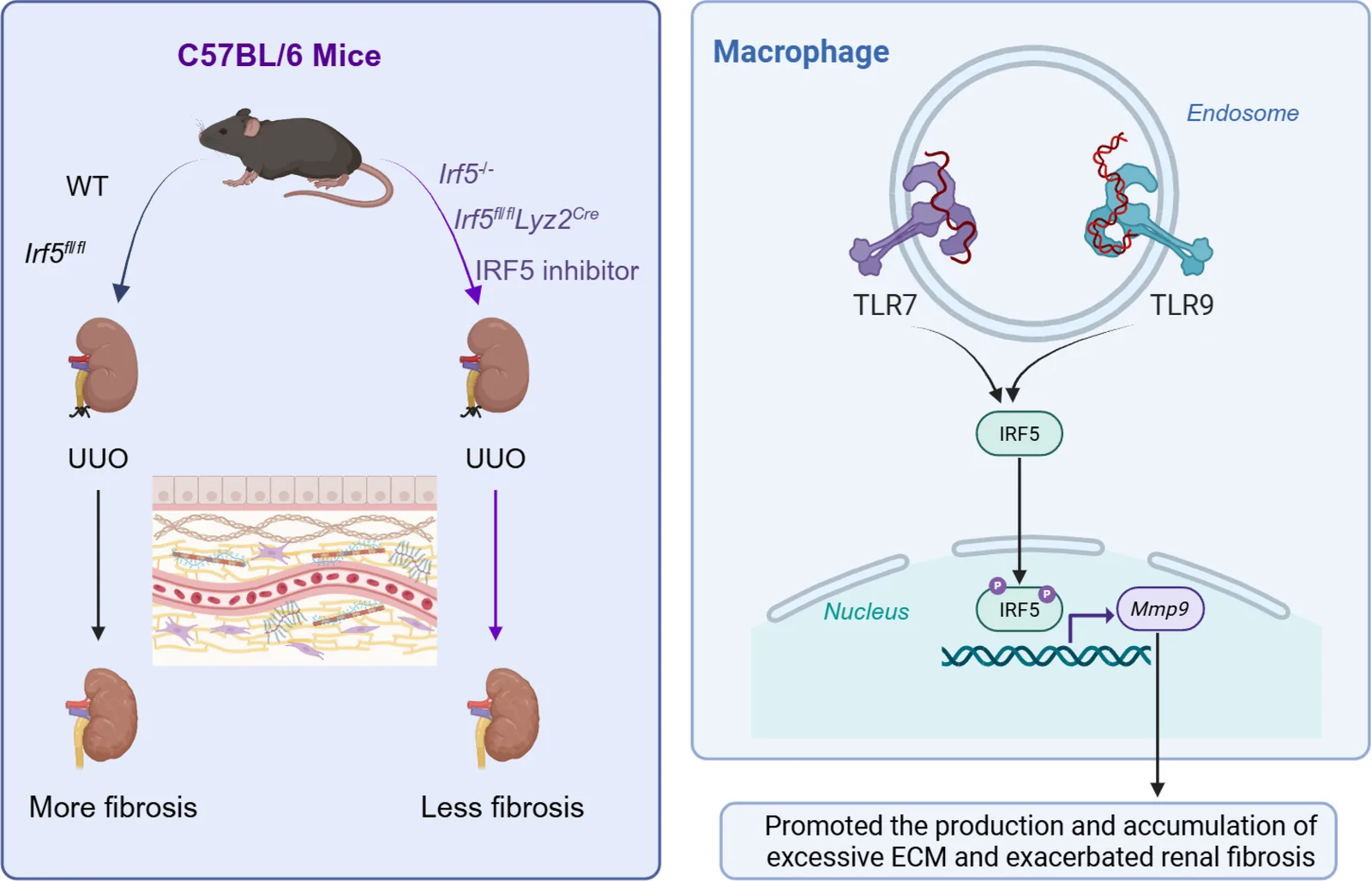

## Introduction

Chronic kidney disease (CKD), a global disorder affecting about 10% of the population, may progress to end-stage renal disease (ESRD). Obstructive nephropathy is one of the main causes of CKD, often caused by various urinary tract problems such as congenital anatomical abnormalities, kidney stones, prostatic hyperplasia, and urinary tract tumors. These problems lead to the obstruction of urine excretion, causing hydronephrosis, further damaging renal tubules, resulting in interstitial inflammation and fibrosis, and impairing renal function (1).

The main pathological characteristics of obstructive nephropathy are renal interstitial inflammation and fibrosis. The abnormally accumulated extracellular matrix (ECM) destroys the normal structure of the kidney, leading to scar formation and ultimately contributing to the continuous decline of renal function (2–5). Notably, monocytes/macrophages are crucial in renal interstitial fibrosis (6–8). They quickly migrate after obstruction, release cytokines like TGF-β1, TNF-α, and ILs, which worsen tubular cell injury and promote fibroblast-myofibroblast conversion, though their activation and regulatory details need further study.

Interferon regulatory factor 5 (IRF5) is a significant transcription factor, primarily activated through the TLR signaling pathway (9). IRF5 is predominantly expressed in myeloid cells, especially macrophages, and is regarded as a key regulator of macrophage activation. Its classical downstream pathways involve promoting the transcription of type I IFN genes and the activation of the inflammatory response (10). As an important immunomodulatory entity, IRF5 has demonstrated its crucial role in various inflammation associated and fibrosis related diseases. In the context of liver fibrosis, IRF5 drives hepatocyte death and the fibrotic process by modulating the expression of proinflammatory factors in macrophages. The upregulation of IRF5 is closely correlated with macrophage activation within the fibrotic liver (11). Similarly, in systemic sclerosis, the activation of IRF5 is linked to the upregulation of proinflammatory and fibrotic factors (12). These findings suggest the potential involvement of IRF5 in the fibrotic process. However, the mechanism of IRF5 in obstructive renal interstitial fibrosis remain unexplored.

In this study, we analyzed patients with obstructive nephropathy, global and myeloid-specific *Irf5*-KO mice with unilateral ureter obstruction (UUO) and utilized a pharmacologic IRF5 inhibitor to address the role of IRF5 in renal fibrosis. We hypothesize that IRF5 in macrophages promotes renal fibrosis in obstructive nephropathy and that IRF5 inhibition could mitigate the progress of renal fibrosis.

## Results

### The expression of IRF5 is significantly increased in the renal tissues of patients with obstructive nephropathy.

To elucidate the role of IRF5 in renal fibrosis associated with obstructive nephropathy, a total of 42 renal tissue samples were enrolled in this study (Supplemental Table 1 and Supplemental Table 2; supplemental material available online with this article; https://doi.org/10.1172/jci.insight.201877DS1). IHC was employed to evaluate the expression of fibrosis markers α-SMA and Vimentin (VIM), along with IRF5, in kidney tissues with varying degrees of fibrosis. Positive areas of α-SMA and VIM increased along with the extent of fibrosis (Figure 1, A and B). The number of IRF5^+^ cells was also significantly increased in the fibrotic tissues, and its expression is positively correlated with the degree of fibrosis (Figure 1, A and B). The results of immunofluorescence costaining indicated that the expression of IRF5 and α-SMA was increased. Spearman correlation analysis further revealed a significant positive correlation between the number of IRF5^+^ cells and the percentage area of α-SMA (*r_s_* = 0.7941, *P* < 0.001) (Figure 1, C and D).

### IRF5 was significantly elevated in macrophages in kidneys from patients with obstructive nephropathy.

Upon H&E staining, we detected large areas of densely packed, interstitial inflammatory cells in fibrotic areas. Higher infiltration of CD68^+^ macrophages, CD20^+^ B lymphocytes, and CD3^+^ T lymphocytes were also confirmed in the fibrotic tissues via IHC analysis (Figure 2, A and B). To clarify IRF5 localization within inflammatory cells, immunofluorescence was performed using IRF5, CD68 (macrophage marker), CD20 (B lymphocyte marker), and CD3 (T lymphocyte marker). The results showed that IRF5 was predominantly colocalized in CD68^+^ macrophages. In moderately to severely fibrotic tissues, a significantly higher percentage of IRF5^+^CD68^+^ cells were observed compared with IRF5^+^CD20^+^ and IRF5^+^CD3^+^ cells (Figure 2, C and D). Furthermore, we found that the ratio of IRF5^+^CD68^+^ cells to CD68^+^ cells was significantly elevated in fibrotic kidney tissue, and there was a strong positive correlation between the number of IRF5^+^CD68^+^ cells and α-SMA^+^ areas (*r_s_* = 0.8921, *P* < 0.001) (Figure 3, A and B).

### Macrophage IRF5 expression was significantly increased in the renal tissues of UUO mice.

Unilateral ureteral obstruction (UUO) is a classic animal model of obstructive nephropathy (13). After 7 days of UUO in WT mice, the renal parenchyma in the UUO group was significantly thinner than that in the sham group (Supplemental Figure 1, A and B). H&E staining revealed tubular atrophy, epithelial cell apoptosis, and inflammatory cell infiltration following UUO. MTS showed the progression of renal fibrosis after UUO, with collagen primarily deposited around the glomeruli and tubule, localized in the corticomedullary junction (Supplemental Figure 1, C and D). IHC staining demonstrated a significant increase in α-SMA, VIM, and IRF5 expression in the obstructed kidneys (Supplemental Figure 1, E and F). Consistently, *Acta2*, *Vim*, and *Irf5* mRNA levels were also significantly increased in renal tissues of UUO group as assessed by qPCR assay (Supplemental Figure 1G).

There was a significant increase in inflammatory cells, primarily CD68^+^ macrophages, in the obstructed kidneys after the UUO procedure. Moreover, IRF5 staining was localized to the same regions as CD68 staining, indicating localization of IRF5 expression to CD68^+^ macrophages. There was an increase in IRF5^+^CD68^+^ macrophages in the obstructed kidney after UUO. As expected, there was also a strong positive correlation between the number of IRF5^+^CD68^+^ cells and α-SMA^+^ areas in the obstructed kidneys (*r_s_* = 0.7623, *P* < 0.001) (Figure 4, A and B).

We conducted RNA-seq on renal tissues from WT UUO and WT sham mice. Compared with the sham group, 2,726 genes were > 2-fold upregulated and 1,827 genes were > 2-fold downregulated in UUO kidneys (Figure 4C) (Supplemental Table 3). Gene ontology (GO) enrichment analysis of deferentially expressed genes showed highly enriched categories related to the ECM, external encapsulating structure, positive regulation of cytokine production, and regulation of lymphocyte activation (Figure 4D) (Supplemental Table 4).

### Global deletion of Irf5 prevented UUO-induced renal fibrosis in vivo.

To investigate our hypothesis, UUO was performed in WT mice and *Irf5-KO* (*Irf5^–/–^*) mice. Seven days after surgery, kidney tissue was collected for analysis. IRF5-KO efficiency in WT and KO mice was confirmed by Western blot analysis (Supplemental Figure 2A). MTS showed that total collagen deposition was significantly reduced in the obstructed kidneys of *Irf5^–/–^* mice compared with WT mice. An IHC assessment of α-SMA and VIM demonstrated a significant reduction in the α-SMA^+^ and VIM^+^ areas in the obstructed kidneys of *Irf5^–/–^* mice (Figure 5, A and B). Furthermore, the number of CD68^+^ cells was also significantly decreased (Figure 5, A and B). We then performed qPCR analysis of fibrosis-related indicators (*Acta2* and *Vim*) and proinflammatory markers (*Tnf-α*, *IL-6*, and *IL-1β*) on the obstructed kidneys. Our results demonstrated that *Acta2*, *Vim*, *Tnf-α*, *IL-6*, and *IL-1β* levels significantly decreased in *Irf5^–/–^* mice (Figure 5, C and E). Expression of VIM and α-SMA protein further confirmed that renal fibrosis was more severe in the obstructed kidneys of WT mice (Figure 5, D and F). RNA-seq analysis also revealed that the differentially expressed genes were highly enriched in categories related to the ECM (Figure 6, A and B, and Supplemental Tables 5 and 6).

### Myeloid-specific Irf5 deficiency prevented UUO-induced renal fibrosis in vivo.

Our data indicate that IRF5 was mainly induced in macrophages in kidneys of UUO group, and global *Irf5* deletion prevents renal fibrosis induced by UUO, we further investigated whether *Irf5* deletion in myeloid cells is able to suppress UUO-induced renal fibrosis as well. Western blot analysis demonstrated that IRF5 expression was significantly reduced in *Irf5^fl/fl^Lyz2^Cre^* UUO mice (Supplemental Figure 2B). H&E staining and MTS showed that renal fibrosis was markedly reduced in renal tissues of *Irf5^fl/fl^Lyz2^Cre^* UUO mice (Figure 7, A and B). IHC staining clarified the decrease of CD68^+^ cells in the obstructive kidney tissue between the *Irf5^fl/fl^* mice and *Irf5^fl/fl^Lyz2^Cre^* mice (Figure 8, A and B). The decreased expression of α-SMA and VIM in renal tissues of *Irf5^fl/fl^Lyz2^Cre^* UUO mice was verified by qPCR and WB (Figure 7, C–E). We also found both inflammatory cell infiltration and inflammatory factors were significantly decreased in renal tissues of *Irf5^fl/fl^Lyz2^Cre^* UUO mice, as demonstrated by qPCR and further validated at the protein level by WB (Figure 8, C and D).

We next performed RNA-seq on renal fibrosis tissues from *Irf5^fl/fl^Lyz2^Cre^* UUO and *Irf5^fl/fl^* UUO mice. The differentially expressed genes were associated with immune response and ECM (Figure 8, E and F, and Supplemental Tables 7 and 8).

### Myeloid-specific Irf5 deletion reduced MMP9 expression in kidneys of UUO mice.

The RNA-Seq analysis further revealed that the most members of the matrix metalloproteinase (MMP) family were positively correlated with *Irf5*. Notably, *Mmp9* showed the most substantial upregulation, with a log_2_ fold change (log_2_FC) of 3.37 from WT UUO and WT sham mice renal tissue (Supplemental Table 3). We further examined the expression of MMP9 from the sham and UUO groups of *Irf5^fl/fl^* and *Irf5^fl/fl^Lyz2^Cre^* mice using immunofluorescence (Figure 9A). The results showed that the number of MMP9^+^ cells in renal tissues of the *Irf5^fl/fl^Lyz2^Cre^* group was significantly fewer than in the *Irf5^fl/fl^* UUO group (Figure 9C). Consistently, the protein and mRNA levels of MMP9 were significantly lower in the renal tissues of *Irf5^fl/fl^Lyz2^Cre^* UUO mice compared with the *Irf5^fl/fl^* UUO mice (Figure 9, D–F). Furthermore, immunofluorescence results indicated that IRF5 and MMP9 colocalized in the tubulointerstitial regions of the *Irf5^fl/fl^* UUO mice. Additionally, the percentage of IRF5^+^MMP9^+^ cells among MMP9^+^ cells was significantly increased in renal tissues of the *Irf5^fl/fl^* UUO mice compared with *Irf5^fl/fl^* sham mice (Figure 9, B and C).

### IRF5 positively regulated MMP9 and inflammatory responses in bone marrow–derived macrophages.

Our previous report showed that IRF5 directly regulate MMP9 in response to CpG, a TLR9 agonist (14). Similarly, we observed a significant increase in the expression of IRF5 and MMP9 in BMDMs after stimulation with TNF-α, with colocalization within the macrophages (Figure 10A). IRF5 KO in macrophages was verified by Western blotting (Supplemental Figure 2C). The expression of MMP9 in *Irf5^fl/fl^Lyz2^Cre^* BMDMs was significantly lower than those in control (Figure 10B). Likewise, we found that TNF-α stimulation significantly upregulated *Mmp9* mRNA and MMP9 protein expression in BMDMs, and the increase was markedly less in *Irf5^fl/fl^Lyz2^Cre^* BMDMs (Figure 10, C and D). In addition, qPCR was performed to analyze BMDMs derived from *Irf5^fl/fl^* and *Irf5^fl/fl^Lyz2^Cre^* mice. The results demonstrated that mRNA expression of the proinflammatory factors *Il1b*, and *Il6* was significantly upregulated in both groups after stimulation, but the increase was considerably lower in *Irf5^fl/fl^Lyz2^Cre^* BMDMs. Although TNF-α stimulation appeared to slightly upregulate profibrotic factor *Tgfb1* expression in BMDMs, the difference did not reach statistical significance between the 2 groups (Figure 10C). CUT&Tag analysis of TNF-α–treated BMDMs demonstrated that IRF5 binding intensity at the *Mmp9* promoter region was significantly enhanced compared with controls (Figure 10E). These findings suggest that IRF5 directly regulates *Mmp9* transcription and may promote fibrosis through the MMP9 pathway rather than the classical TGF-β1 pathway.

### MMP9 expression was significantly increased and colocalized with IRF5 in the renal tissues of patients with obstructive nephropathy.

We examined the expression and colocalization of IRF5 and MMP9 in the renal tissues of patients with obstructive nephropathy. The results indicate that the increased IRF5 and MMP9 expression was associated with the severity of renal fibrosis; their colocalization was evident (Figure 11A), and the proportion of IRF5^+^MMP9^+^ cells among MMP9^+^ cells was significantly higher (Figure 11B). This suggests that IRF5 plays a crucial role in the progression of renal fibrosis via the regulation of MMP9 expression.

### IRF5 inhibitor attenuated UUO induced renal fibrosis in mice.

In order to further investigate the role of IRF5 in promoting renal fibrosis, we established the UUO mouse model in WT mice. The treatment group received i.p. injections of IRF5 inhibitor (100 μg per mouse) on days 0, 1, 4, and 7 after UUO induction. An equal volume of saline was administered to the control group. Mice were sacrificed on day 14, and kidney samples were collected (Figure 12A). Western blot results confirmed the inhibitory effect on IRF5 (Supplemental Figure 2D). In the results of MTS, the fibrotic area of the obstructed kidneys from the treatment group was significantly smaller compared with the control group (*P* = 0.02). IHC staining showed a significant decline in the expression of α-SMA and VIM in the obstructed kidneys from the treatment group (Figure 12, B and C). Additionally, the numbers of IRF5^+^ cells and CD68^+^ cells were significantly decreased in the obstructed kidneys from the treatment group (Figure 12, B and D).

To investigate the effects of the IRF5 inhibitor on the expression of multiple proinflammatory and profibrotic factors, we subsequently conducted qPCR analyses of fibrosis-related indicators (*Acta2*, *Mmp9*, and *Vim*) and proinflammatory markers (*Tnf-α*, *Tgf-β1*, *IL-6*, and *IL-1β*) on the obstructed kidneys. Interestingly, our results demonstrated that *Acta2*, *Mmp9*, *Vim*, *Tnf-α*, *IL-6*, and *IL-1β* levels significantly decreased in the treatment group, with no significant difference observed in *Tgf-β1* expression between the 2 groups (Figure 12E). Furthermore, it showed that the protein expression levels of MMP9 and α-SMA were significantly lower in the obstructed kidneys from the treatment group (Figure 12F).

## Discussion

In the current study, we found that both IRF5 expression and the number of IRF5^+^CD68^+^ cells were significantly elevated in the renal tissues from patients with obstructive nephropathy and mice with UUO. Notably, the number of IRF5^+^CD68^+^ cells was positively correlated with the severity of renal fibrosis. Then we demonstrated that global and myeloid-specific IRF5 deficiency markedly reduced renal fibrosis, immune cell infiltration, and the expression of immune mediators following ureteral obstruction. We further revealed that myeloid-specific IRF5 deficiency modulated fibrosis-associated pathways, with IRF5 acting as a positive regulator of *Mmp9* expression. The application of IRF5 inhibitors could alleviate renal fibrosis in mice and inhibit the expression of proinflammatory factors. Finally, we also observed significantly increased MMP9 expression colocalizing with IRF5 in the patients renal tissues. Collectively, our findings suggest that macrophage IRF5 promotes renal fibrosis by regulating MMP9 expression in obstructive nephropathy.

The role of IRF5 in various fibrotic diseases has garnered significant attention in recent years. A previous study has shown that IRF5 plays a pivotal role in pulmonary fibrosis by modulating macrophage polarization (15). Similarly, another study showed that IRF5 promotes the progression of liver fibrosis by regulating inflammatory macrophages (16). These findings suggest that IRF5 may have a crucial function in the formation of fibrosis. Consistent with these studies, our experimental results show that IRF5 plays a key role in the progression of renal fibrosis in obstructive nephropathy by modulating macrophages.

Numerous studies have demonstrated that IRF5 is not only a key regulatory factor in the inflammatory response but also actively participates in inflammation by modulating the transcription of target genes in various myeloid-derived cells (17–21). Previous study has shown that IRF5 regulates liver inflammation and fibrosis by mediating inflammatory activation, including activation of macrophages and secretion of proinflammatory factors (11). IRF5 can enhance the expression of proinflammatory cytokines such as IL-6, TNF-α, and CXCL10 by directly binding to their promoter regions and forming a complex with RelA, a subunit of NF-κB (22). In our study, we observed that myeloid-specific IRF5 deficiency in UUO mice resulted in a significant reduction in the number of CD68^+^ macrophages and CD3^+^ T cells in renal tissues, alongside a marked decrease in expression of proinflammatory cytokines. These findings demonstrate a critical role for deficiency of IRF5 in macrophages, through attenuating the recruitment and activation of inflammatory cells, suppressing the inflammatory response, and reducing the expression of proinflammatory factors. This ultimately leads to a mitigation of inflammation-driven fibrotic processes.

During the development of fibrosis, macrophages contribute not only by initiating inflammatory response but also by promoting ECM deposition and tissue remodeling (23). Previous study has identified a macrophage subset associated with ECM remodeling during the transition from acute kidney injury to CKD through spatial single-cell transcriptomic analysis. These macrophages, primarily derived from infiltrating monocytes, were found to be closely linked to the progression of fibrosis (24). Consistent with the profibrotic role of macrophages, our in vitro data demonstrate that IRF5-deficient BMDMs exhibit significantly reduced MMP9 expression upon stimulation, suggesting that IRF5 may contribute to ECM remodeling in part through the regulation of macrophage-derived MMP9.

As a zinc-dependent endopeptidase, MMP9 is known to regulate the degradation and remodeling of ECM (25) and play a role in many physiological processes, including tissue repair, inflammation, and the pathological process of fibrosis (26–31). While MMP9 plays a complex role in fibrosis generally, the current understanding, particularly focusing on kidney tissue, is that it predominantly promotes fibrogenesis. This profibrotic role has been validated in multiple animal models, including CKD and UUO, as well as in vitro cell experiments (32, 33). Previous studies have demonstrated that MMP9 promotes fibrosis by inducing epithelial/mesenchymal transition (EMT) in renal tubular cells (34, 35). MMP9 can directly induce EMT in endothelial cells via the Notch signaling pathway. This process is characterized by the loss of the endothelial marker CD31 and a significant upregulation of mesenchymal markers such as α-SMA and VIM, leading to their transformation into myofibroblasts. Conversely, the deletion of MMP9 can attenuate this process (36). It has been established that osteopontin mediates macrophage infiltration. MMP9, derived from both renal tubular epithelial cells (TECs) and macrophages, extensively cleaves osteopontin, which in turn exacerbates macrophage recruitment and promotes the progression of EMT in TECs, ultimately increasing the severity of renal fibrosis (37). MMP9 can also promote collagen deposition by activating and releasing the core profibrotic factor TGF-β, which in turn stimulates TGF-β–dependent fibroblast activation (38). Furthermore, MMP9 contributes to the accumulation of neutrophils in the fibrotic kidney by elevating inflammatory cytokine levels and promoting cell recruitment, thereby forming a vicious cycle that exacerbates fibrosis (39). Collectively, substantial evidence indicates that macrophage-derived MMP9 promotes renal fibrosis through multiple mechanisms, both directly via EMT induction and indirectly through immune cell recruitment and inflammatory amplification.

Previous studies have shown that small molecule IRF5 inhibitors can impede the disease progression of systemic lupus erythematosus (SLE) and contribute to maintaining the remission phase in model mice (40). Additionally, other investigations have verified that IRF5 inhibitors can mitigate the myocardial inflammatory response and fibrosis in model mice of sclerosis (41). Extending these findings, our study demonstrated that IRF5 inhibitor can also significantly attenuate renal fibrosis in the mouse UUO model and suppress the expression of fibrotic factors and proinflammatory factors. These findings collectively suggest that IRF5 inhibitors could inhibit inflammatory responses and slow the fibrotic process, thereby exhibiting the potential to serve as targeted drugs for the treatment of fibrosis diseases.

In summary, we validated by CUT&Tag that IRF5 directly binds to the MMP9 promoter. In vivo studies using UUO models of global and myeloid-specific IRF5 deficiency mice, as well as IRF5 inhibitor-treated WT mice, all demonstrated significantly reduced MMP9 expression and diminished macrophage recruitment in vivo. In vitro, primary BMDMs from IRF5-deficient mice showed markedly lower MMP9 expression upon stimulation. These findings indicate that IRF5 mediates the progression of obstructive nephropathy by directly regulating MMP9 expression, and that targeting IRF5 may represent a potential therapeutic strategy for alleviating renal fibrosis.

## Methods

### Sex as a biological variable.

Male C57BL/6J mice were utilized in this study because male animals exhibited less variability in phenotype. To minimize sex-based variability, only male mice were subjected to the UUO model. We acknowledge that it is unknown whether the findings are relevant for female mice.

### Human kidney samples.

Renal tissue specimens were included based on the following criteria: (a) Renal Fibrosis Group, which included surgically excised renal parenchymal tissue from patients with urethral obstruction, and (b) Control Group, which included renal parenchymal tissue without fibrotic changes, obtained from areas adjacent to renal tumors without prior chemotherapy, or from tissue resected due to trauma. Exclusion criteria were the following: (a) samples from patients with polycystic kidney disease, dysplastic kidney disease, secondary renal fibrosis, immune deficiencies, viral hepatitis, or other related conditions and (b) samples from patients with incomplete clinical data. Detailed characteristics of the patients are provided in Supplemental Tables 1 and 2.

The extent of fibrosis was defined as the ratio between fibrotic regions and total tissue area, categorized as follows: 10%–25% for mild, 26%–50% for moderate, and > 50% for severe fibrosis. Inflammation was blindly scored on the degree of inflammatory cell infiltration: < 25% was defined as mild, 25%–50% as moderate, and > 50% as severe.

### Animal model.

All male C57BL/6 mice were housed in the specific pathogen–free conditions of Zhejiang Chinese Medical University Laboratory Animal Research Center. Mice were housed under standard conditions with controlled temperature (21°C–25°C), humidity (40%–60%), and 12/12-hour dark-light cycle. *Irf5*-KO mice were purchased from Nanjing BioMedical Research Institute of Nanjing University, China (Supplemental Data, Certificate 1). *Irf5^fl/fl^* mice and *Lyz2^Cre^* mice were purchased from The Jackson Laboratory (Supplemental Data, Certificates 2 and 3). Myeloid cell–specific deficiency *Irf5* mice (*Irf5^fl/fl^ Lyz2^Cre^*) were created by crossing the *Irf5^fl/fl^* mice with the *Lyz2^Cre^* transgenic mice. Littermates not carrying the *Lyz2^Cre^* transgene (*Irf5^fl/fl^*) served as controls. All mice were maintained on a C57BL/6 background, and genotypes were determined by PCR using tail DNA.

### UUO model.

The UUO procedure was performed according to the method described by Chevalier et al. (13). Briefly, under isoflurane anesthesia, the left ureter was exposed and ligated at 2 distinct points with 1 mm interval between the ligatures. Sham-operated mice had their ureters exposed, manipulated but not ligated. The mice were euthanized at designated time points after the UUO procedure, and both obstructed and nonobstructed contralateral kidneys were harvested for RNA, protein extraction, and histopathology.

### N5-1 treatment.

Eight-week-old male C57BL/6 mice were subjected to UUO or sham surgery. Following the procedures, the mice received injections of either the N5-1 (an *Irf5* inhibitor) 100 μg per mouse per injection (42) or a physiological saline control on postoperative days 0, 1, 4, and 7. The N5-1 peptide was previously described by Song et al. (42). N5-1 was synthesized by Beyotime Biotech Inc. (Supplemental Data, Certificate 4). The animals were euthanized 14 days after surgery and the kidneys were collected for further analysis.

### Cell culture.

Mouse primary bone marrow–derived macrophages (BMDMs) were prepared by flushing the bone marrow from the tibiae and femoral obtained from 6- to 8-week-old *Irf5^fl/fl^* and *Irf5^fl/fl^Lyz2^Cre^* mice. Cells were cultured in the complete medium High-glucose Dulbecco’s modified Eagle’s medium (DMEM; Gibco) containing 10% FBS (Procell), 1% penicillin/streptomycin solution (Biosharp), and 20 ng/mL macrophage colony-stimulating factor (M-CSF; Pricella). After 7 days, the complete medium was discarded, and serum-free high-glucose DMEM was added. For macrophage activation, 30 ng/mL TNF-α (MedChemExpress) was added in culture medium to treat for 8 hours, 24 hours, and 48 hours.

### Histological analysis.

Both human and murine kidney tissues were fixed with 4% paraformaldehyde, embedded in paraffin and sectioned at 4 μm. Following deparaffinization, the sections were stained with routine H&E staining and Masson trichrome staining (MTS) procedures. For IHC, sections were deparaffinized and treated with 3% hydrogen dioxide. After antigen retrieval and blocking, sections were incubated with primary antibodies for 90 minutes at room temperature (RT) followed by corresponding secondary antibody incubation for 40 minutes at RT and DAB staining. For immunofluorescence staining, sections were first deparaffinized and rehydrated. After antigen retrieval and blocking, sections were incubated with primary antibodies for 120 minutes at RT followed by corresponding secondary antibody incubation for 1 hour at RT. Following reagents were used to detect secondary antibodies: FITC (ZSGB-BIO, catalog ZF-0312), TSA-CY3 (Pinuofei Biological, catalog PN0106), and TSA-594 (Pinuofei Biological, catalog PN0106). Nuclei were stained with DAPI (Pinuofei Biological) at RT for 5 minutes. All sections were observed under a fluorescence microscope (Leica) or a laser scanning confocal microscopy (Leica). For the quantitative analysis, 10 × 400 fields were randomly selected in the cortical area from each kidney section. All antibodies used are listed in Supplemental Table 9.

Given that IRF5 and MMP9 are key molecules in this study, the specificity of both antibodies was validated in kidney sections by immunofluorescence staining, as shown in Supplemental Figures 3 and 4.

All histological staining and semiquantitative analyses were performed. All slides were coded with random numbers by a researcher not involved in the analysis, and we performed the analyses without any knowledge of the group assignments.

### Immunocytochemistry.

For immunocytochemistry (ICC), cells were also fixed in 4% paraformaldehyde for 20 minutes at 4°C prior to permeabilizing and blocking in 0.02% Triton X–100/2% BSA (Solarbio)/PBS (Pinuofei Biological) for 15 minutes. Cells were incubated with primary antibodies for 90 minutes at 37°C followed by incubation with secondary antibodies for 40 minutes at 37°C. Mounting was performed with DAPI and microscopic examinations with a fluorescence microscope (Leica). All antibodies used are listed in Supplemental Table 9.

### Western blot analysis.

Kidney tissues and cells were harvested and lysed in RIPA lysis buffer (MeilunBio) containing protease inhibitors (MeilunBio). An equal amount of protein from all the samples was separated by SDS-PAGE (Servicebio) and then transferred to PVDF membranes (MilliporeSigma). Membranes were incubated with primary antibodies at 4°C overnight and subsequently incubated with corresponding secondary antibodies for 1 hour at RT. Finally, the protein bands were visualized by chemiluminescence and analyzed using ImageJ software (NIH; Version win64-bit java8). All antibodies used are listed in Supplemental Table 9.

### qPCR.

Total RNA was extracted from kidney tissues and reverse-transcribed into cDNA using PrimeScript RT Master Mix (Takara). And qPCR was performed using TB Green Premix Ex Taq (Takara). The relative gene expression was determined after normalizing the data to the β-actin gene and calculating the ΔΔCT. All primer sequences used are listed in Supplemental Table 10.

### RNA-seq and bioinformation analysis.

Harvested tissues were incubated in TRIzol reagent (Invitrogen). Both RNA isolation and RNA-seq were performed by Novogene Co. Ltd. Briefly, RNA concentration was assessed by Qubit 2.0 fluorometer (Life Technologies). The quality was verified by the Bioanalyzer 2100 system (Agilent Technologies). Libraries were created using NEBNext Ultra RNA Library Prep Kit for Illumina (New England Biolabs) according to the manufacturer’s instructions. Clean reads were mapped to the mouse genome (GRCm38) (https://ftp.ensembl.org/pub/release-94/fasta/mus_musculus/dna/Mus_musculus.GRCm38.dna.primary_assembly.fa.gz), (annotation link, https://ftp.ensembl.org/pub/release-94/gtf/mus_musculus/Mus_musculus.GRCm38.94.gtf.gz) using STAR v2.5.1b (https://github.com/alexdobin/STAR). Feature Counts (v1.5.0) was used to count reads that mapped to the entire gene body of genes. Differential gene expression analysis was performed using DEseq2 (v1.20.0). Genes with a *P* value (FDR corrected) less than 0.05 and log_2_ fold change greater than 1 or less than 1 were considered significantly differentially expressed. The list of transcription factors is available in an online database (https://guolab.wchscu.cn/static/AnimalTFDB3/download/Mus_musculus_TF).

The raw RNA-seq data have been deposited in the NCBI Gene Expression Omnibus (GEO) database under accession no. GSE330552. The data are currently private and will be made publicly available upon publication of this manuscript.

### CUT&Tag sequencing.

CUT&Tag experiments were carried out by Tianjin Novogene Bioinformatic Technology Co. Ltd. (https://cn.novogene.com/). Permeabilized with digitonin, cells underwent antibody-guided (anti-IRF5 antibody: Abcam, ab21689) (Control antibody: IgG, Abcam ab172730) pA-Tn5 transposase-mediated tagmentation using the CUT&Tag_Novogene Kit, simultaneously ligating adapters for PCR library construction. Post-PCR libraries were purified with AMPure beads and quality-controlled via Agilent Bioanalyzer 2100. Sequencing libraries were clustered on cBot and processed through fastp (version 0.20.0) for clean data generation. Alignment to reference genomes (BWA version 0.7.12) preceded MACS2 (version 2.1.1) peak calling (*q* < 0.05). HOMER (version 4.9.1) performed motif analysis on 500 bp summit-centered peaks, while ChIPseeker annotated peak-gene associations. Differential peaks (fold change > 2 RPM) were identified using bedtools-merged peaks across groups.

### Statistics.

All statistical analyses were performed using Prism Version 10.1.2 software (GraphPad Software). Experimental data were presented as mean ± SD from at least 3 independent experiments. One-way ANOVA followed by Tukey’s post hoc test was used for comparisons among 3 or more independent groups, and an unpaired 2-tailed Student’s *t* test was used when comparing only 2 unpaired groups. Spearman correlation analysis was used to determine the significance of correlation analysis. *P* value less than 0.05 was considered statistically significant in all analyses.

### Study approval.

This study was conducted in accordance with the Declaration of Helsinki, and was approved by the Ethics Committee of the Children’s Hospital, Zhejiang University School of Medicine (no. 2023-IRBAL-081). Written informed consent was obtained from all the patients and their guardian. All patient information is anonymized to protect personal privacy.

### Data availability.

We are committed to the principles of open and reproducible research and, thus, are dedicated to sharing the data underlying this study. All data generated or analyzed during this research are openly available. The raw RNA-seq data generated in this study have been submitted to the NCBI GEO database under accession no. GSE330552. Data are currently private and will be made publicly available upon publication of the manuscript. All Supporting data values for the figures in this study are available in the Supporting Data Values file.

## Author contributions

Co–first authors are JW and GD. The order of authorship was determined by the extent of their contributions to the study: JW conceived and designed the study, acquired funding, and supervised the research. GD performed all UUO surgeries, conducted the majority of cell experiments, performed more than half of the Western blotting and qPCR assays, prepared all figures, and handled all revisions and correspondence during the resubmission process. Both authors contributed substantially to the work, and the order was agreed upon by all authors. Conception and design were contributed by JW. All pathological histological section staining and semiquantitative analyses were performed by MY. Experimental operation was contributed by GD, ZL, MY, ZX, and ZY. Data acquisition was contributed by JW, GD, ZL, MY, TC, ZY, and YZ. Data analysis and interpretation were contributed by JW, GD, ZL, MY, TC, and YZ. Manuscript drafting was contributed by JW, MY, GD, and TC. Critical revision of the manuscript was contributed by JW, and XY. Statistical analysis was contributed by JW, GD, ZL, MY and TC. Figure design was contributed by JW, GD, ZL, and TC. Supervision was contributed by JW and XY.

## Conflict of interest

The authors have declared that no conflict of interest exists.

## Funding support

- National Natural Science Foundation of China (82371709) (JW).
- Key Project of Zhejiang Provincial Natural Science Foundation (LHDMZ25H050002) (JW).

## Supplementary Material

Supplemental data

Unedited blot and gel images

Supplemental table 1

Supplemental table 10

Supplemental table 2

Supplemental table 3

Supplemental table 4

Supplemental table 5

Supplemental table 6

Supplemental table 7

Supplemental table 8

Supplemental table 9

Supporting data values

## Figures and Tables

**Figure 1:**
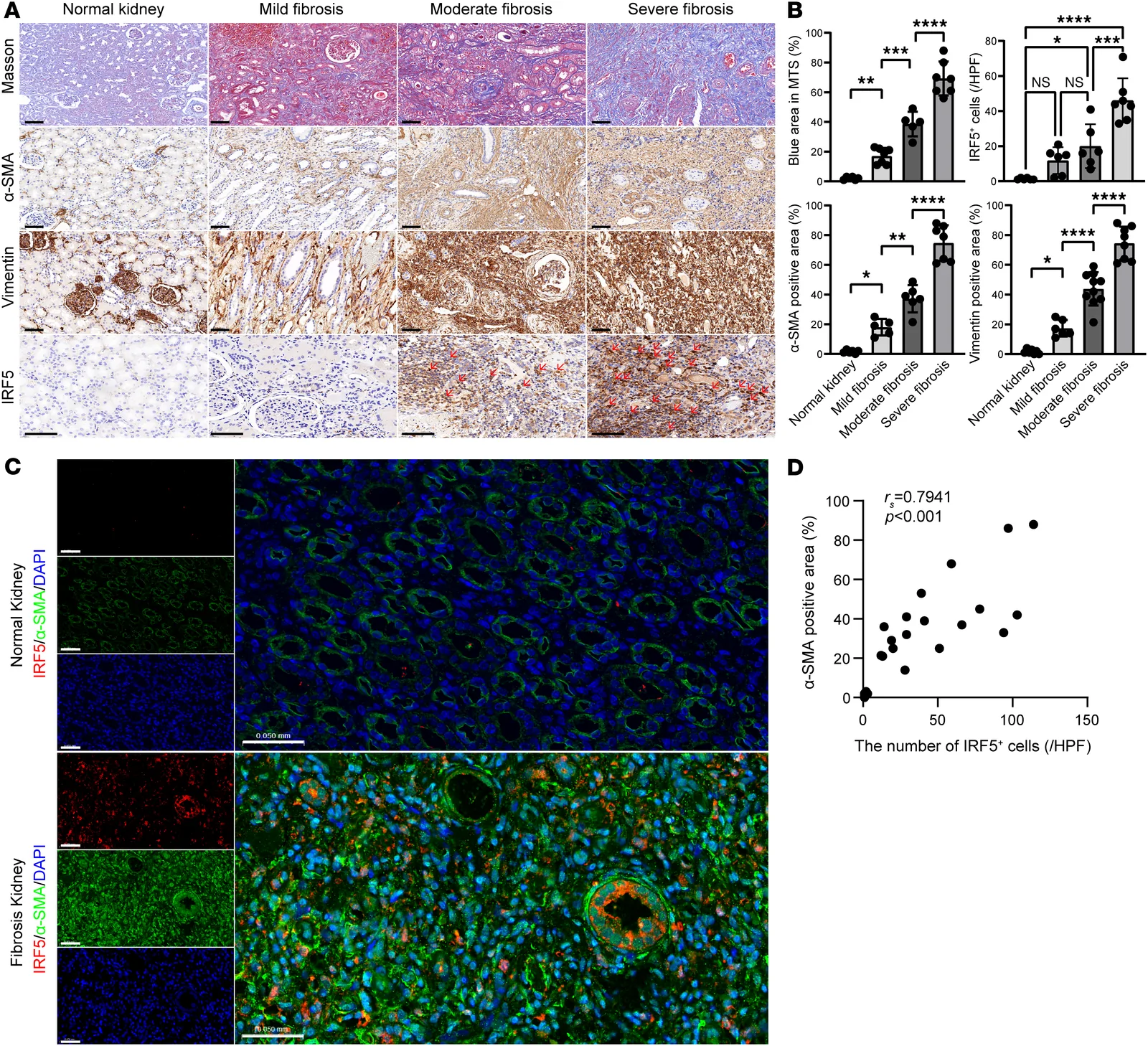
IRF5 was increased in human renal tissues with obstructive nephropathy. (**A**) Masson trichrome staining and IHC staining for α-SMA, VIM, and IRF5 in kidney tissues with varying degrees of fibrosis. Typical images and quantification are included. Arrows indicate strongly positive cells exhibiting macrophage-like nuclei under staining. Scale bars: 100 μm. (**B**) With the progression of fibrosis, collagen deposition, α-SMA^+^ and VIM^+^ areas gradually increased. Semiquantitative population of IRF5^+^ cells in kidneys with varying degrees of fibrosis. The number of IRF5^+^ cells increased in fibrotic tissues. (**C** and **D**) Representative images of immunofluorescence (IF) using IRF5 (red), α-SMA (green), and DAPI (blue) with normal kidney and fibrosis kidney. Scale bars: 50 μm. Spearman correlation analysis showed that the number of IRF5^+^ cells was significantly positively correlated with the expression of α-SMA (*r_s_* = 0.7941, *P* < 0.001). Data are expressed as the mean ± SD. Statistical comparisons among multiple groups were performed using 1-way ANOVA followed by Tukey’s post hoc test. *n* = 3–10. *P < 0.05; **P < 0.01; ***P < 0.001; ****P < 0.0001. HPF, high-power field.

**Figure 2:**
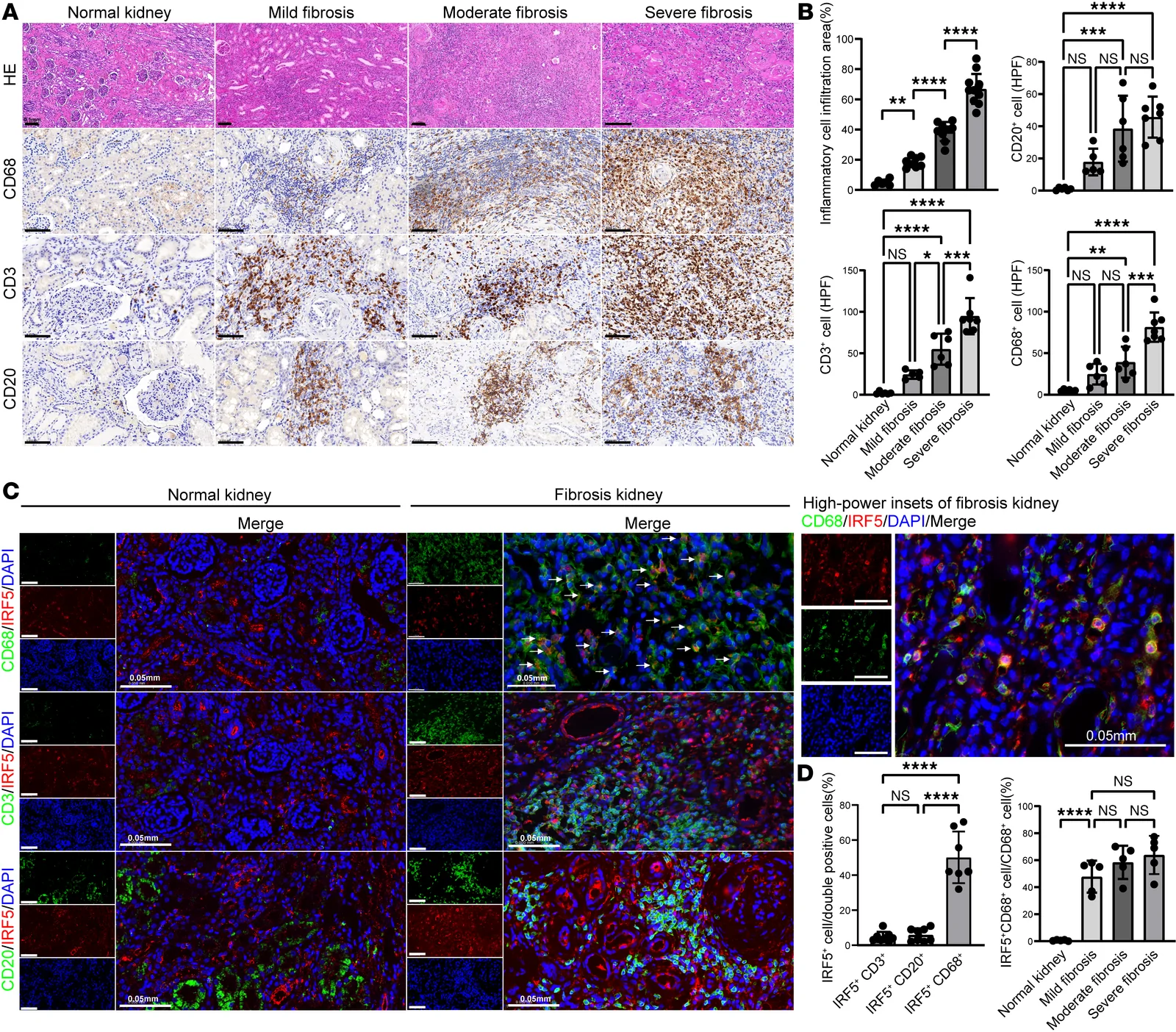
Elevated IRF5^+^CD68^+^ cell counts in renal tissues of patients with obstructive nephropathy. (**A** and **B**) Histologic images of kidney sections with varying degrees of fibrosis stained with H&E and IHC staining for CD68, CD20, and CD3. Typical images and quantification are included. Scale bars: 100 μm. (**C**) Representative images of normal and fibrosis kidney immunofluorescence using IRF5 (red), CD68/CD20/CD3 (green), and DAPI (blue). Representative high-power insets images of fibrosis kidney immunofluorescence using IRF5 (red), CD68 (green), and DAPI (blue). Scale bars: 50 μm. White arrows indicated double positive cells (colocatable cells). Randomly selected 10 fields of each patient’s kidney under 400× magnification were scored for quantification. (**D**) Ratio of IRF5^+^ cells to double-positive cells and ratio of IRF5^+^CD68^+^ cells in kidney sections with varying degrees of fibrosis. Data are expressed as the mean ± SD. Statistical comparisons among multiple groups were performed using 1-way ANOVA followed by Tukey’s post hoc test. *n* = 3–10. *P < 0.05; **P < 0.01; ***P < 0.001; ****P < 0.0001. HPF, high-power field.

**Figure 3:**
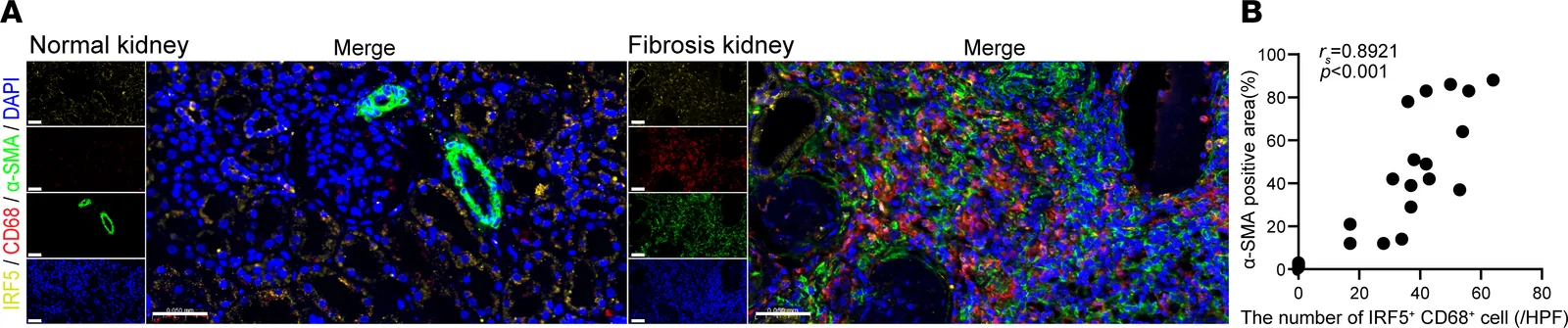
IRF5 localizes in macrophages and is associated with renal fibrosis. (**A** and **B**) Representative images of normal and fibrosis kidney immunofluorescence using IRF5 (yellow), CD68 (red), α-SMA (green), and DAPI (blue). Scale bars: 50 μm. The number of IRF5^+^CD68^+^ cells was significantly positively correlated with the α-SMA^+^ areas (*r_s_* = 0.8921, *P* < 0.001).

**Figure 4:**
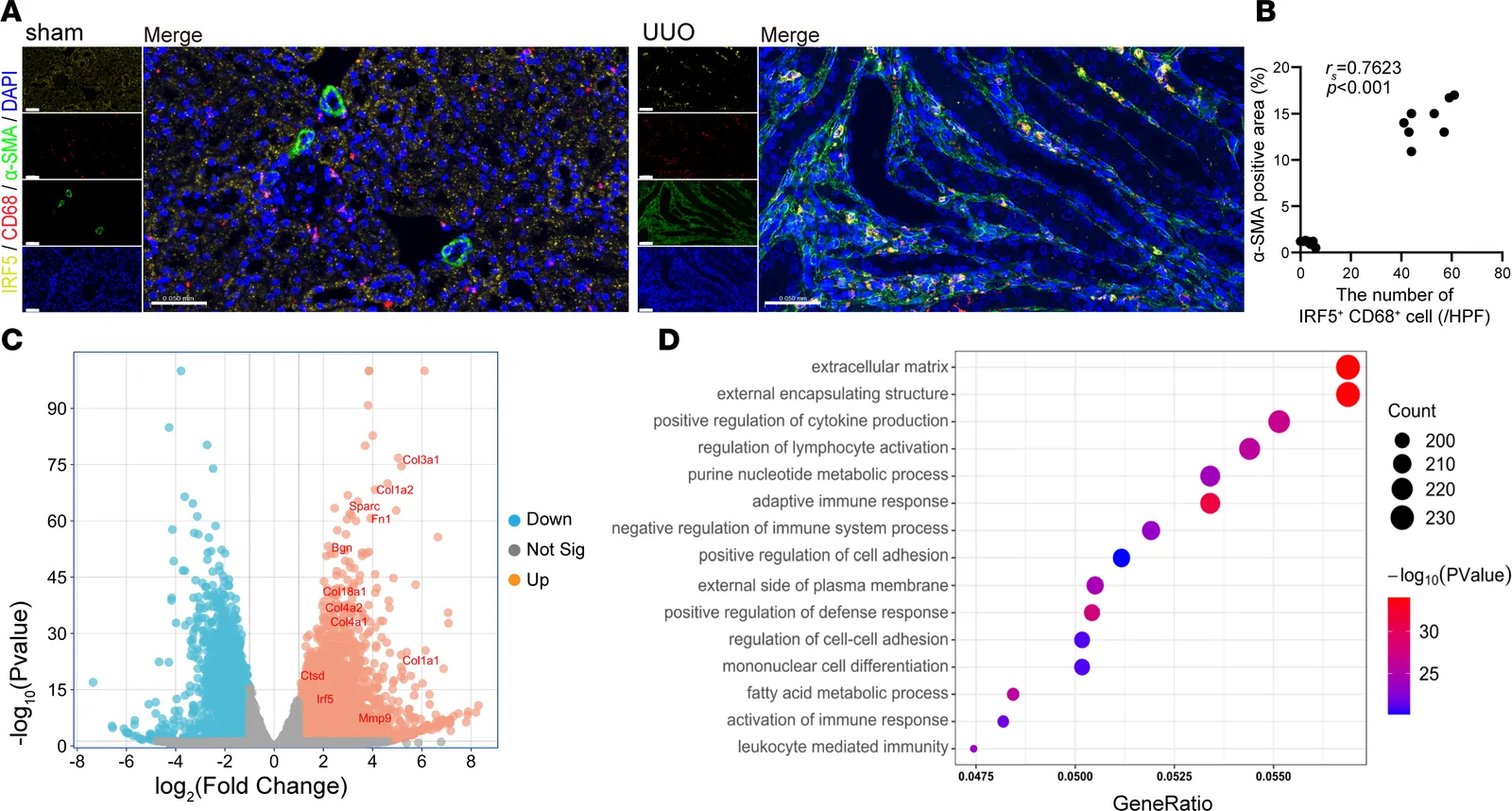
IRF5^+^CD68^+^ cell expression markedly increases in UUO mouse renal tissues. (**A**) Representative images of sham and UUO immunofluorescence using IRF5 (yellow), CD68 (red), α-SMA (green), and DAPI (blue). Scale bars: 50 μm. (**B**) The number of IRF5^+^CD68^+^ cells was significantly positively correlated with the α-SMA^+^ areas (*r_s_* = 0.7623, *P* < 0.001). (**C**) Gene expression difference of WT sham and WT UUO mice. (**D**) Gene ontology (GO) enrichment analysis of WT sham and WT UUO mice. The highly enriched categories include the extracellular matrix (ECM), external encapsulating structure, positive regulation of cytokine production, and regulation of lymphocyte activation.

**Figure 5:**
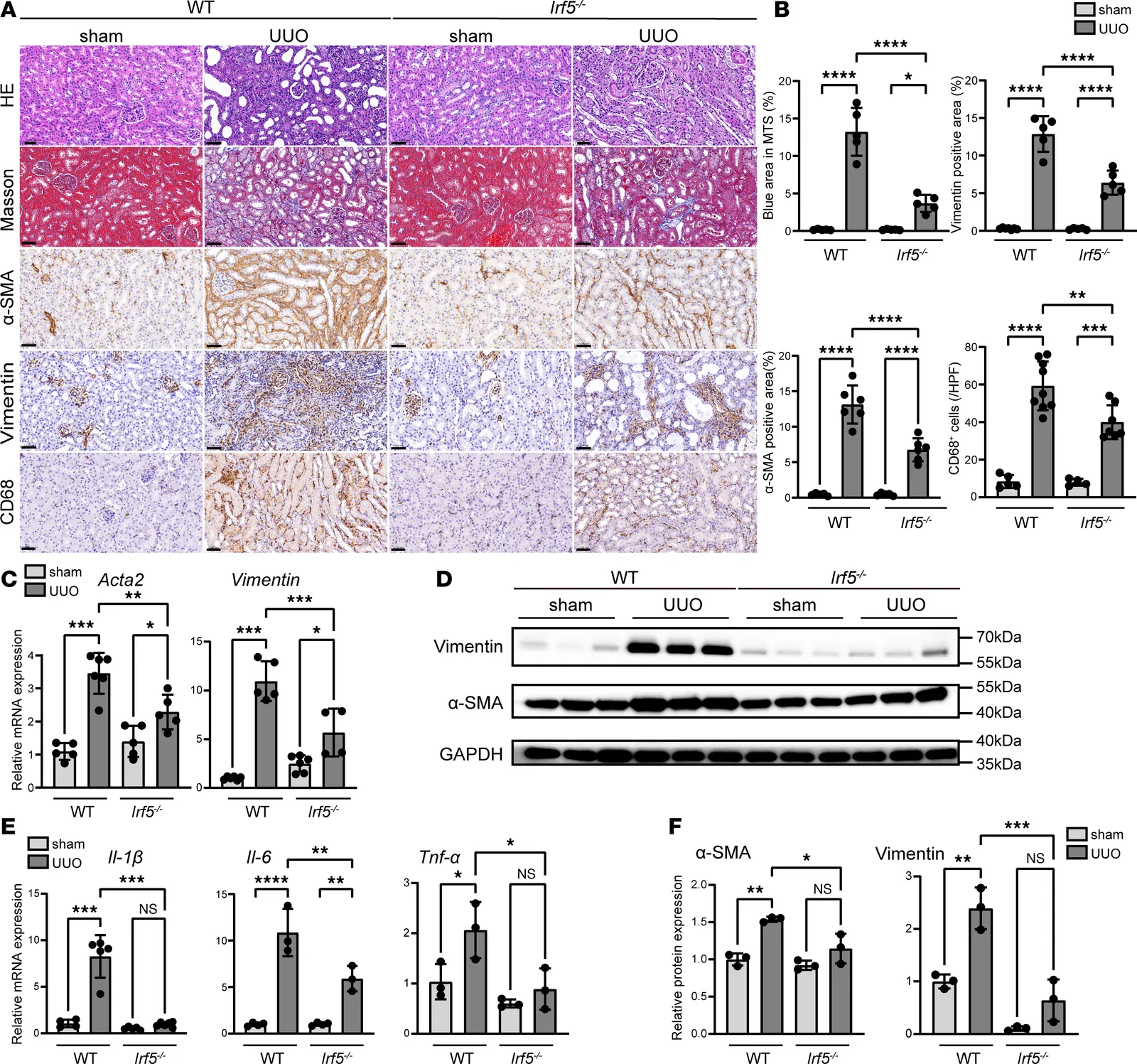
IRF5 KO mitigates inflammation and fibrosis in the UUO mouse model. (**A**) Representative H&E, masson staining, and IHC images of α-SMA, VIM, and CD68 of WT mice and *Irf5*^–/–^ mice in sham group and UUO group. Scale bars: 50 μm. (**B**) Collagen deposition is increased in UUO-induced WT renal tissues yet decreased in *Irf5*^–/–^ mice. VIM^+^α-SMA^+^ area was significantly upregulated in the UUO-induced WT renal tissues and downregulated in the *Irf5*^–/–^ renal tissues. The number of CD68^+^ cells is markedly reduced in UUO-induced *Irf5*^–/–^ renal tissues compared with WT renal tissues. (**C**) Renal mRNA expression of *Acta2* and *Vim* in the indicated mouse groups were measured by qPCR. (**D** and **F**) Renal protein expression of α-SMA and VIM in the indicated mouse groups were measured by Western blot, the results were subjected to quantitative analysis. (**E**) Renal mRNA expression of *Il-1β*, *Il-6*, and *Tnf-α* in the indicated mouse groups were measured by qPCR. The mRNA expression level was reduced in the UUO-induced *Irf5*^–/–^ renal tissues. Data are expressed as the mean ± SD. Statistical comparisons among multiple groups were performed using 1-way ANOVA followed by Tukey’s post hoc test. *n* = 3–9. *P < 0.05; **P < 0.01; ***P < 0.001; ****P < 0.0001.

**Figure 6:**
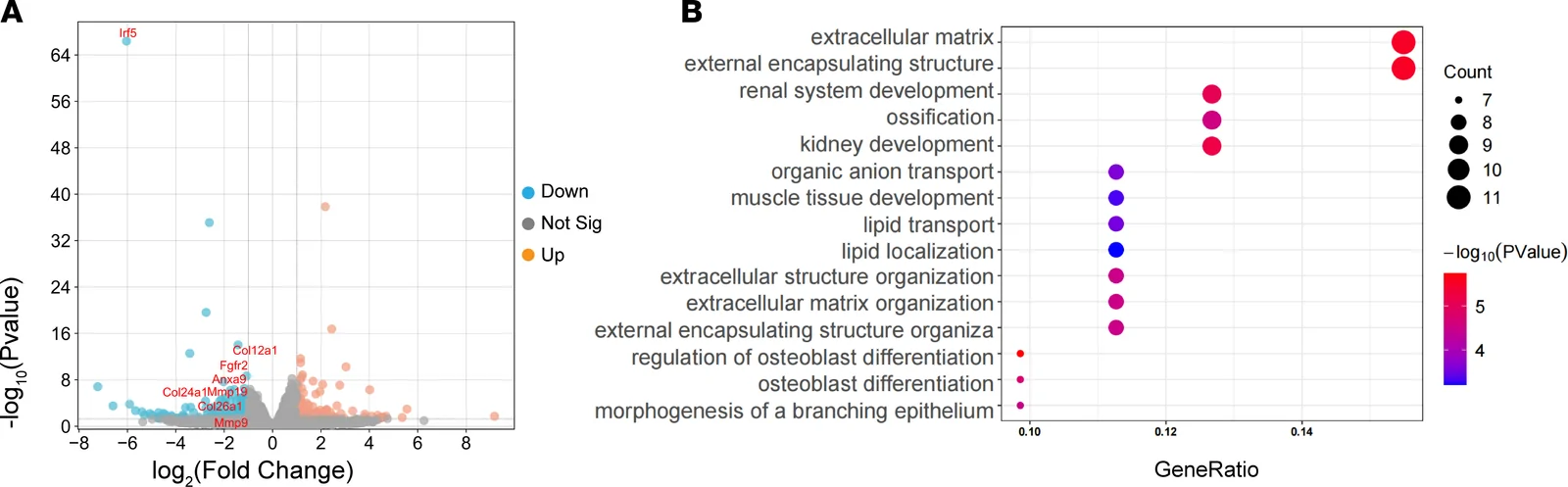
*Irf5*-KO mice display differential gene expression in RNA-seq analysis. (**A**) Gene expression difference of WT UUO and *Irf5*^–/–^ UUO mice. (**B**) GO enrichment analysis of WT UUO and *Irf5*^–/–^ UUO mice. The highly enriched categories include the extracellular matrix (ECM), external encapsulating structure, and renal system development.

**Figure 7:**
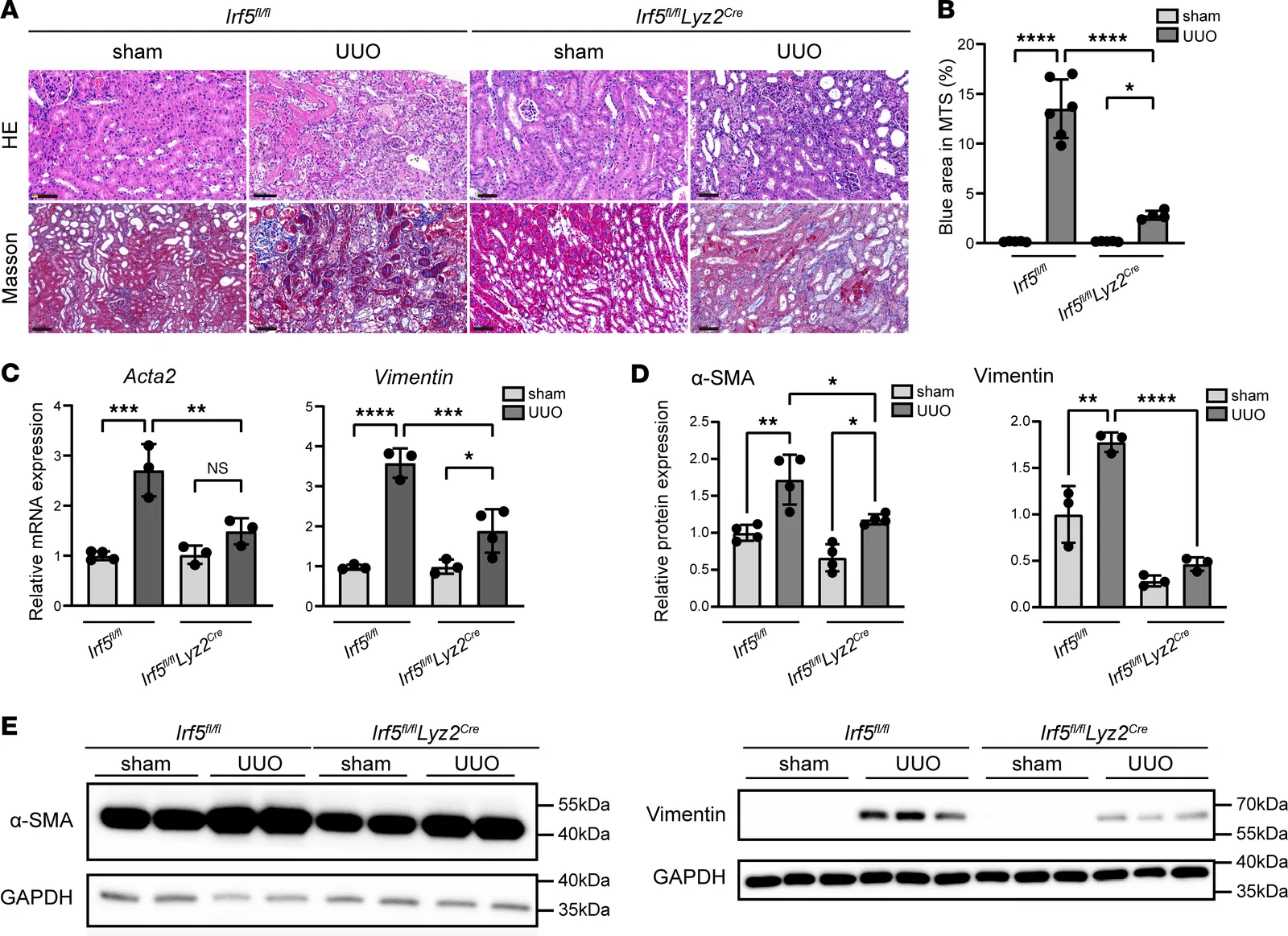
IRF5 deficiency in myeloid cells inhibits renal fibrosis in vivo. (**A**) Representative H&E and Masson staining images of *Irf5^fl/fl^* mice and *Irf5^fl/fl^Lyz2*^Cre^ mice in sham-operated and 7 days after UUO. Scale bars: 50 μm. (**B**) Collagen deposition is augmented in UUO-induced *Irf5^fl/fl^* renal tissues yet diminished in *Irf5^fl/fl^Lyz2*^Cre^ mice. (**C**) Renal *Acta2* and *Vim* mRNA expression in the indicated mouse groups were measured by qPCR. (**D** and **E**) Renal α-SMA and VIM protein expression in the indicated mouse groups were measured by Western blot, the results were subjected to quantitative analysis. Data are expressed as the mean ± SD. Statistical comparisons among multiple groups were performed using 1-way ANOVA followed by Tukey’s post hoc test. *n* = 3–10. *P < 0.05; **P < 0.01; ***P < 0.001; ****P < 0.0001.

**Figure 8:**
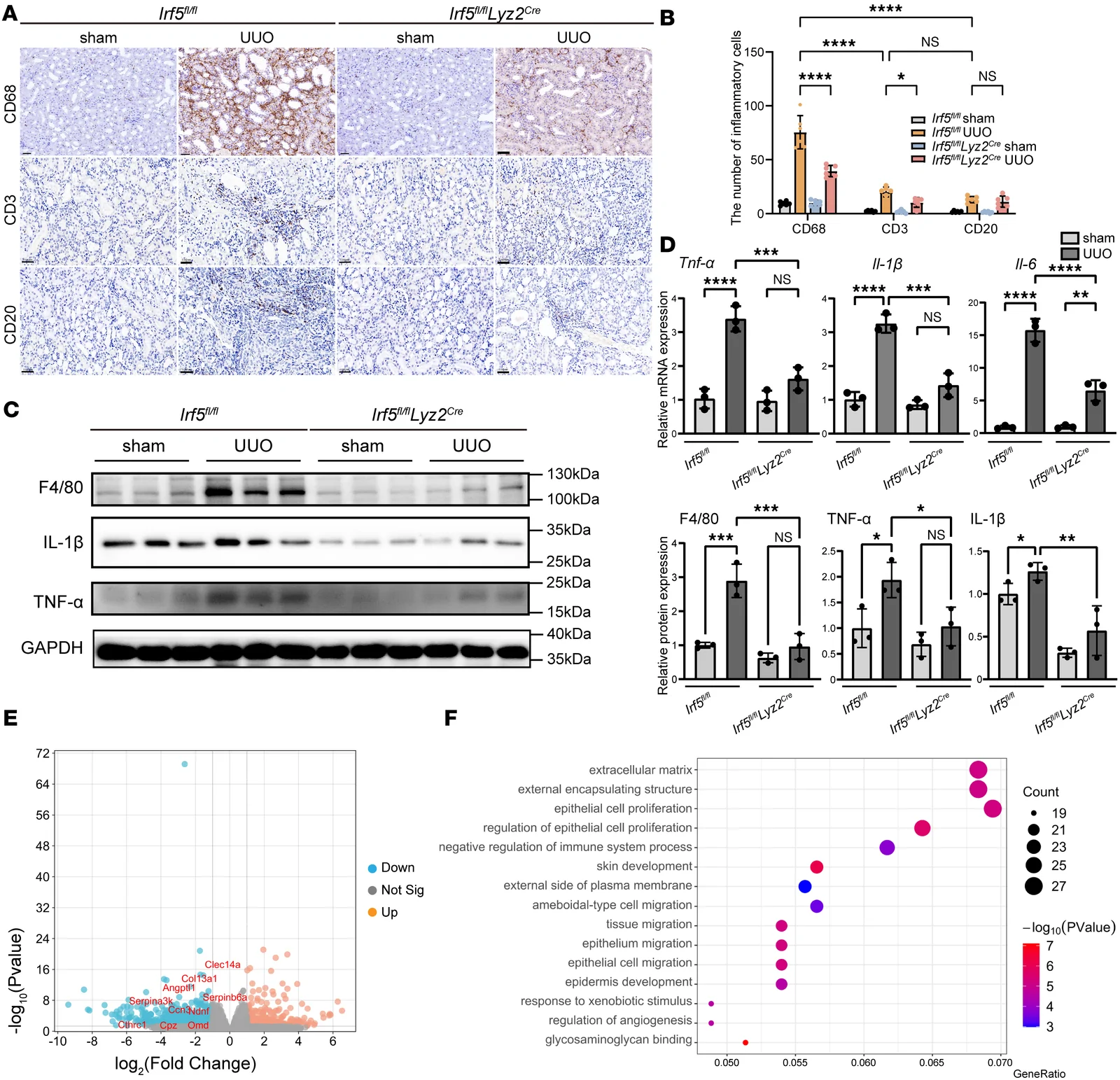
IRF5 deficiency in myeloid cells reduces renal inflammation in vivo. (**A**) IHC images of CD68 (macrophage marker), CD20 (B lymphocyte marker), and CD3 (T lymphocyte marker) of *Irf5^fl/fl^* mice and *Irf5^fl/fl^Lyz2*^Cre^ mice in sham-operated and 7 days after UUO. Scale bars: 50 μm. (**B**) CD68^+^ macrophage numbers were markedly elevated in *Irf5^fl/fl^* UUO tissues yet significantly reduced in *Irf5^fl/fl^Lyz2*^Cre^ UUO renal tissues (*P* < 0.05). (**C** and **D**) Renal protein expression of F4/80, IL-1β, TNF-α in the indicated mouse groups were measured by Western blot. Renal mRNA expression of *Tnf-α*, *Il-1β*, and *Il-6* in the indicated mouse groups were measured by qPCR. (**E**) Gene expression difference of *Irf5^fl/fl^* UUO and *Irf5^fl/fl^Lyz2*^Cre^ UUO mice. (**F**) GO enrichment analysis of *Irf5^fl/fl^* UUO and *Irf5^fl/fl^Lyz2*^Cre^ UUO mice. The highly enriched categories include the extracellular matrix (ECM), external encapsulating structure, and epithelial cell proliferation. Data are expressed as the mean ± SD. Statistical comparisons among multiple groups were performed using 1-way ANOVA followed by Tukey’s post hoc test. *n* = 3–8. *P < 0.05; **P < 0.01; ***P < 0.001; ****P < 0.0001.

**Figure 9:**
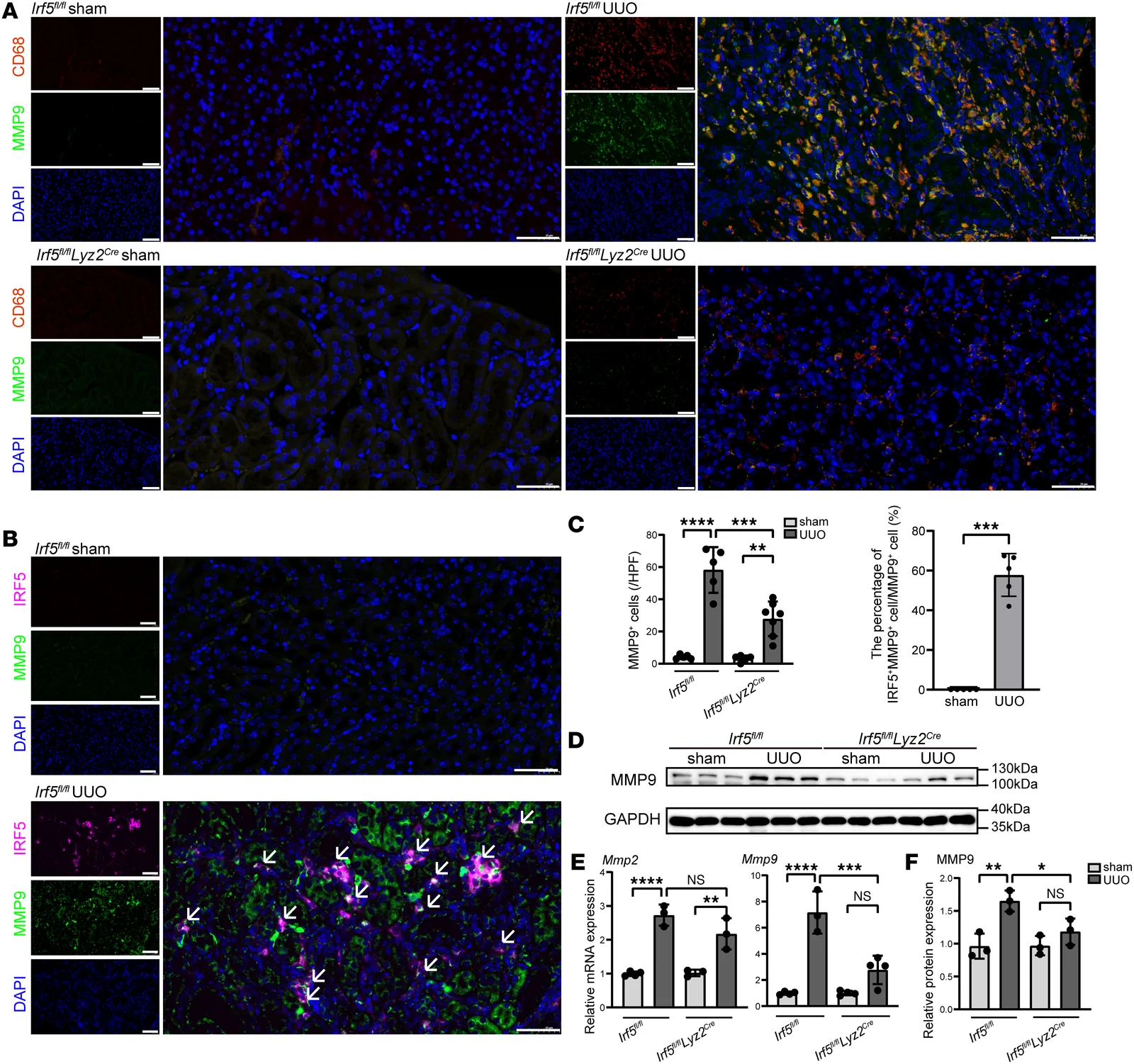
The regulatory effect of IRF5 on MMP9 expression in vivo. (**A**) Representative immunofluorescence images of MMP9 (green) and CD68 (red) expression in renal tissues from sham and UUO groups in *Irf5^fl/fl^* and *Irf5^fl/fl^Lyz2*^Cre^ mice. Scale bars: 50 μm. (**B**) Representative immunofluorescence images of MMP9 (green) and IRF5 (red) expression in renal tissues from sham and UUO groups in *Irf5^fl/fl^* mice. White arrows indicate cells with obvious strong double-positive signals. Scale bars: 50 μm. (**C**) MMP9^+^ cell count in *Irf5^fl/fl^Lyz2*^Cre^ UUO renal tissues was significantly lower than in *Irf5^fl/fl^* UUO group. The proportion of IRF5^+^MMP9^+^ cells within the MMP9^+^ cell was significantly higher in *Irf5^fl/fl^* UUO renal tissues than in sham group. (**D**) Renal MMP9 protein expression in *Irf5^fl/fl^* and *Irf5^fl/fl^Lyz2*^Cre^ were measured by Western blot. MMP9 protein expression was markedly reduced in *Irf5^fl/fl^Lyz2*^Cre^ UUO mice renal tissues versus *Irf5^fl/fl^* UUO controls. (**E**) Renal *Mmp2*, *Mmp9* mRNA expression in *Irf5^fl/fl^* and *Irf5^fl/fl^Lyz2*^Cre^ were measured by qPCR. (**F**) MMP9 protein expression was markedly reduced in *Irf5^fl/fl^Lyz2*^Cre^ UUO mice renal tissues versus *Irf5^fl/fl^* UUO controls. Data are expressed as the mean ± SD. Statistical comparisons among multiple groups were performed using 1-way ANOVA followed by Tukey’s post hoc test. *n* = 3–7. *P < 0.05; **P < 0.01; ***P < 0.001; ****P < 0.0001.

**Figure 10:**
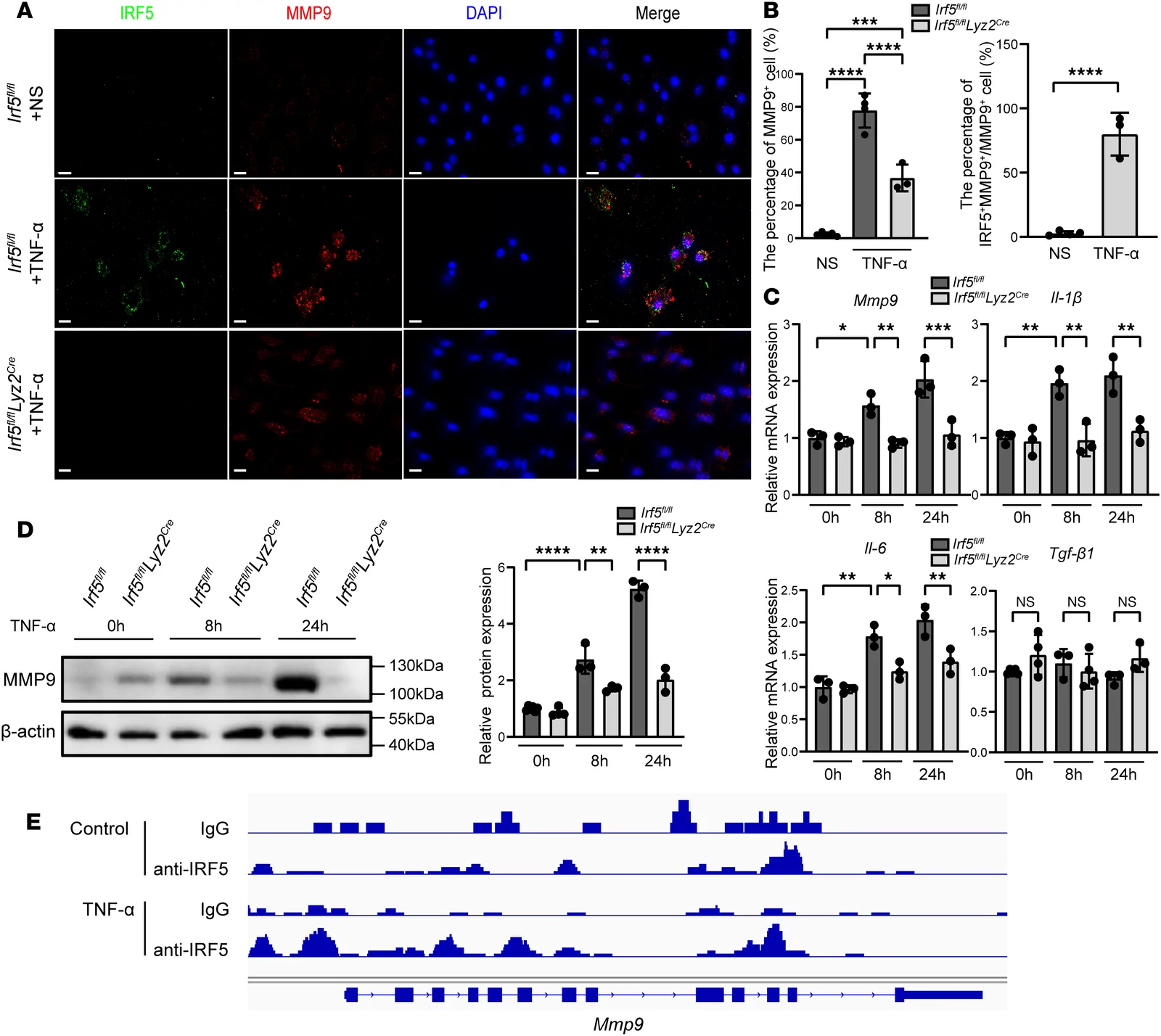
IRF5 positively regulates MMP9 expression and inflammation in BMDMs. (**A**) Representative images of dual immunofluorescence using IRF5 (green), MMP9 (red), and DAPI (blue) in BMDMs. Scale bars: 5 μm. (**B**) MMP9 expression significantly upregulated in BMDMs after TNF-α stimulation, with colocalization within the macrophages. The expression of MMP9 in BMDMs derived from *Irf5^fl/fl^Lyz2*^Cre^ mice was significantly lower than those derived from *Irf5^fl/fl^* mice. (**C** and **D**) *Mmp9* mRNA and MMP9 protein expression in *Irf5^fl/fl^* and *Irf5^fl/fl^Lyz2*^Cre^ BMDMs after 0-, 8-, and 24-hour TNF-α stimulation were measured by qPCR and Western blot. MMP9 protein expression was significantly reduced in *Irf5^fl/fl^Lyz2*^Cre^ BMDMs versus *Irf5^fl/fl^* BMDMs. *Il-1β*, *Il-6*, and *Tgf-β1* mRNA expression in *Irf5^fl/fl^* and *Irf5^fl/fl^Lyz2*^Cre^ BMDMs after 0-, 8-, and 24-hour TNF-α stimulation were measured by qPCR. TNF-α stimulation induced significant upregulation of *Il-1β*, and *IL-6* mRNA in BMDMs, with *Irf5^fl/fl^Lyz2*^Cre^ BMDMs showing a markedly reduced response compared with *Irf5^fl/fl^* BMDMs. (**E**) Representative chromatin landscapes of BMDMs across the *Mmp9* region in the murine genome through CUT&TAG sequencing. Data are expressed as the mean ± SD. Statistical comparisons among multiple groups were performed using 1-way ANOVA followed by Tukey’s post hoc test. *n* = 3–5. *P < 0.05; **P < 0.01; ***P < 0.001; ****P < 0.0001. BMDMs, bone marrow–derived macrophages.

**Figure 11:**
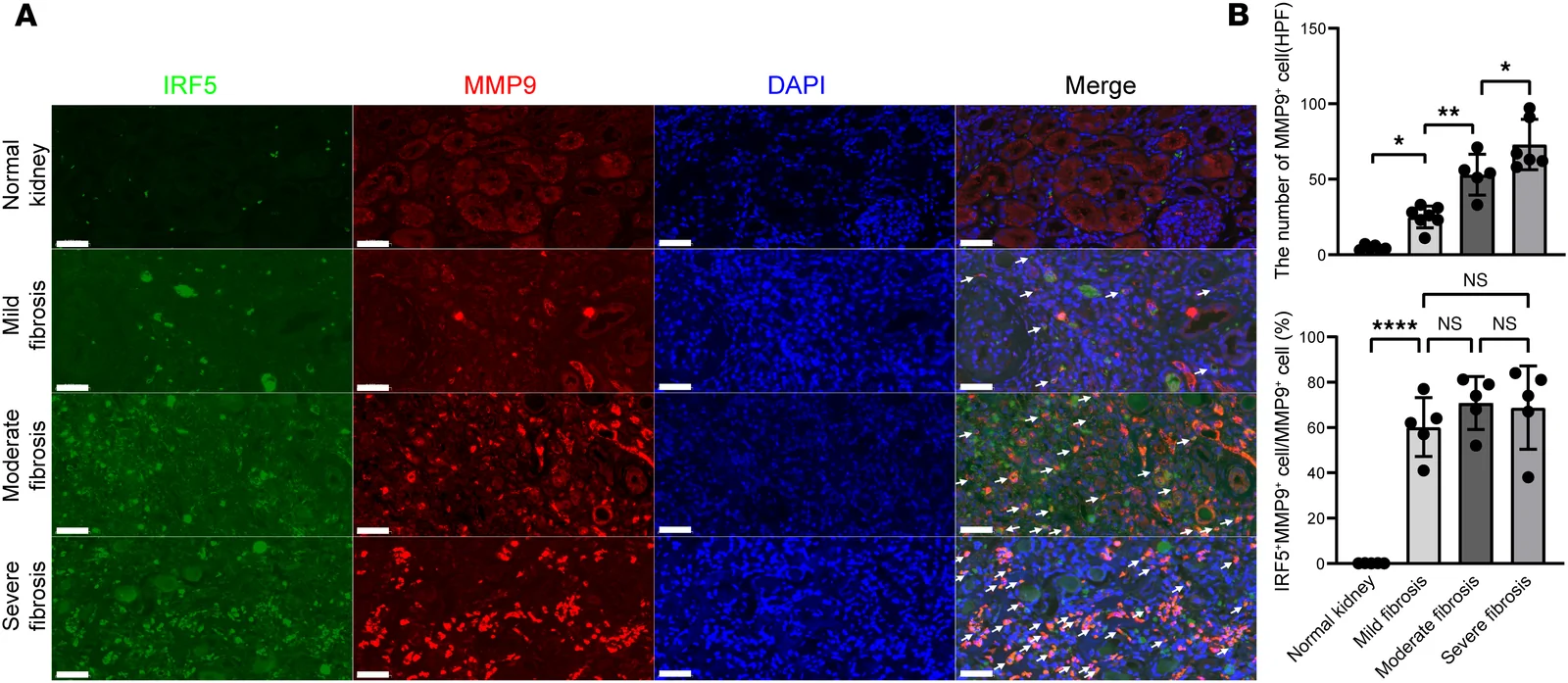
MMP9 expression was significantly elevated and colacalized with IRF5 in patients with obstructive nephropathy. (**A**) Representative images of dual immunofluorescence using IRF5 (green), MMP9 (red), and DAPI (blue) with varying degrees of fibrosis. White arrows indicate cells with obvious strong double-positive signals. Scale bars: 50 μm. (**B**) The number of MMP9^+^ cells increased in fibrotic tissues. IRF5^+^MMP9^+^ cells proportion was significantly greater among MMP9^+^ cells in obstructive nephropathy renal tissues than in controls. Data are expressed as the mean ± SD. Statistical comparisons among multiple groups were performed using 1-way ANOVA followed by Tukey’s post hoc test. *n* = 5–7. *P < 0.05; **P < 0.01; ****P < 0.0001.

**Figure 12:**
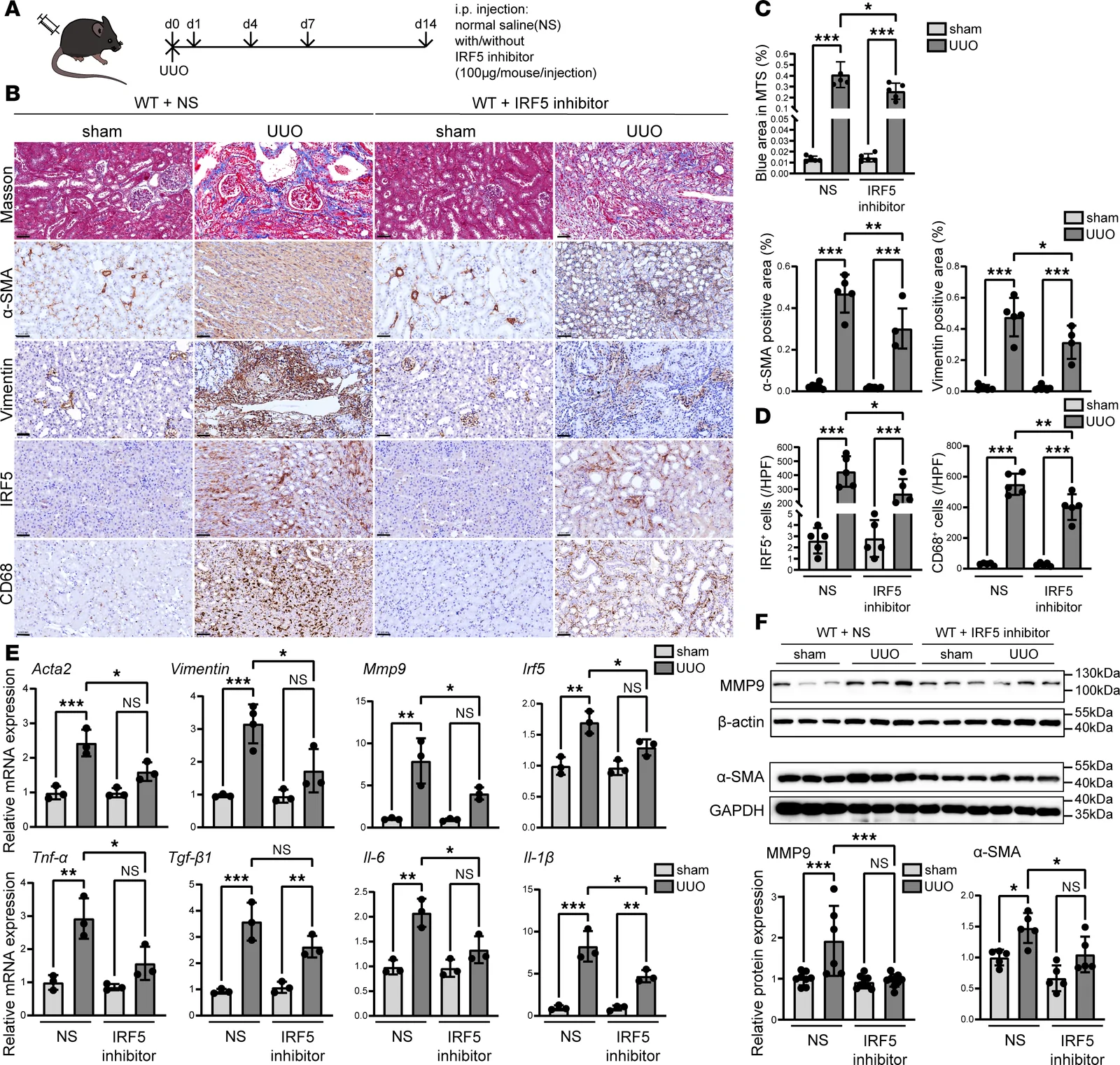
IRF5 inhibitors mitigated renal fibrosis in vivo. (**A**) Schematic graph to show the time point of normal saline or IRF5 inhibitor injection. The drug was administered after UUO on days 0, 1, 4, 7, and 14. (**B**) Representative Masson staining and immunohistochemical images of α-SMA, VIM, IRF5, and CD68 of kidney from mice receiving saline or IRF5 inhibitor treatment. Scale bars: 50 μm. (**C**) Collagen deposition is augmented in UUO-induced WT renal tissues yet diminished in IRF5 inhibitor-treated mice. α-SMA^+^ and VIM^+^ region was significantly upregulated in the UUO group and downregulated in the inhibitor-treated group. (**D**) The numbers of IRF5^+^ cells and CD68^+^ cells were significantly decreased in the obstructed kidneys from the treatment group. (**E**) Renal *Acta2*, *Vim*, *Mmp9*, *Irf5*, *Tnf-α*, *Tgf-β1*, *Il-6*, and *Il-1β* mRNA expression in the indicated mouse groups were measured by qPCR. (**F**) Renal MMP9 and α-SMA protein expression in the indicated mouse groups were measured by Western blot, the results were subjected to quantitative analysis. Data are expressed as the mean ± SD. Statistical comparisons among multiple groups were performed using 1-way ANOVA followed by Tukey’s post hoc test. *n* = 3–9. *P < 0.05; **P < 0.01; ***P < 0.001.

## Version 1 08/06/2026

In-Press Preview

## Version 2 10/08/2026

Electronic publication
