# Supplementary material for: Interferon regulatory factor 5 in myeloid cells promotes obstructive nephropathy-induced renal fibrosis in vivo
Source: JCI Insight. 2026 Aug 6;11(19):e201877. doi: 10.1172/jci.insight.201877 (PMC13726410; doi:10.1172/jci.insight.201877)
Supplement: Supplementary file 1 [file jciinsight-11-201877-s309.pdf]

List of supplemental data files:

Supplemental Figure 1. IRF5 expression is markedly elevated in UUO mouse renal tissues. (A)Gross anatomy of kidney in sham and UUO. (B)The renal parenchyma in the 7d after UUO group was significantly thinner compared to the sham-operated group. (C)Representative H&E and Masson staining images in sham-operated and 7d after UUO. Bars = 50μm. (D)Significant deposition of blue-stained, coarse collagen fibers and marked inflammatory cell infiltration were observed in the obstructed renal tissue.
(E)Immunohistochemical images of α-SMA, Vimentin, and IRF5 in sham and UUO renal tissues. Typical images and quantification are included. Bars = 50 μm. (F)The α-SMA and Vimentin positive area in 7d after UUO was significantly higher than that in sham-operated. The number of IRF5 positive cells in the UUO group was significantly higher compared to sham-operated. (G)Renal Vimentin, Acta2, and Irf5 mRNA expression in the indicated mouse groups were measured by reverse transcription quantitative polymerase chain reaction (RT-qPCR). Data are expressed as the mean ± SD. Comparisons between two groups were performed using an unpaired two-tailed Student's t-test. N = 3-8. *P < 0.05; **P < 0.01; ***P < 0.001; ****P < 0.0001.

Supplemental Figure 2. Validation of IRF5 expression in the indicated groups were measured by Western blot. (A)Renal IRF5 protein expression in the WT mice and global Irf5 deletion mice were measured by Western blot. (B)Renal IRF5 protein expression in the Irf5fl/fl mice and myeloid-specific Irf5 deletion mice were measured by Western blot.(C)Cells IRF5 protein expression in the BMDMs from Irf5fl/fl mice and myeloid-specific Irf5 deletion mice were measured by Western blot.(D)Renal IRF5 protein expression in the control groups and IRF5 inhibitor treatment groups were measured by Western blot. Data are expressed as the mean± SD. Statistical comparisons among multiple groups were performed using one-way ANOVA followed by Tukey's post hoc test. N = 3. *P < 0.05; **P < 0.01; ***P < 0.001.

Supplemental Figure 3. Specificity validation of IRF5 staining. (A) Immunofluorescence images of anti-IRF5 antibodies ab181553 (abcam, Red) and ab21689 (abcam, Green) on kidney sections from WT and Irf5-/- mice after UUO. (B) Immunofluorescence images of anti-IRF5 antibodies ab181553 (abcam, Red) and ab21689 (abcam, Green) on kidney sections from patient with kidney fibrosis. Bars = 50 μm.

Supplemental Figure 4. Specificity validation of MMP9 staining. (A) Immunofluorescence images of anti-MMP9 antibodies 10375-2-AP (proteintech, Red) and MA5-15886 (Invitrogen, Green) on kidney sections from WT and Irf5-/- mice after UUO. Bars = 50 μm.

Supplemental Certificate 1.

Supplemental Certificate 2.

Supplemental Certificate 3.

Supplemental Certificate 4.

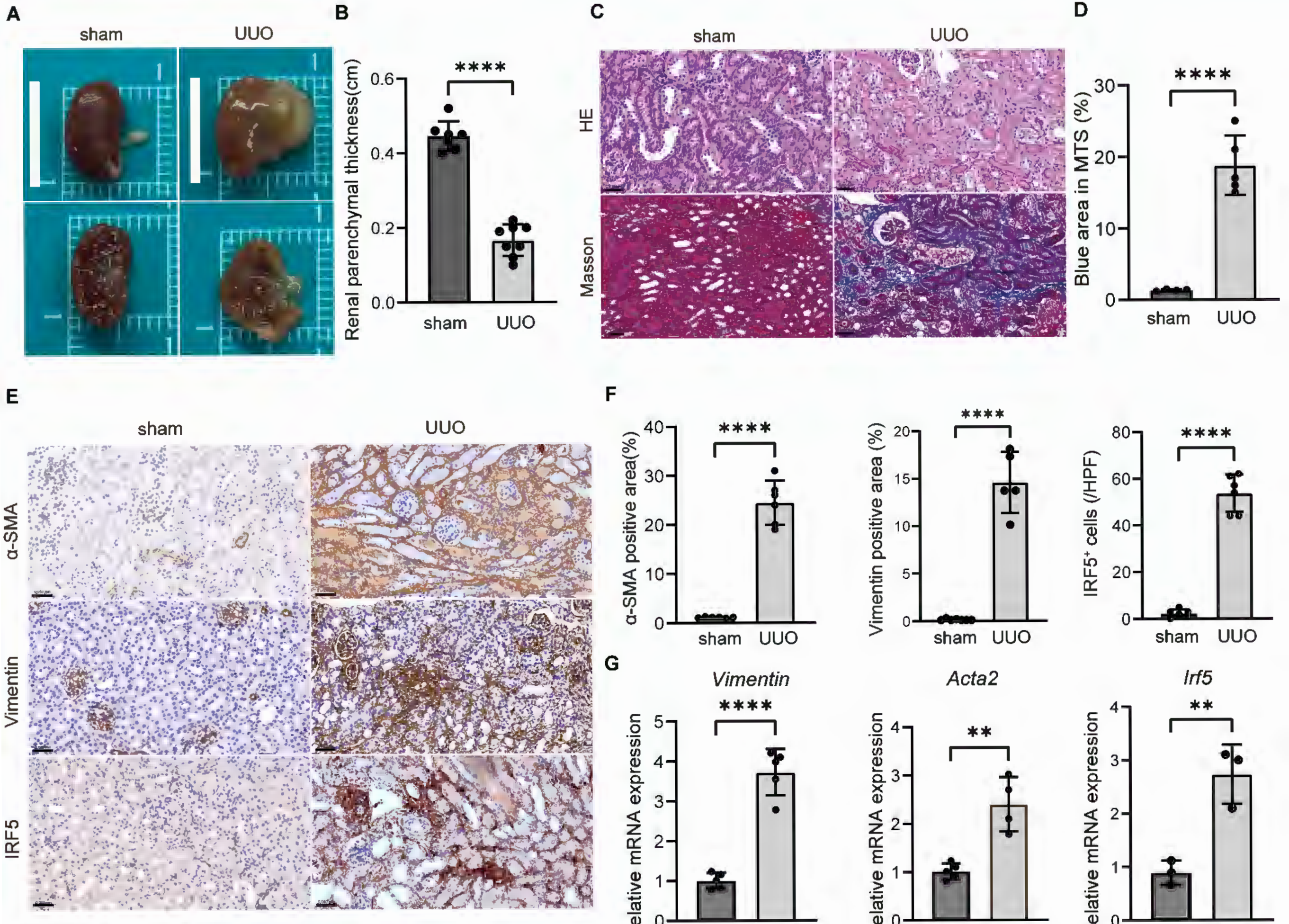

Supplemental figure 1.

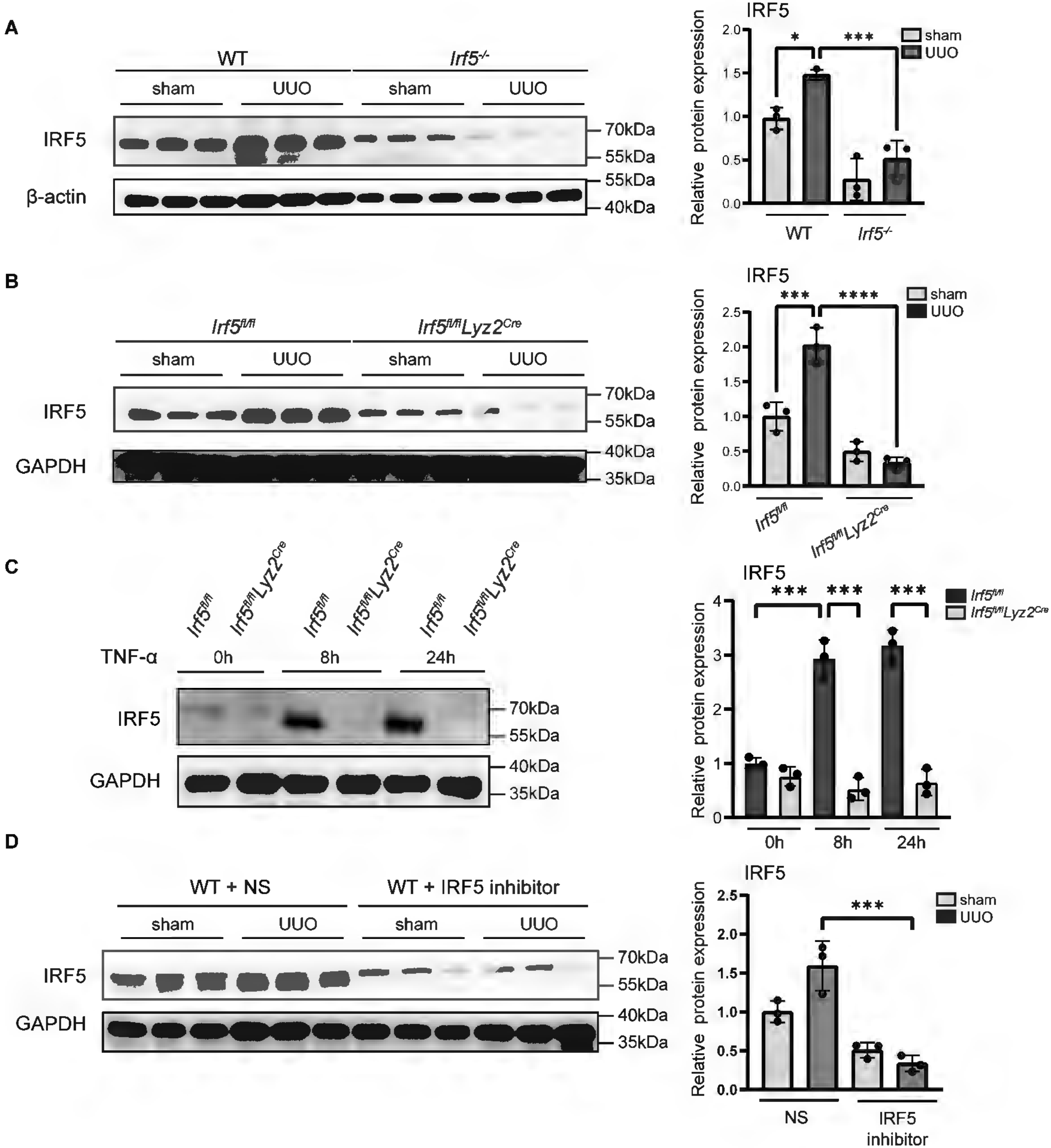

Supplemental figure 2.

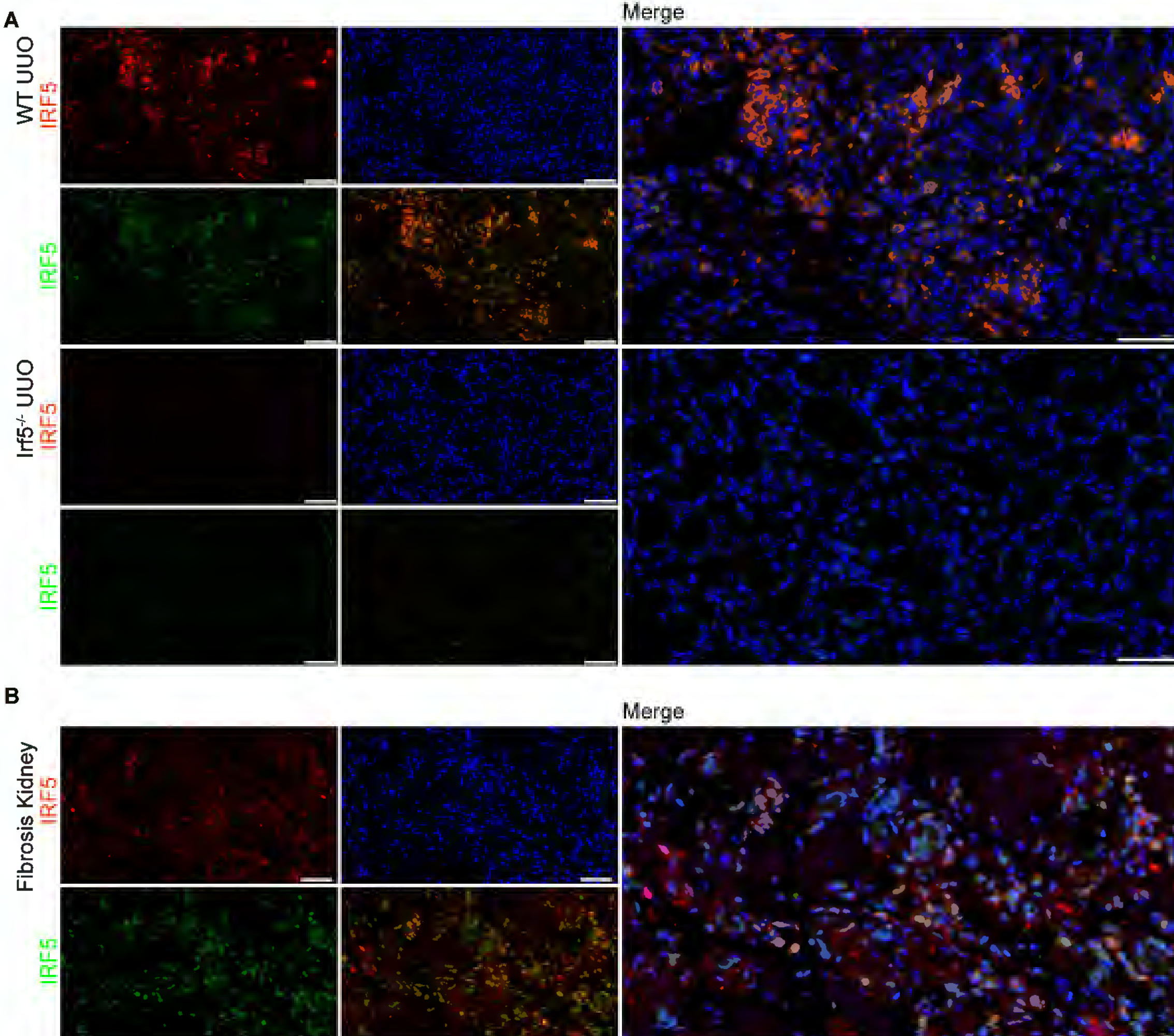

Supplemental figure 3.

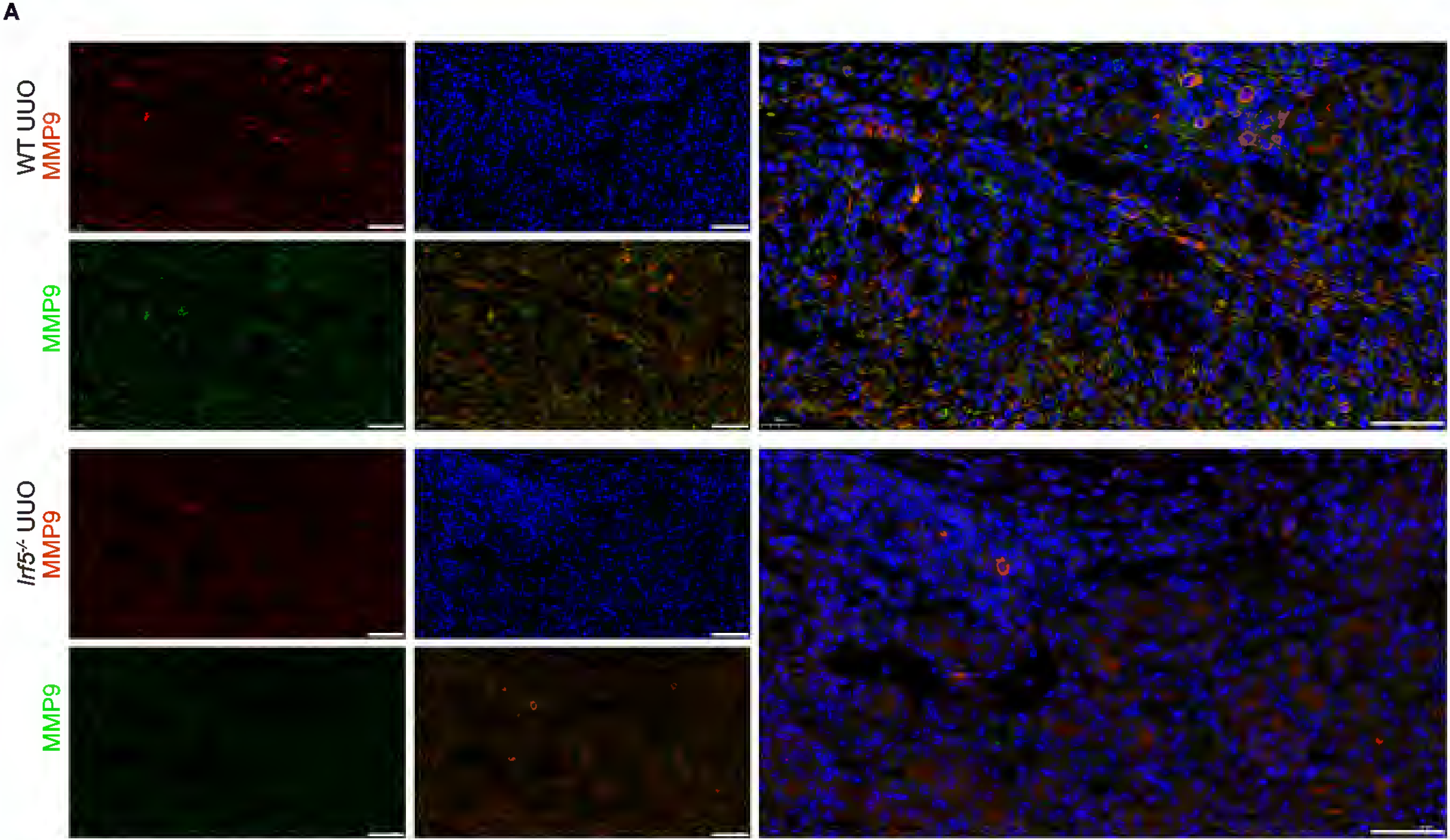

Supplemental figure 4.

Supplemental Table 1. Background information on the kidney fibrosis patients and normal controls

Kidney fibrosis patients (n=30)

			Number(n)	Percentage(%)
Age (years/Mean ± SD)	<18	3.0 ± 1.2	4	13.3%
	≥18	60.2 ± 8.7	26	86.7%
Gender	Male		17	56.7%
	Female		13	43.3%
Reason of obstruction	UPJO*		4	13.3%
	Nephrolith		20	66.7%
	Tumor		6	20%
Degree of inflammatory infiltration	Mild		8	26.7%
	Moderate		9	30%
	Severe		13	43.3%
Degree of kidney fibrosis	Mild		6	20%
	Moderate		10	33.3%
	Severe		14	46.7%

*UPJO: Ureteropelvic Junction Obstruction

Normal controls (n=12)

			Number(n)	Percentage(%)
Age (years/Mean ± SD)	<18	2.8 ± 1.4	4	33.3%
	≥18	52.1 ± 16.3	8	66.7%
Gender	Male		5	41.7%
	Female		7	58.3%

Supplemental Table S9. The list of antibodies used for immunohistochemistry (IHC), immunofluorescence (IF) and western blot (WB)

Antigen	Company		Host	Reactivity	Dilution
IRF5	abcam	ab181553	Rabbit	Mouse, Human	IHC 1:500 IF 1:1000 WB 1:1000
IRF5	abcam	ab21689	Rabbit	Mouse, Human	IF 1:2000 WB 1:5000
IRF5	proteintech	3A4A10	Mouse	Mouse, Huamn	IF 1:500
α-SMA	eBioscience	ab2572996	Mouse	Mouse, Human	IHC 1:500 IF 1:250 WB 1:100
Vimentin	abcam	ab92547	Rabbit	Mouse, Human	IHC 1:500 IF 1:500 WB 1:1000
CD68	abcam	ab283654	Rabbit	Mouse	IHC 1:100 IF 1:50
CD20	abcam	ab64088	Rabbit	Mouse, Human	IHC 1:100 IF 1:100
CD3	abcam	ab16669	Rabbit	Mouse, Human	IHC 1:500 IF 1:200
MMP9	proteintech	10375-2-AP	Rabbit	Mouse, Human	IHC 1:400 IF 1:200 WB 1:1000
MMP9	Invitrogen	MA5-15886	Mouse	Mouse, Human	IF 1:1000
GAPDH	Invitrogen	ab10977387	Mouse	Mouse	WB 1:5000
β-actin	Invitrogen	ab2745831	Rabbit	Mouse	WB 1:10000
F4/80	proteintech	28463-1-AP	Rabbit	Mouse	WB 1:3000
TNF-α	proteintech	60291-1-Ig	Mouse	Mouse	WB 1:1000

Supplemental Table S10. The list of primers for RT-qPCR

Gene	Direction	Sequence
Actin	Forward	5'-CAG CCT TCC TTC TTG GGT ATG-3'
	Reverse	5'-GGC ATA GAG GTC TTT ACG GAT G-3'
Acta2	Forward	5'-GTC CCA GAC ATC AGG GAG TAA-3'
	Reverse	5'-TCG GAT ACT TCA GCG TCA GGA-3'
Il-1β	Forward	5'-CTG CAG CTG GAG AGT GTG G-3'
	Reverse	5'-GGG GAA CTC TGC AGA CTC AA-3'
Il-6	Forward	5'-GCC TTC TTG GGA CTG ATG CT-3'
	Reverse	5'-GCC ATT GCA CAA CTC TTT TCT CA-3'
Irf5	Forward	5'-AGA GAC AGG GAA GTA CAC TGA AG-3'
	Reverse	5'-TGG AAG TCA CGG CTT TTG TTA AG-3'
Tgfβ1	Forward	5'-CCA CCT GCA AGA CCA TCG AC-3'
	Reverse	5'-CTG GCG AGC CTT AGT TTG GAC-3'
Tnfα	Forward	5'-CAG GCG GTG CCT ATG TCT C-3'
	Reverse	5'-CGA TCA CCC CGA AGT TCA GTA G-3'
Vimentin	Forward	5'-CAC TAG CCG CAG CCT CTA TTC-3'
	Reverse	5'-GTC CAC CGA GTC TTG AAG CA-3'

Supplemental Certificate 1

南京大学-南京生物医药研究院
Nanjing Biomedical Research Institute of Nanjing University

Irf5 基因敲除小鼠项目完成报告

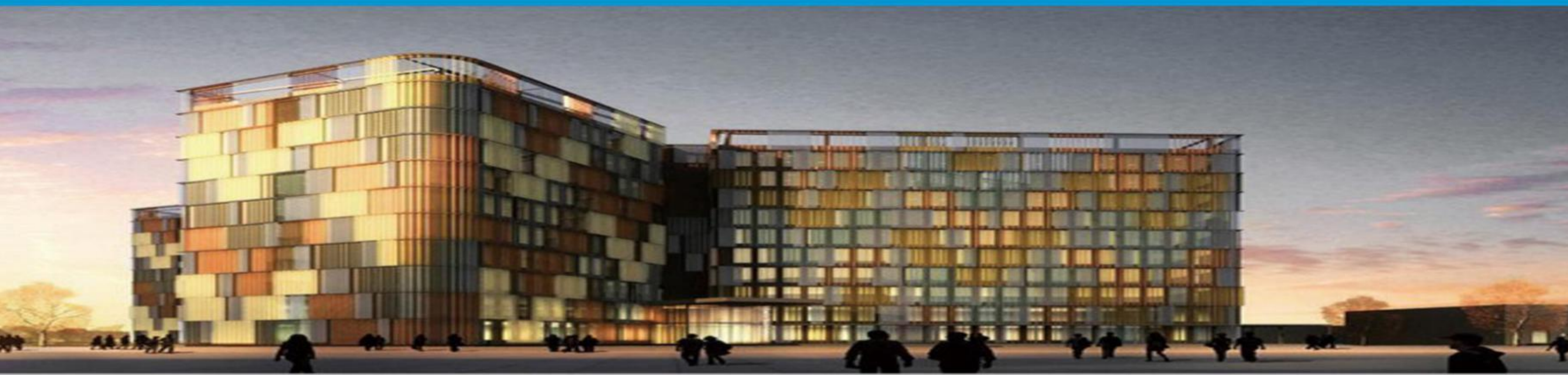

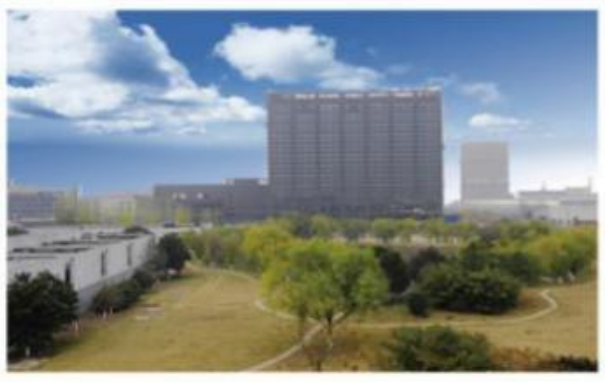

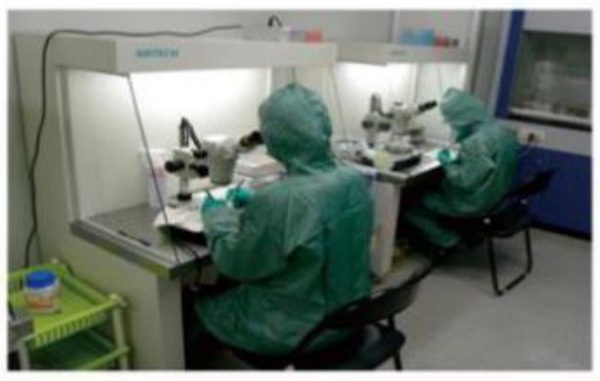

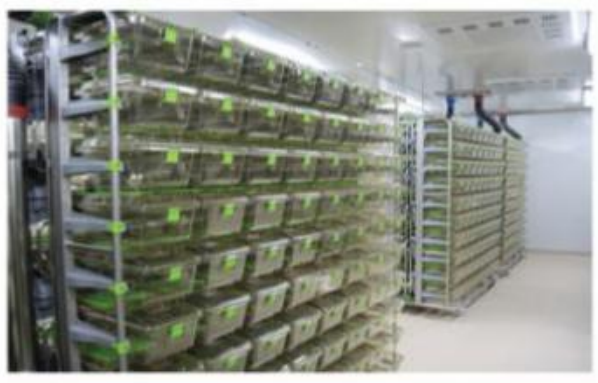

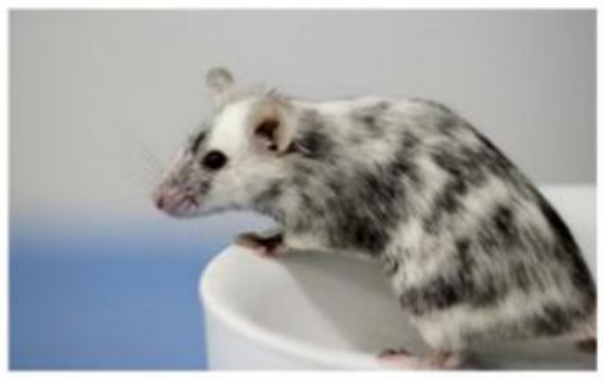

NBRI·技术服务部

Irf5 基因敲除小鼠项目完成报告

项目编号：XM709673

项目类型：Cas9-KO

项目背景：C57BL/6J

客户姓名：蔡哲钧

客户单位：浙江大学

NBRI

目录

一、基因基本信息 3

二、 项目策略 3

三、gRNA 序列信息 4

四、F0 代阳性小鼠信息 4

五、F1 代小鼠鉴定结果与小鼠信息 5

NBRI

一、基因基本信息

敲除基因名称（NCBI 号）：Irf5（27056）

敲除基因 NCBI 网址链接：https://www.ncbi.nlm.nih.gov/gene/27056

敲除基因名称（MGI 号）：Irf5（MGI:1350924）

敲除基因 Ensembl 网址链接：

http://uswest.ensembl.org/Mus_musculus/Gene/Summary?db=core;g=ENSMUSG00000029771;r=6:29526625-29541871

方案针对的转录本（Ensembl 号）：Irf5-201 (ENSMUST00000004392.11)

方案敲除的 exon：Exon2~Exon9

二、项目策略

2.1 项目策略图

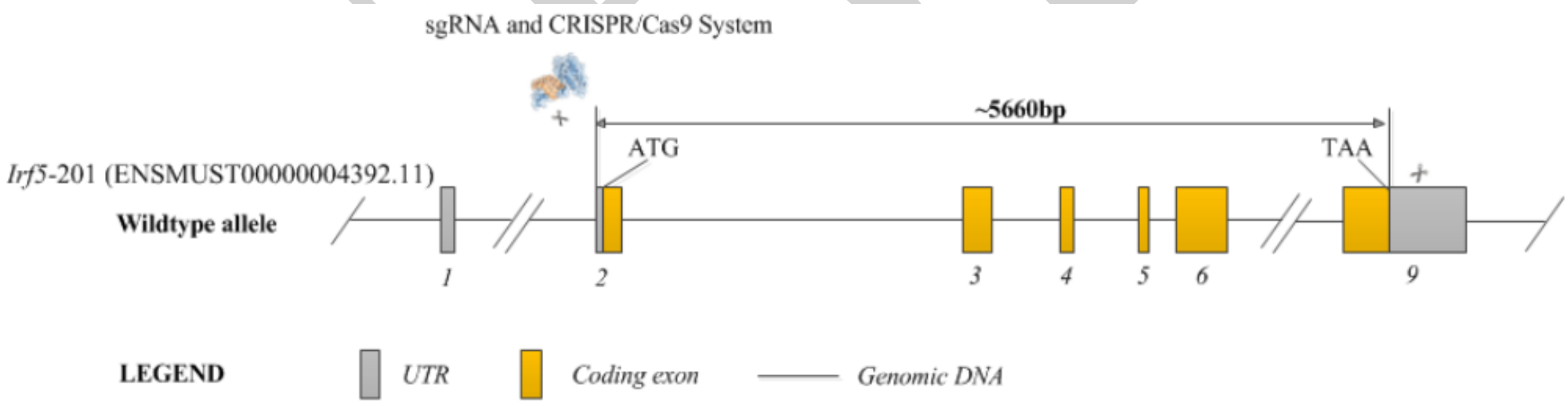

2.2 项目策略概述

利用 CRISPR/Cas9 技术，设计并体外转录 gRNA，将 Cas9、gRNA 同时注射到小鼠的受精卵中。Cas9 蛋白在 gRNA 引导下结合到靶位点进而造成 DNA 双链断裂，从而实现靶位点碱基序列缺失，最终实现及基因敲除。

三、gRNA 序列信息

gRNA 名称	gRNA 序列（5’ →3’ ）	PAM
gRNA1	AAGGAAACTCCCGTGCTAGT	GGG
gRNA2	CTTGGCCCCAGACGCCTAGA	AGG
gRNA3	CGTAGACTGATATAGCAATG	TGG
gRNA4	GGTGATTTCTCTAATATGCC	TGG

四、F0 代阳性小鼠信息

经受精卵显微注射，胚胎移植，获得 F0 小鼠。通过 PCR，测序确认，共获得如下的阳性 F0 代小鼠。

具体信息如下：

ID	Gender	color	Gty	DOB	Gen	Remark
1	♂	B	阳性	2018/5/7	F0	配繁
4	♂	B	阳性	2018/5/7	F0	配繁
9	♀	B	阳性	2018/5/7	F0	配繁
12	♂	B	阳性	2018/5/7	F0	
15	♂	B	阳性	2018/5/7	F0	
17	♀	B	阳性	2018/5/7	F0	
18	♀	B	阳性	2018/5/7	F0	配繁
19	♀	B	阳性	2018/5/7	F0	

NBRI

五、F1 代小鼠鉴定结果与小鼠信息

5.1 鉴定策略示意图

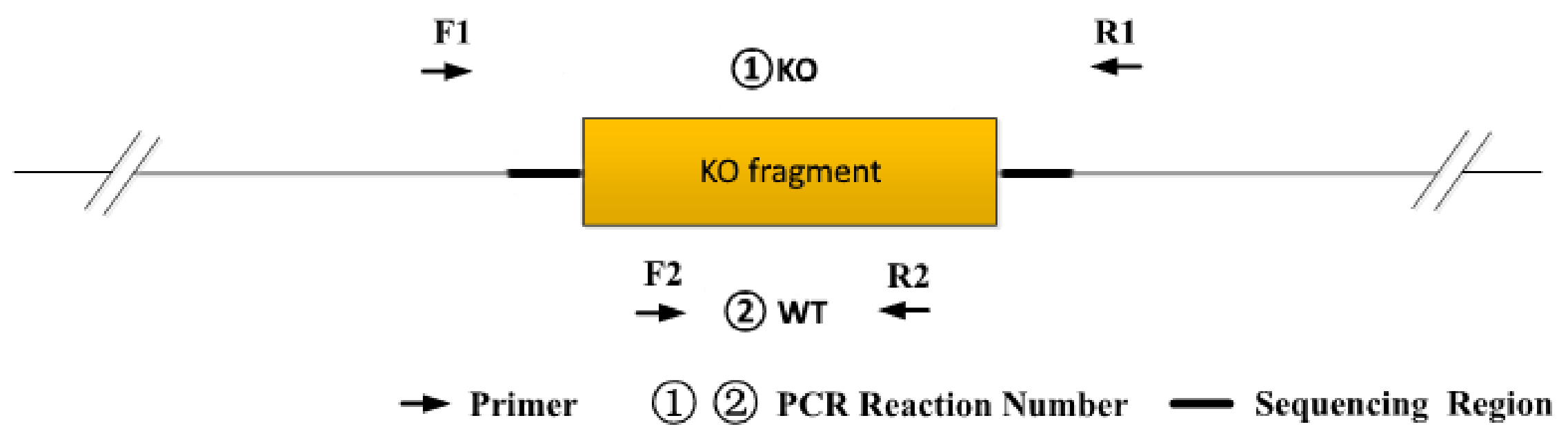

野生型：① PCR 反应未获得 ~748bp 条带；② PCR 反应可以获得 459bp 条带

杂合子：① PCR 反应可以获得~748bp 条带；② PCR 反应可以获得 459bp 条带

纯合子：① PCR 反应可以获得~748bp 条带；② PCR 反应未获得 459bp 条带

5.2 PCR 引物信息

PCR 序号	引物名称	引物详细名称	引物序列	产物大小	备注
①KO	F1	709673-Irf5-ko-tF1	CAGAGTCCTGTGAACGAACCTG	WT: 6816bp<br>Targeted:~748bp	/
	R1	709673-Irf5-ko-tR1	ACAACAGAAGCCACTGGCAC		
②WT	F2	709673-Irf5-wt-tF1	TCTTTGTGCTAGGGATCAAGGC	WT: 459bp<br>Targeted: 0bp	/
	R2	709673-Irf5-wt-tR1	CAGGTTACAGTATGTGTGGGCC		

5.3 F1 小鼠鉴定结果

鉴定结果
根据 PCR 策略示意图，经 PCR 和测序确认，**22，23，28，29** 号小鼠为阳性 F1 代小鼠。<br>具体删除方式如下：

22#，23#：-11bp,+2bp,-5973bp/wt

actcaagaatgggtggcccaca------***11bp***------CCcacgggagttccttctt//gccccagacgc------***5973bp***------ctggctgggaagaaatgaagccagc

28#，29#：-6068bp,+1bp/wt

gcactcaagaatgggtggcccacagca------***6068bp***------Ggcctggctgggaagaaatgaagccagcagcc

电泳图如下：

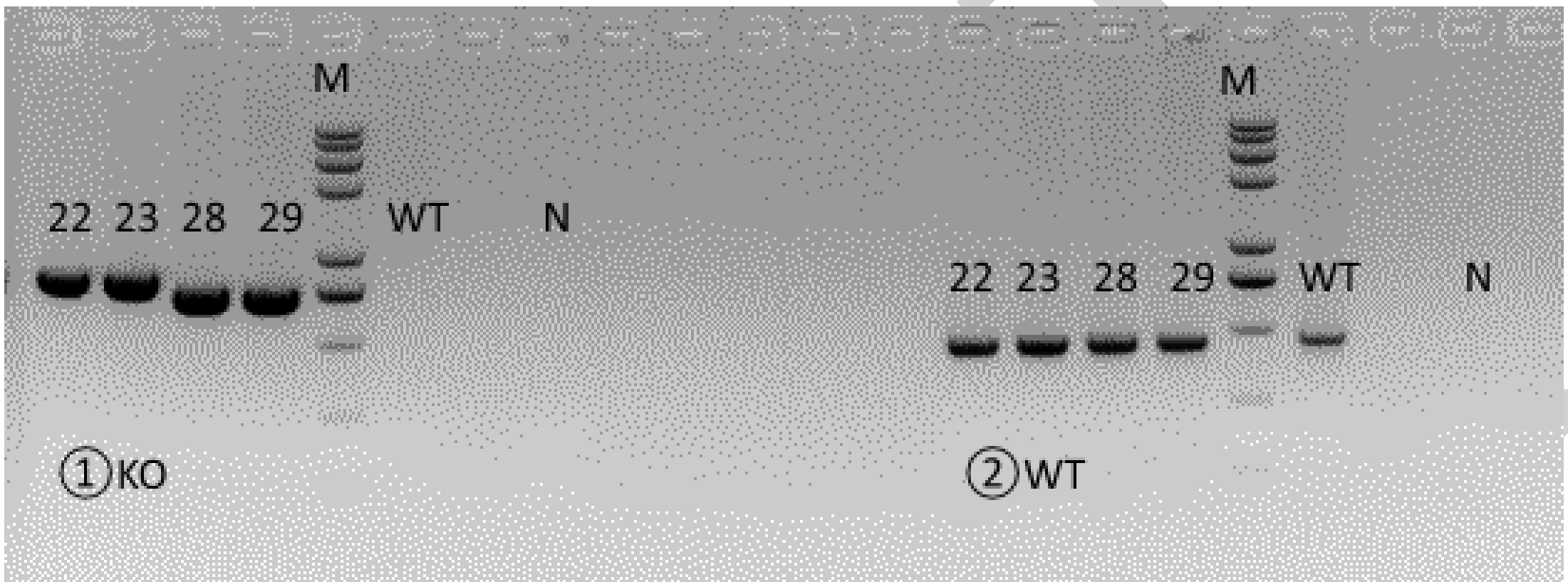

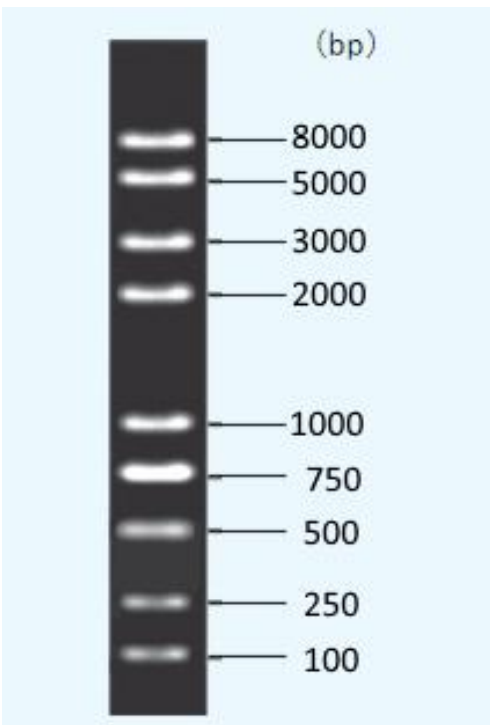

注：数字为鼠尾号，WT 为 C57BL/6J 野生型，N 为 negative 空白对照，M 为 DNA Marker

5.4 PCR 条件：

PCR Reaction Component		
**Seg.**	**reaction component**	**Volume (μl)**
1	2 × Taq Master Mix ，Dye Plus，(Vazyme P112-03)	12.5
2	ddH2O	9.5

3	Primer A(10pmol/μl)	1
4	Primer B(10pmol/μl)	1
5	Template(≈100ng/μl)	1

PCR program

Seg.	Temp.	Time	Cycle
1	95℃	5min	
2	98℃	30s	20×
3	65℃（-0.5℃/cycle）	30s	
4	72℃	45s	
5	98℃	30s	20×
6	55℃	30s	
7	72℃	45s	
8	72℃	5min	
9	10℃	hold	

5.5 阳性 F1 代小鼠信息如下：

ID	Gender	color	Gty	DOB	Gen	F/M
22	♂	B	-11bp,+2bp,-5973bp/wt，编码区完全删除	2018/7/16	F1	4#/C57BL/6J
23	♂	B	-11bp,+2bp,-5973bp/wt，编码区完全删除	2018/7/16	F1	4#/C57BL/6J
28	♂	B	-6068bp,+1bp/wt，编码区完全删除	2018/7/20	F1	C57BL/6J/18#
29	♂	B	-6068bp,+1bp/wt，编码区完全删除	2018/7/20	F1	C57BL/6J/18#

NBRI

撰写人信息：

姓名：沈彦花

单位：南京大学-南京生物医药研究院

地址：江苏省南京市浦口高新区学府路 12 号

电话：025-58641534

Email：shenyh@nbri-nju.com

NBRI 工具小鼠销售平台

如果您需要工具鼠或其他品系小鼠信息，欢迎登录我们的网站（http://www.nbri-nju.com）查询。敬请关注 NBRI 的企业微信，我们将竭诚为您提供最完美的服务！

NBRI

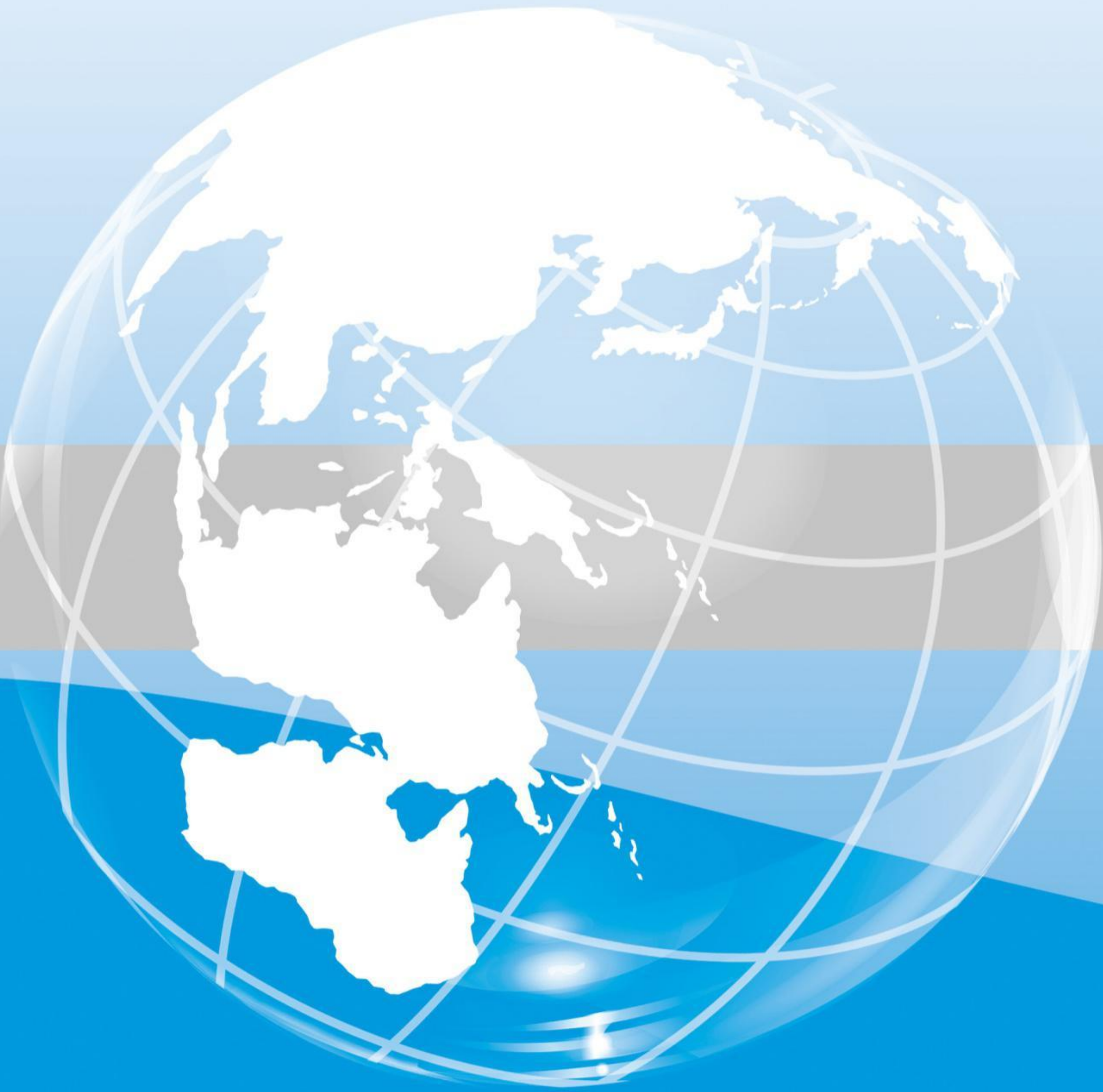

地址：南京市浦口区学府路12号
邮编：210061
电话：025-58641520
传真：025-58641500
网址：www.nbri-nju.com   www.nrcmm.cn
E-mail: services@nbri-nju.com

Supplemental Certificate 2

The Jackson Laboratory GENOTYPING PROTOCOLS DATABASE

B6.129P2-*Lyz2*$^{tm1(cre)Ifo}$/J

Stock No: 004781
Protocol 26499: Separated MCA Assay - Lyz2<tm1(cre)lfo>
Version 5.2

Notes

Use Perfect Match© (Stratagene) added before Taq to delete non-specific bands.

The genotyping protocol(s) presented here have been optimized for reagents and conditions used by The Jackson Laboratory (JAX). To genotype animals, JAX recommends researchers validate the assay independently upon receipt of animals into their facility. Reaction cycling temperature and times may require additional optimization based on the specific genotyping reagents used.

Expected Results

Mutant = ~700 bp
Heterozygote = ~700 bp and ~350 bp
Wild type = 350 bp
Separated by gel electrophoresis on a 1.5% agarose gel.

JAX Protocol

Protocol Primers

PRIMER	5' LABEL	SEQUENCE 5' → 3'	3' LABEL	PRIMER TYPE	REACTION	NOTE
oIMR3066		CCC AGA AAT GCC AGA TTA CG		Mutant	A	
oIMR3067		CTT GGG CTG CCA GAA TTT CTC		Common	A, B	
oIMR3068		TTA CAG TCG GCC AGG CTG AC		Wild type	B	

Reaction A

COMPONENT	FINAL CONCENTRATION
ddH2O	
Kapa 2G HS buffer	1.30 X
MgCl2	2.60 mM
dNTPS-kapa	0.26 mM
oIMR3066	0.50 uM
oIMR3067	0.50 uM
Glycerol	6.50 %
Dye	1.00 X
Kapa 2G HS taq polym	0.03 U/ul
DNA	

Cycling

STEP	TEMP °C	TIME	NOTE
1	94.0	--	
2	94.0	--	
3	65.0	--	-0.5 C per cycle decrease
4	68.0	--	
5		--	repeat steps 2-4 for 10 cycles (Touchdown)
6	94.0	--	
7	60.0	--	
8	72.0	--	
9		--	repeat steps 6-8 for 28 cycles
10	72.0	--	
11	10.0	--	hold

JAX uses a very high speed Taq (~1000 bp/sec), use cycling times recommended for your reagents.

JAX uses a 'touchdown' cycling protocol and therefore has not calculated the optimal annealing temperature for each set of primers.

Reaction B

COMPONENT	FINAL CONCENTRATION
ddH2O	
Kapa 2G HS buffer	1.30 X
MgCl2	2.60 mM
dNTPS-kapa	0.26 mM
oIMR3067	0.50 uM
oIMR3068	0.50 uM
Glycerol	6.50 %
Dye	1.00 X
Kapa 2G HS taq polym	0.03 U/ul
DNA	

Cycling

STEP	TEMP °C	TIME	NOTE
1	94.0	--	
2	94.0	--	
3	65.0	--	-0.5 C per cycle decrease
4	68.0	--	
5		--	repeat steps 2-4 for 10 cycles (Touchdown)
6	94.0	--	
7	60.0	--	
8	72.0	--	
9		--	repeat steps 6-8 for 28 cycles
10	72.0	--	
11	10.0	--	hold

JAX uses a very high speed Taq (~1000 bp/sec), use cycling times recommended for your reagents.

JAX uses a 'touchdown' cycling protocol and therefore has not calculated the optimal annealing temperature for each set of primers.

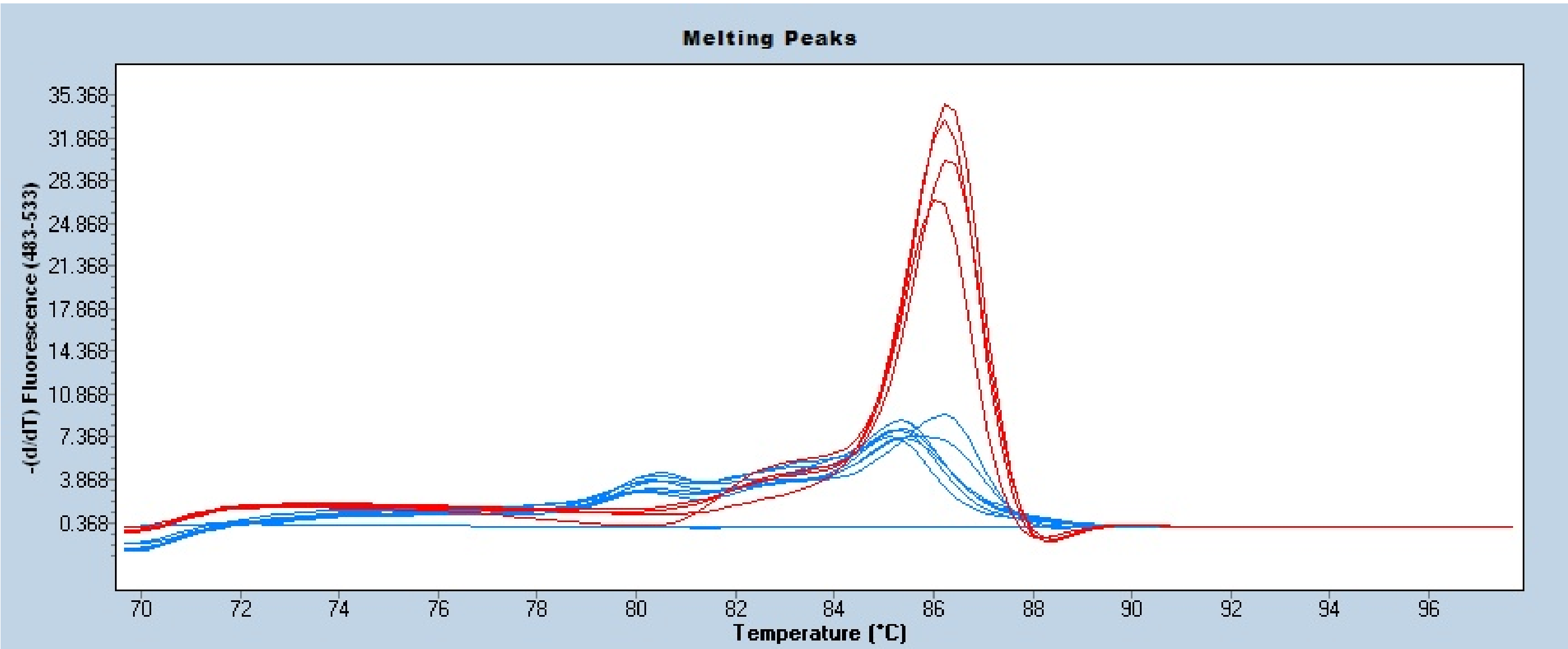

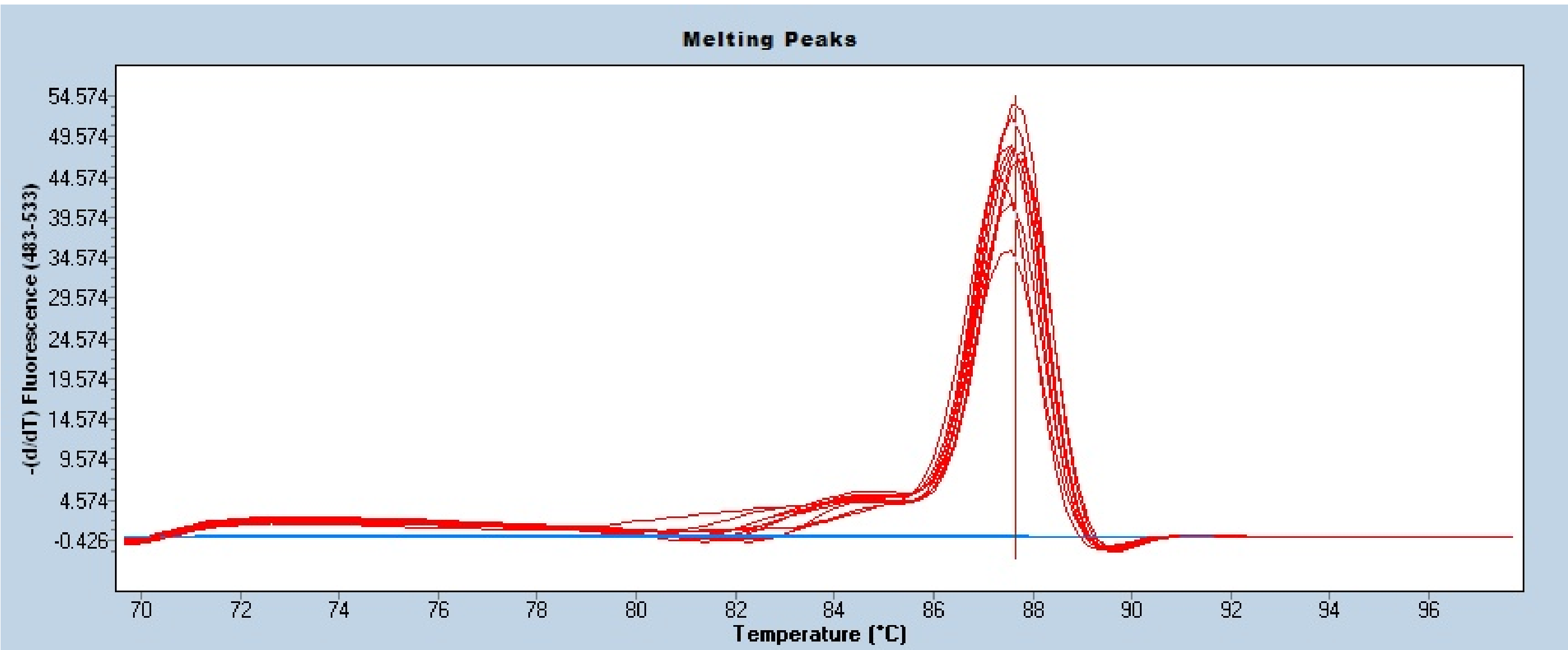

C57BL/6-$Irf5^{tm1Ppr}$/J

Stock No: 017311
Protocol 36936: Separated PCR Assay - Irf5<tm1Ppr>-Cre recombined
Version 1.0

Notes

The genotyping protocol(s) presented here have been optimized for reagents and conditions used by The Jackson Laboratory (JAX). To genotype animals, JAX recommends researchers validate the assay independently upon receipt of animals into their facility. Reaction cycling temperature and times may require additional optimization based on the specific genotyping reagents used.

Expected Results

Mutant = ~300 bp
Heterozygote = 196 bp and ~300 bp
Wild type = 196 bp

JAX Protocol

Protocol Primers

PRIMER	5' LABEL	SEQUENCE 5' → 3'	3' LABEL	PRIMER TYPE	REACTION	NOTE
12932		AGG GCC TGT CCA GAA TTA GG		Mutant Reverse	B	
50085		CTT TGC CAT GAA CCA CTC AG		Wild type Forward	A	
50086		GAT GGT GTT GTC CCC ATC CT		Wild type Reverse	A	
50087		CCA CAG CAC CCA CTA GCA C		Mutant Forward	B	

Reaction A

COMPONENT	FINAL CONCENTRATION
ddH2O	
Kapa 2G HS buffer	1.30 X
MgCl2	2.60 mM
dNTPS-kapa	0.26 mM
50085	0.50 uM
50086	0.50 uM
Glycerol	6.50 %
Dye	1.00 X
Kapa 2G HS taq polym	0.03 U/ul
DNA	

Cycling

STEP	TEMP °C	TIME	NOTE
1	94.0	--	
2	94.0	--	
3	65.0	--	-0.5 C per cycle decrease
4	68.0	--	
5		--	repeat steps 2-4 for 10 cycles (Touchdown)
6	94.0	--	
7	60.0	--	
8	72.0	--	
9		--	repeat steps 6-8 for 28 cycles
10	72.0	--	
11	10.0	--	hold

JAX uses a very high speed Taq (~1000 bp/sec), use cycling times recommended for your reagents.

JAX uses a 'touchdown' cycling protocol and therefore has not calculated the optimal annealing temperature for each set of primers.

Reaction B

COMPONENT	FINAL CONCENTRATION
ddH2O	
Kapa 2G HS buffer	1.30 X
MgCl2	2.60 mM
dNTPS-kapa	0.26 mM
12932	0.50 uM
50087	0.50 uM
Glycerol	6.50 %
Dye	1.00 X
Kapa 2G HS taq polym	0.03 U/ul
DNA	

Cycling

STEP	TEMP °C	TIME	NOTE
1	94.0	--	
2	94.0	--	
3	65.0	--	-0.5 C per cycle decrease
4	68.0	--	
5		--	repeat steps 2-4 for 10 cycles (Touchdown)
6	94.0	--	
7	60.0	--	
8	72.0	--	
9		--	repeat steps 6-8 for 28 cycles
10	72.0	--	
11	10.0	--	hold

JAX uses a very high speed Taq (~1000 bp/sec), use cycling times recommended for your reagents.

JAX uses a 'touchdown' cycling protocol and therefore has not calculated the optimal annealing temperature for each set of primers.

JR 409613 Irf5tm1Ppr-Cre recombined WT

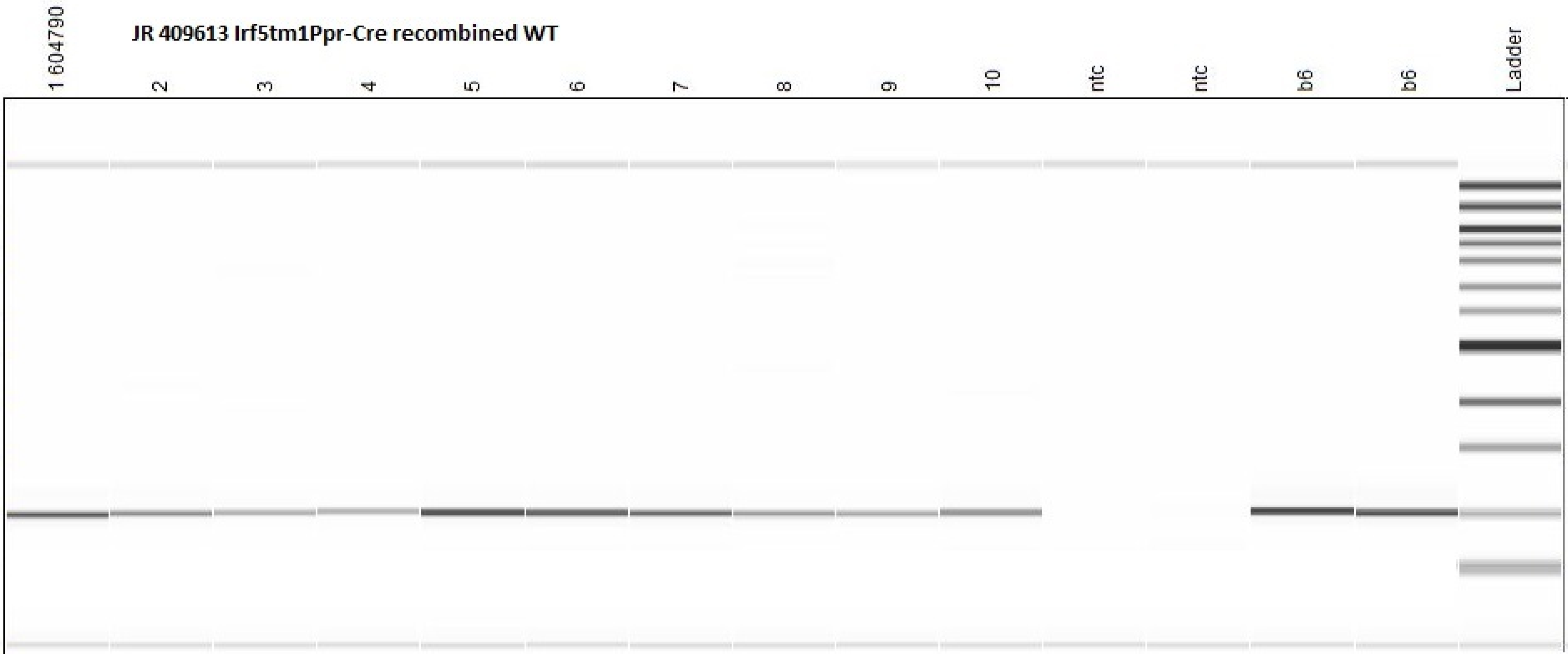

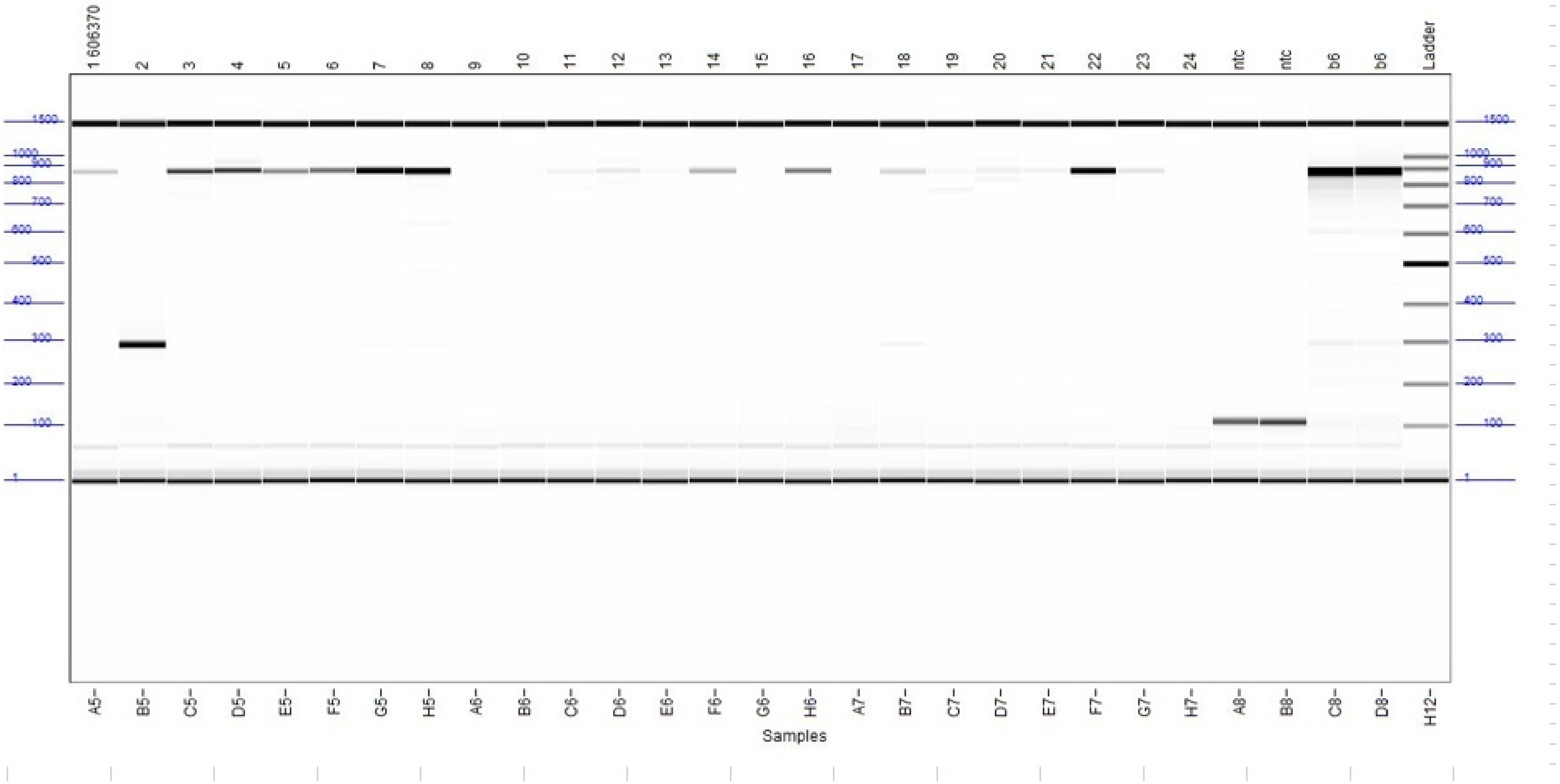

1 606370
2
3
4
5
6
7
8
9
10
11
12
13
14
15
16
17
18
19
20
21
22
23
24
ntc
ntc
b6
b6
Ladder
1500
1000
900
800
700
600
500
400
300
200
100
1
A5
B5
C5
D5
E5
F5
G5
H5
A6
B6
C6
D6
E6
F6
G6
H6
A7
B7
C7
D7
E7
F7
G7
H7
A8
B8
C8
D8
H12
Samples

Supplemental Certificate 4

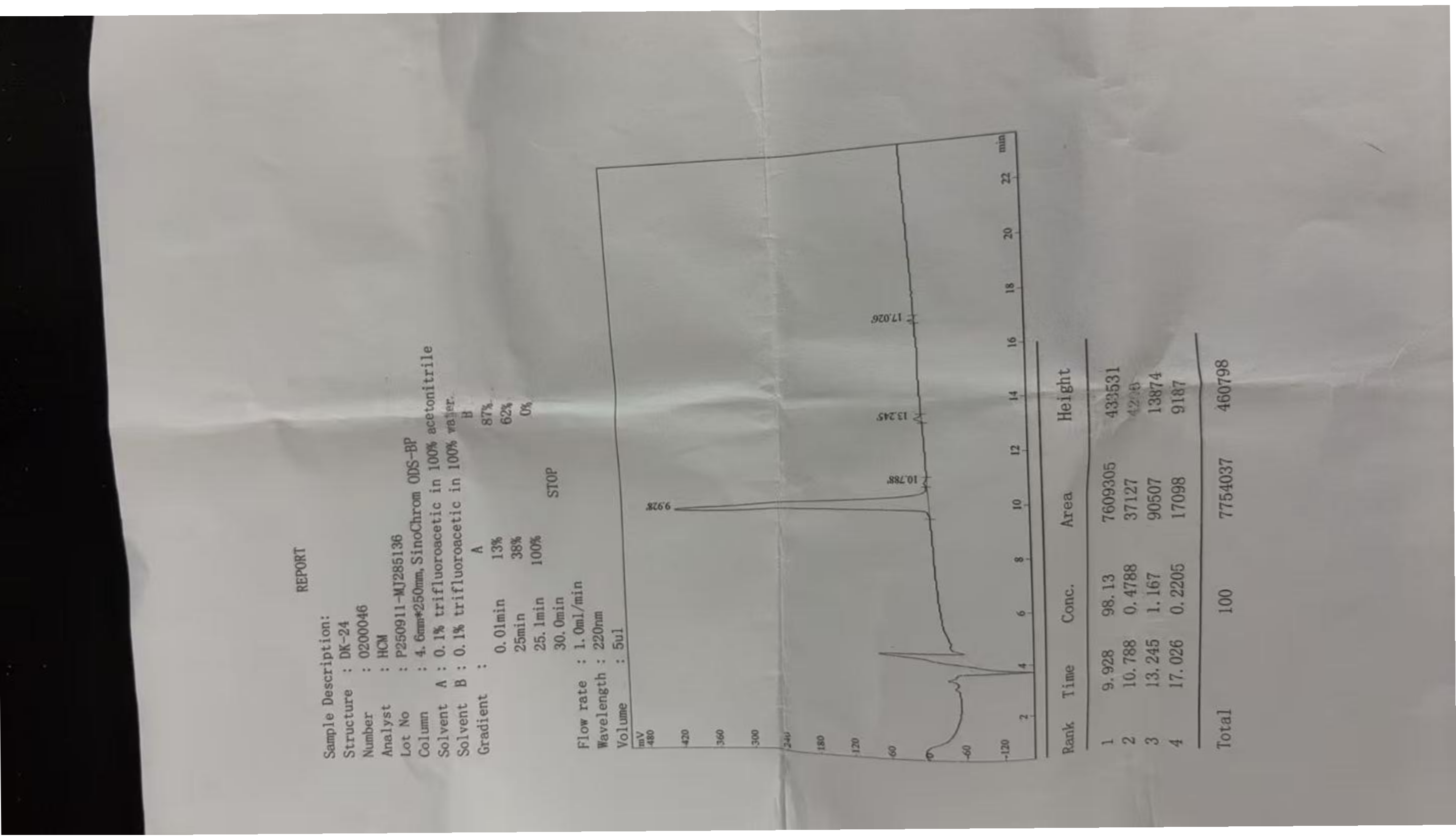

REPORT

Sample Description:
Structure : DK-24
Number : 0200046
Analyst : HCM
Lot No : P250911-MJ285136
Column : 4.6mm*250mm, SinoChrom ODS-BP
Solvent A : 0.1% trifluoroacetic in 100% acetonitrile
Solvent B : 0.1% trifluoroacetic in 100% water
Gradient :

	A	B
0.01min	13%	87%
25min	38%	62%
25.1min	100%	0%
30.0min	STOP	

Flow rate : 1.0ml/min
Wavelength : 220nm
Volume : 5ul

Rank	Time	Conc.	Area	Height
1	9.928	98.13	7609305	433531
2	10.788	0.4788	37127	[illegible]
3	13.245	1.167	90507	13874
4	17.026	0.2205	17098	9187
Total		100	7754037	460798

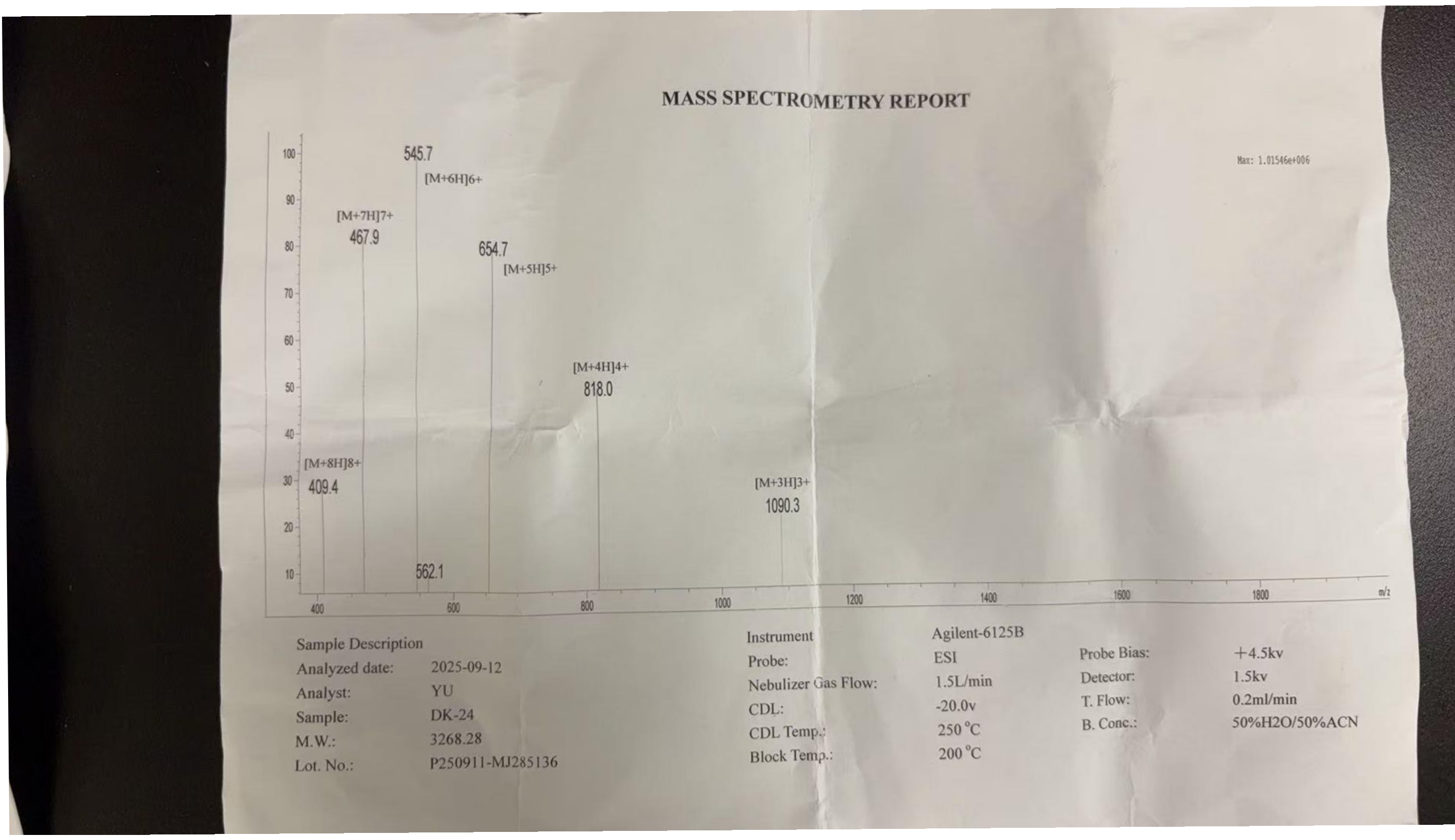

MASS SPECTROMETRY REPORT

Sample Description

Analyzed date:	2025-09-12
Analyst:	YU
Sample:	DK-24
M.W.:	3268.28
Lot. No.:	P250911-MJ285136

Instrument	Agilent-6125B
Probe:	ESI
Nebulizer Gas Flow:	1.5L/min
CDL:	-20.0v
CDL Temp.:	250 °C
Block Temp.:	200 °C
Probe Bias:	+4.5kv
Detector:	1.5kv
T. Flow:	0.2ml/min
B. Conc.:	50%H2O/50%ACN
