# Supplementary material for: Interferon regulatory factor 5 in myeloid cells promotes obstructive nephropathy-induced renal fibrosis in vivo
Source: JCI Insight. 2026 Aug 6;11(19):e201877. doi: 10.1172/jci.insight.201877 (PMC13726410; doi:10.1172/jci.insight.201877)

Figure 5D   VIM and α-SMA

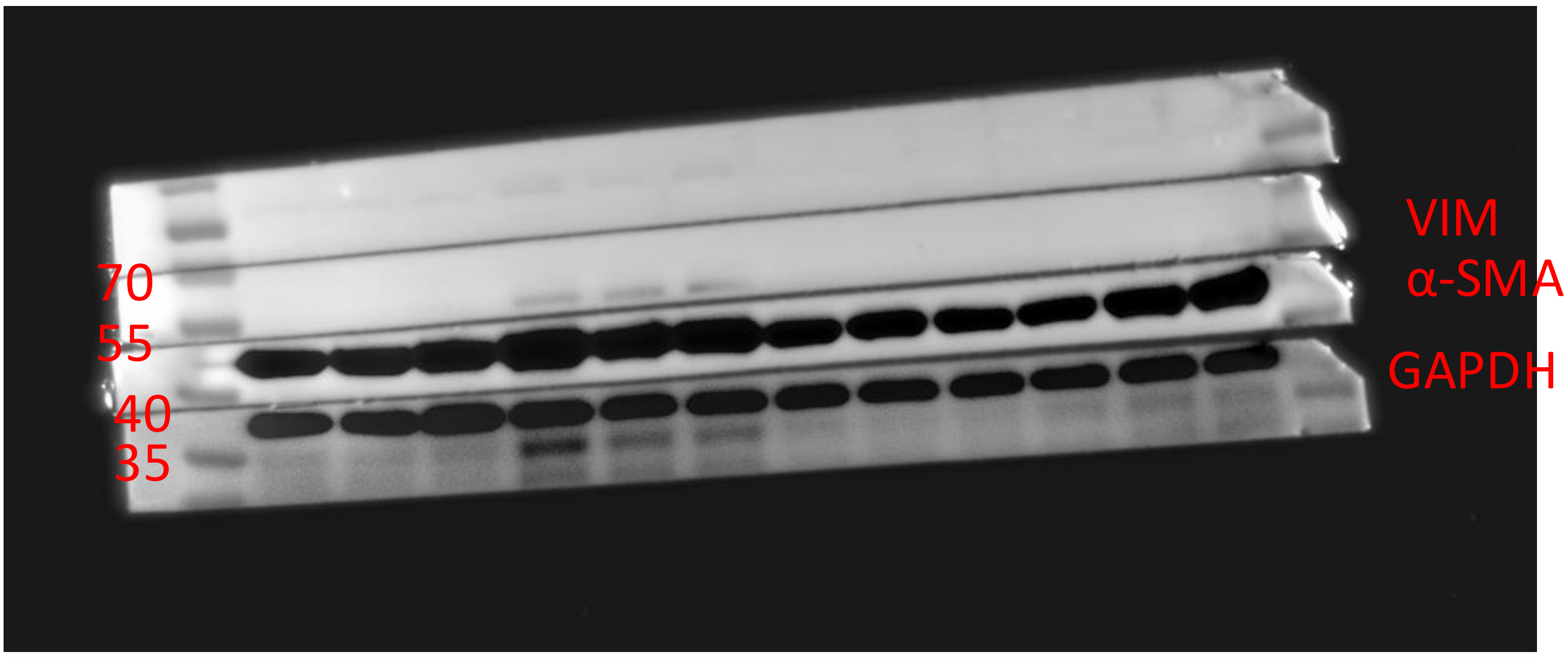

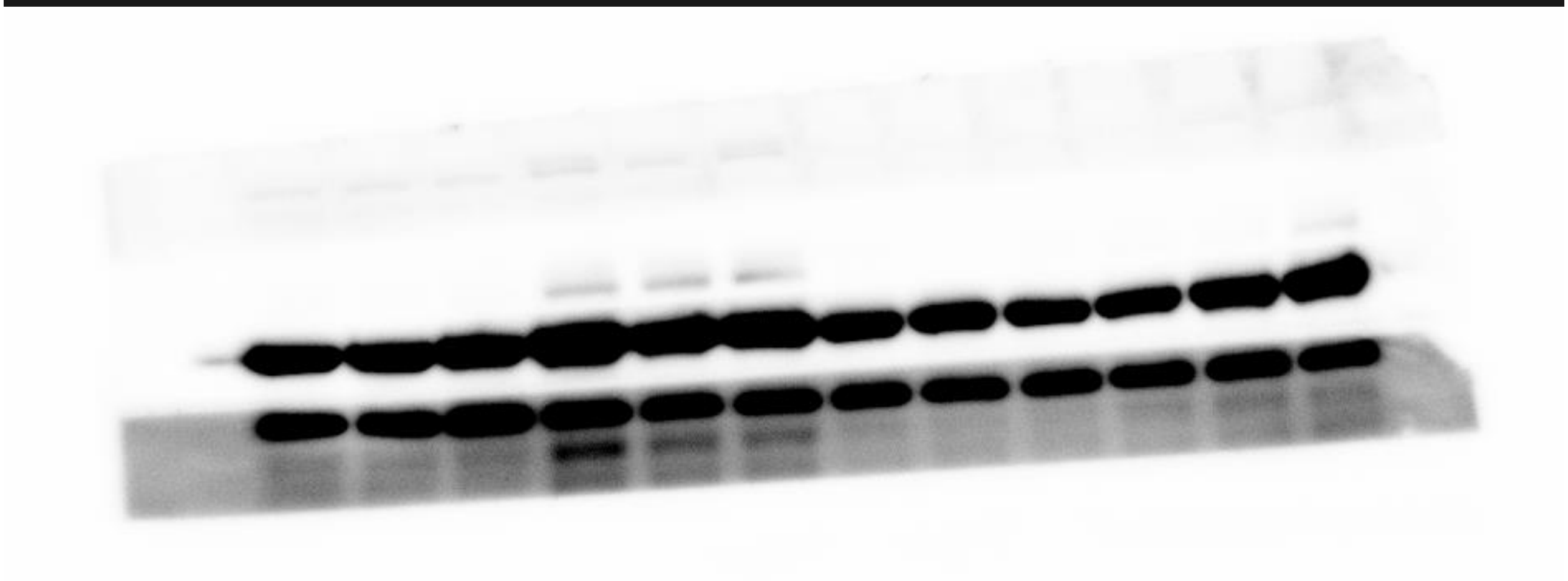

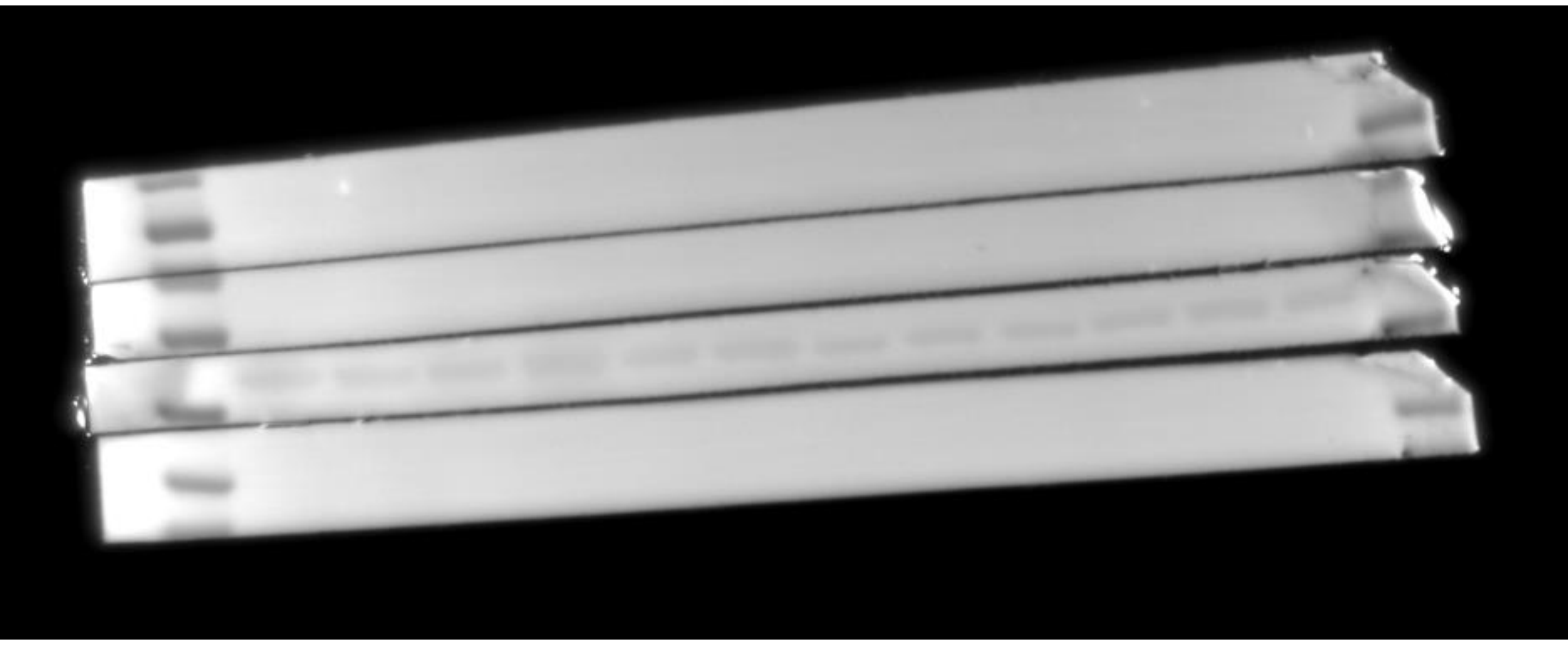

Figure 7E α-SMA

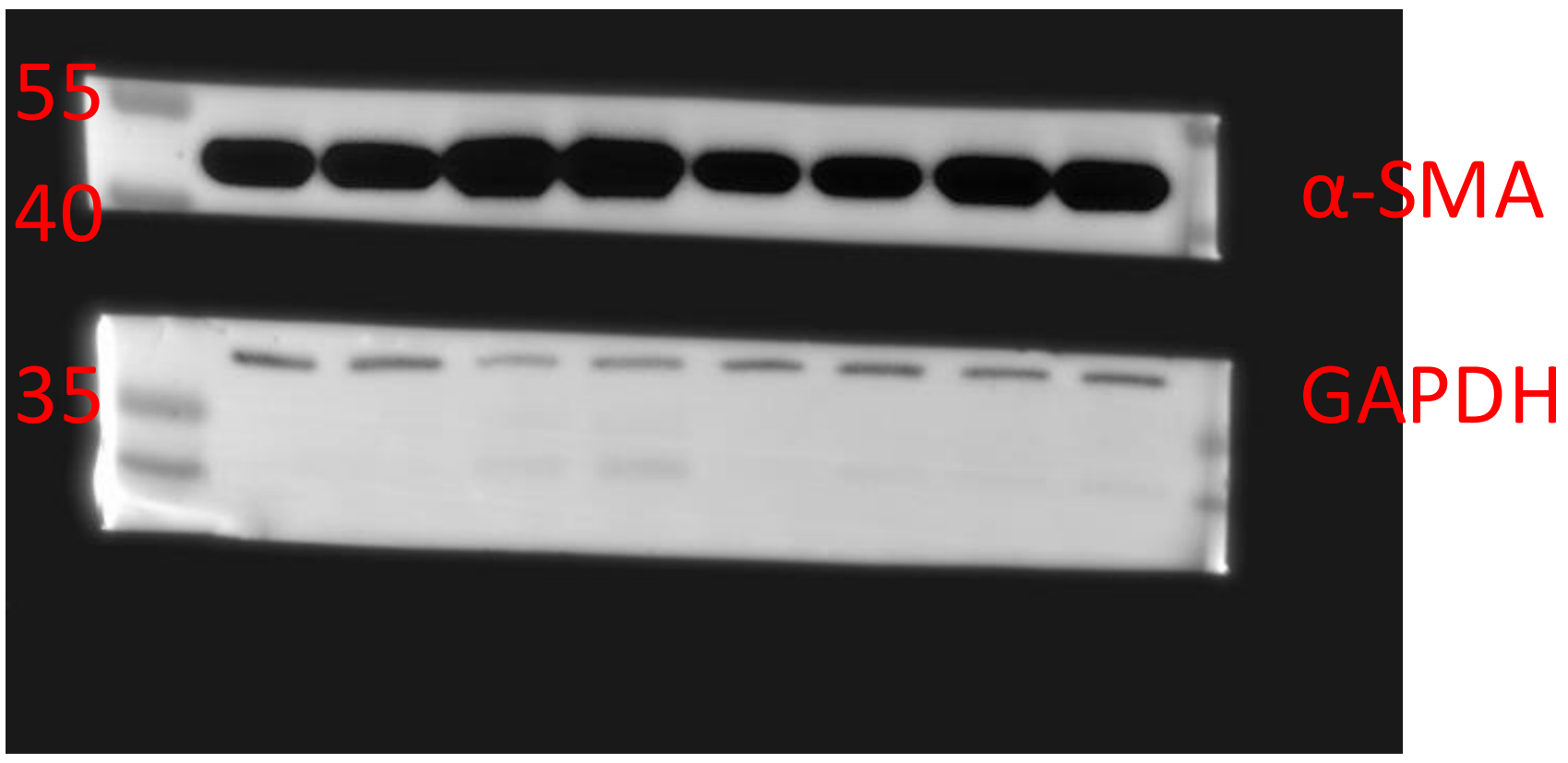

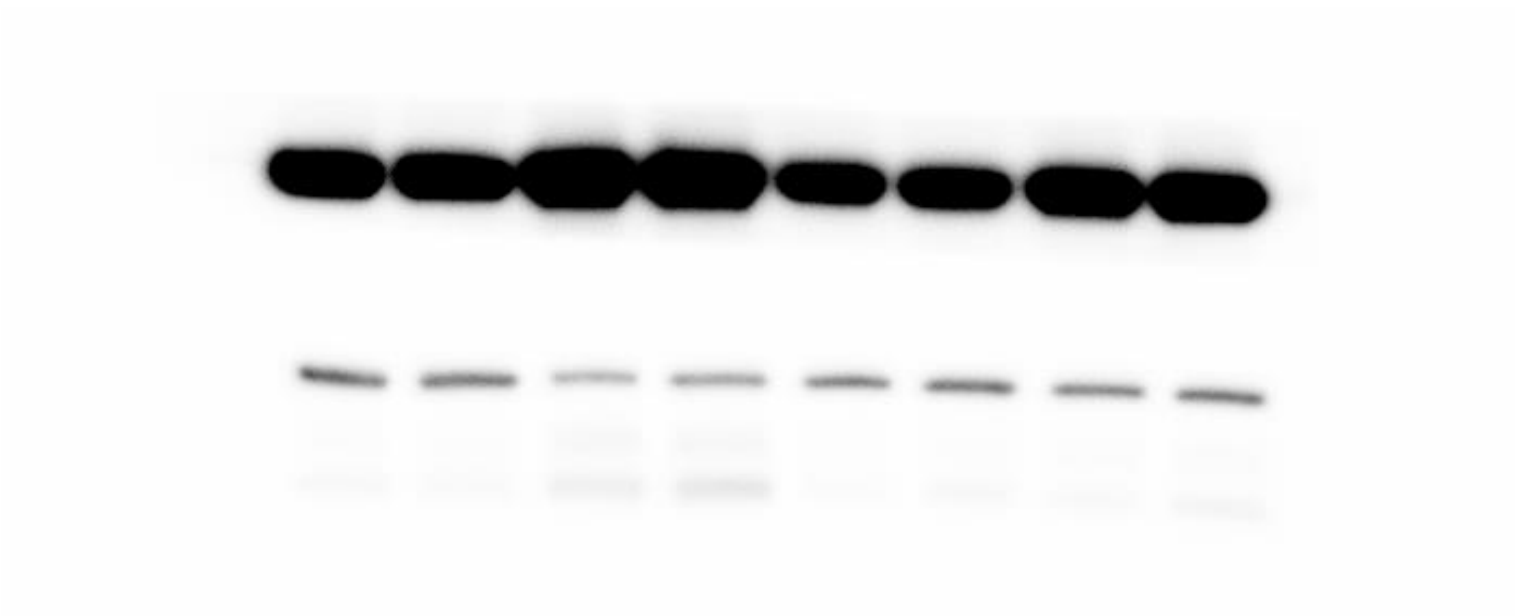

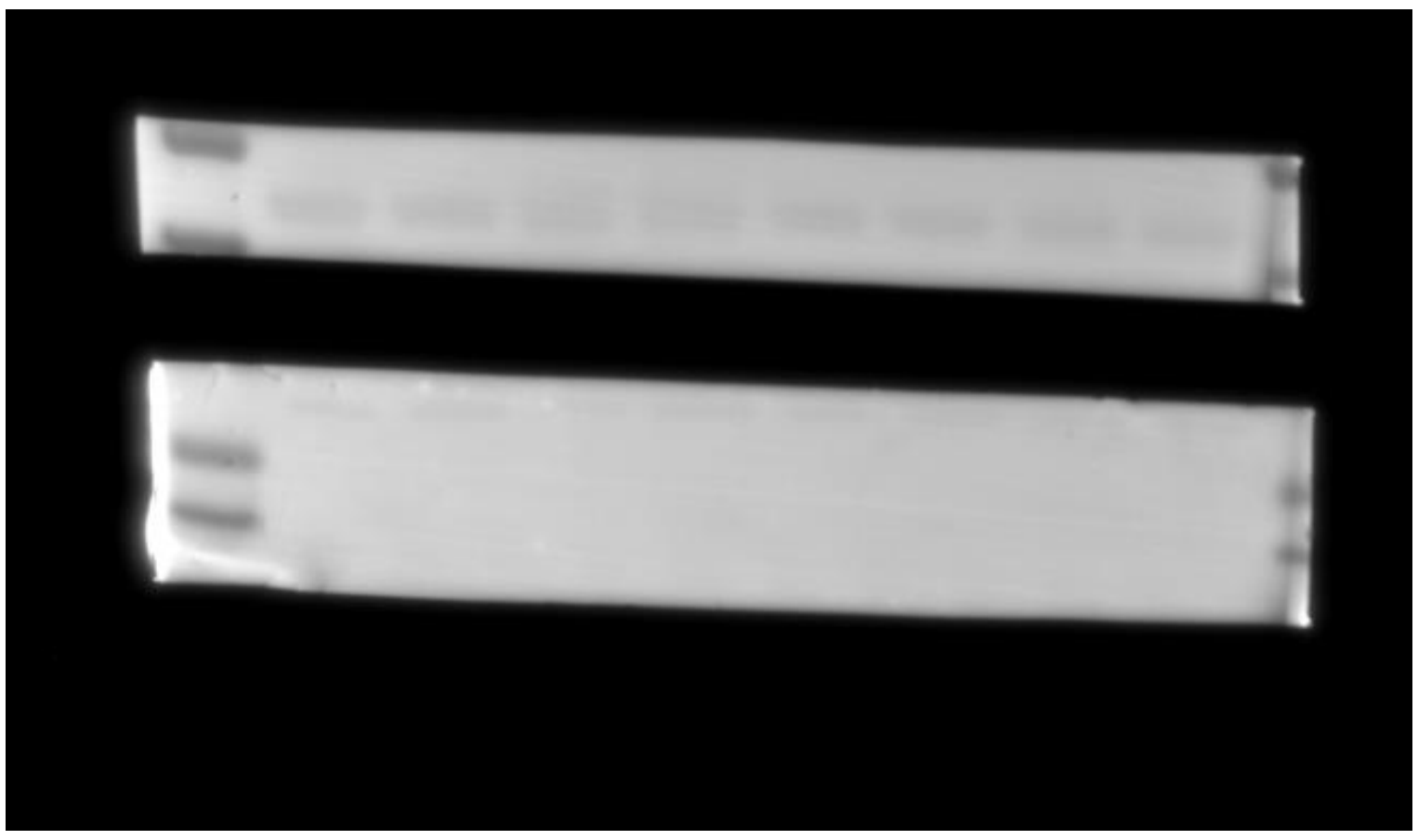

Figure 7E VIM

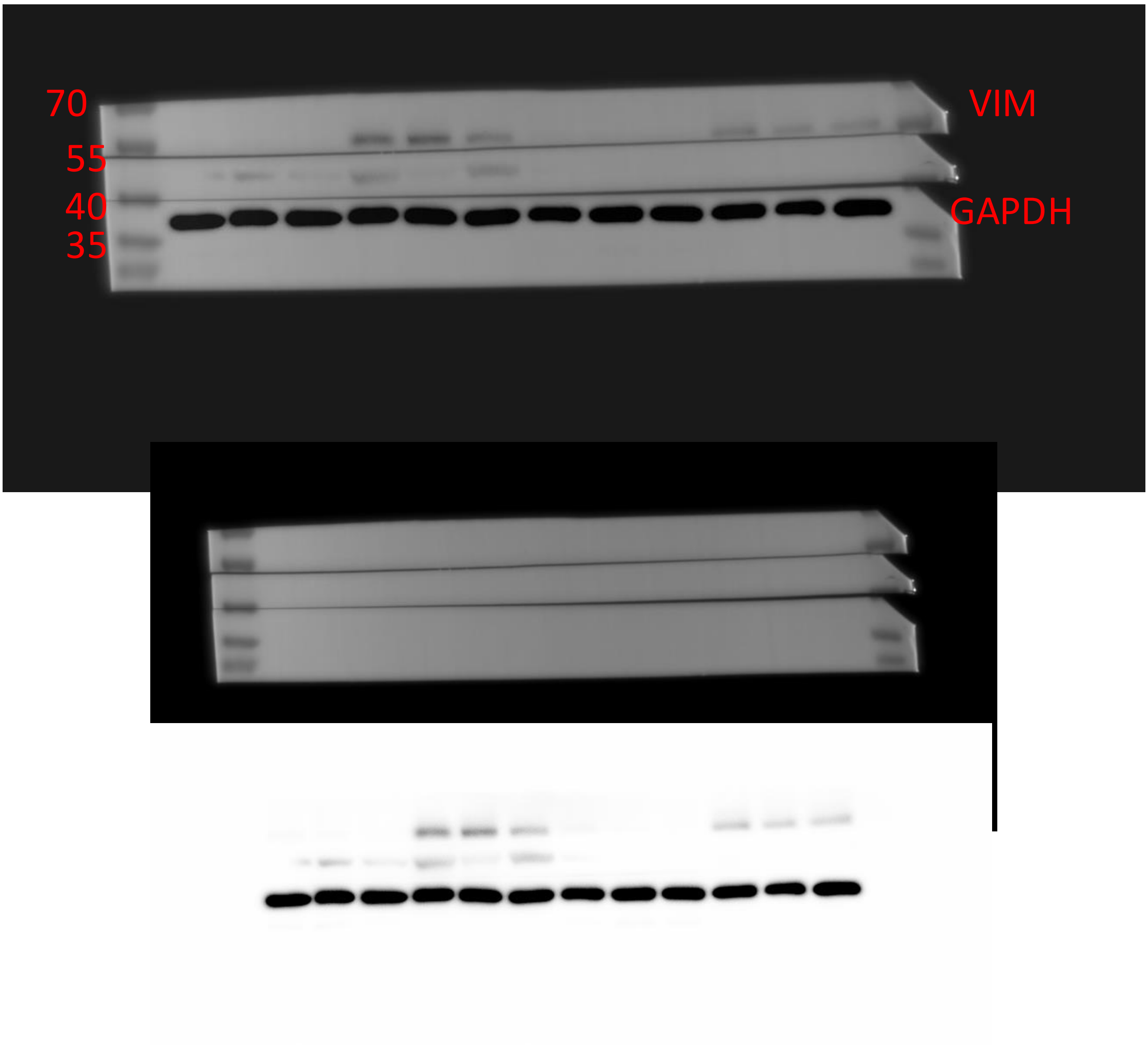

VIM bands obtained after adjusting the exposure time

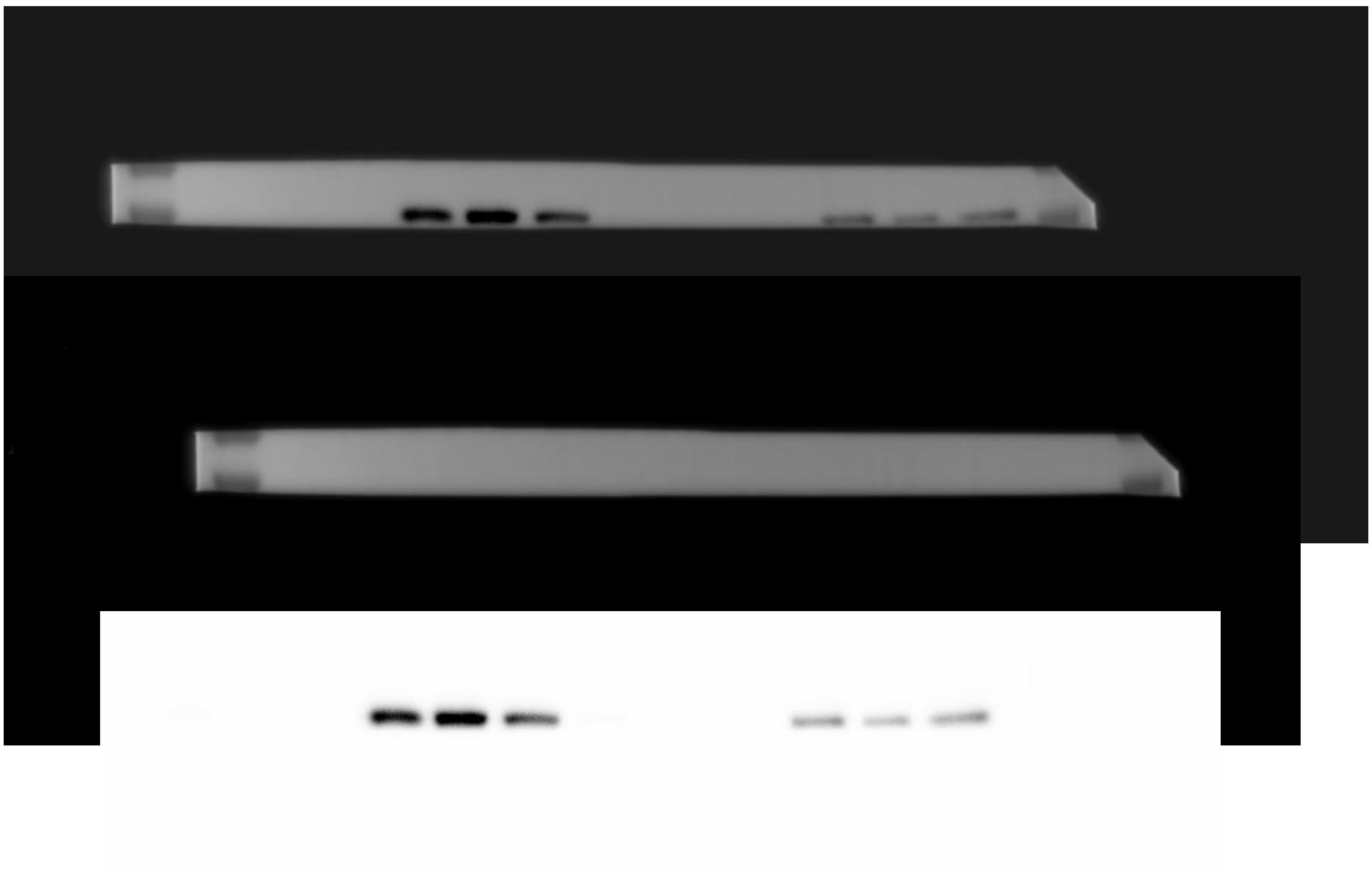

Figure 9D MMP9

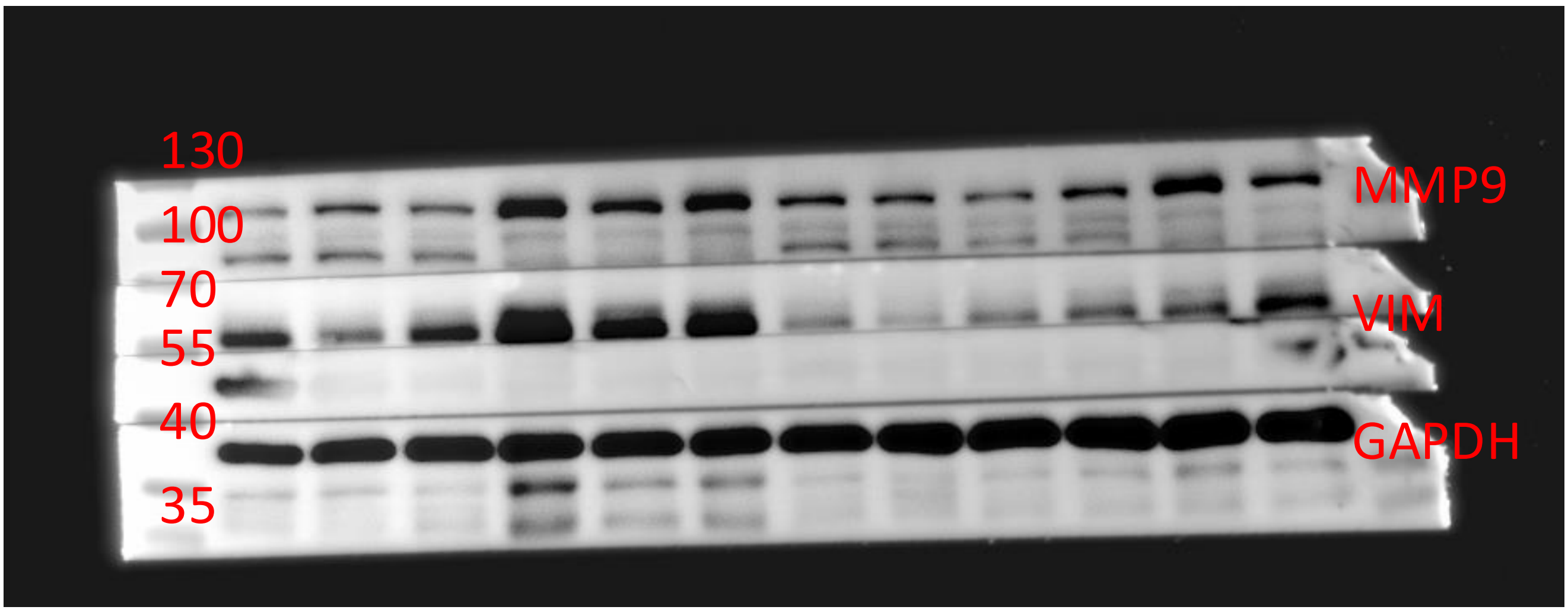

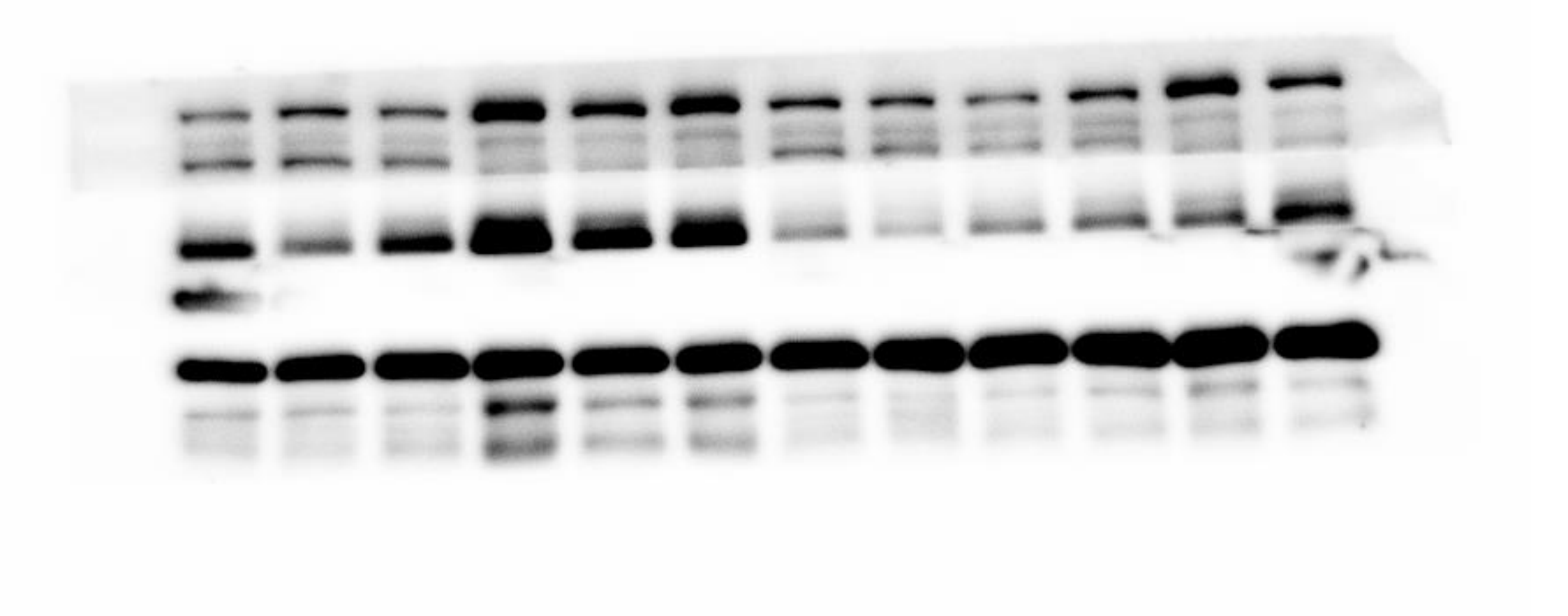

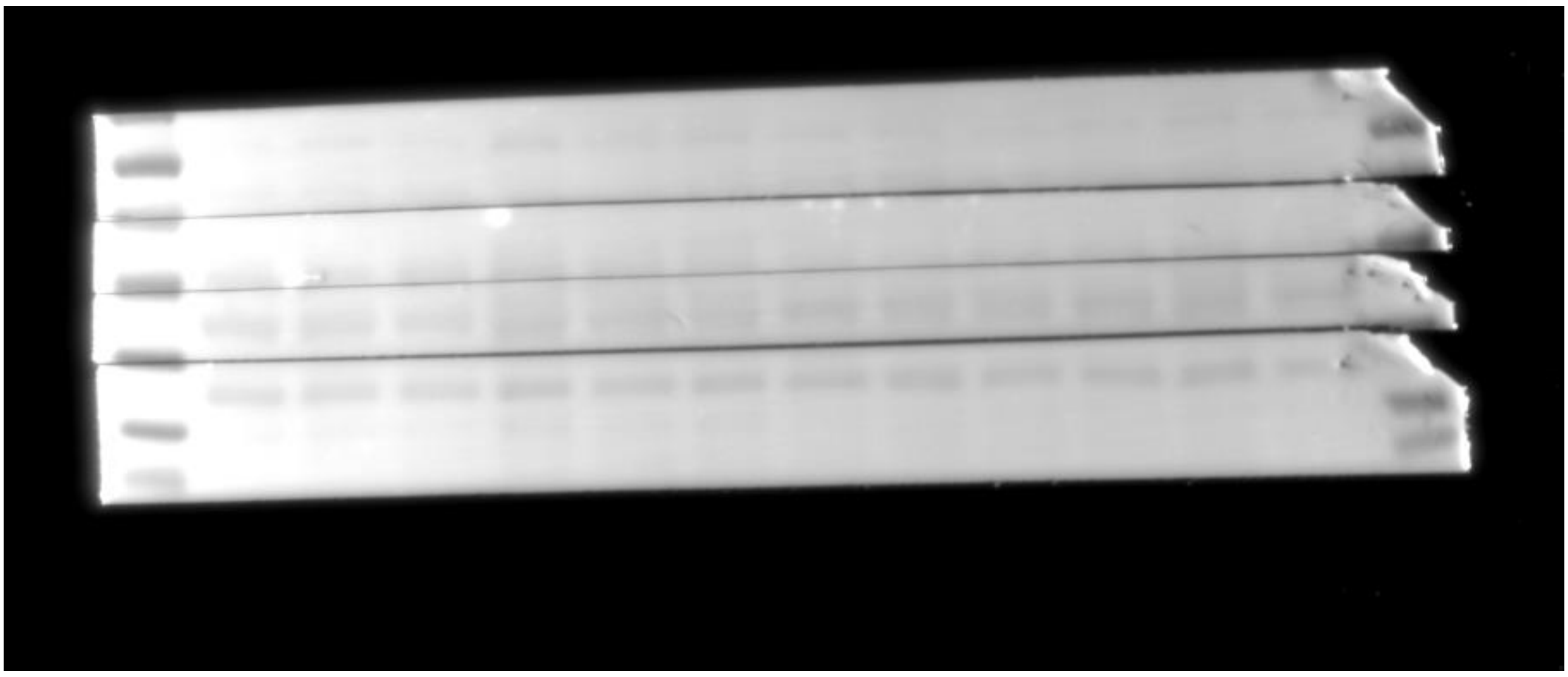

Figure 8C F4/80 and TNF-α and supplementary figure 3B IRF5

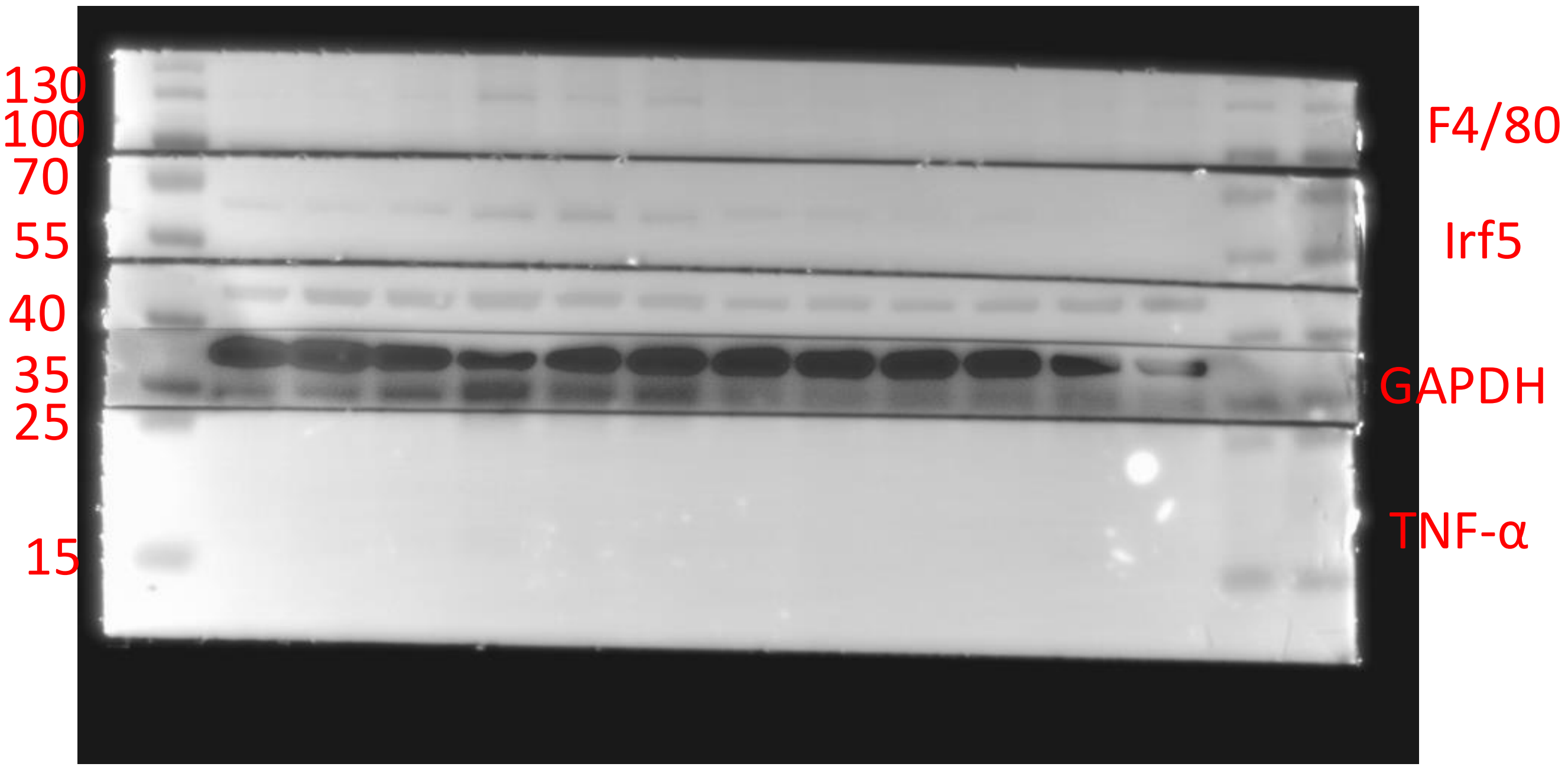

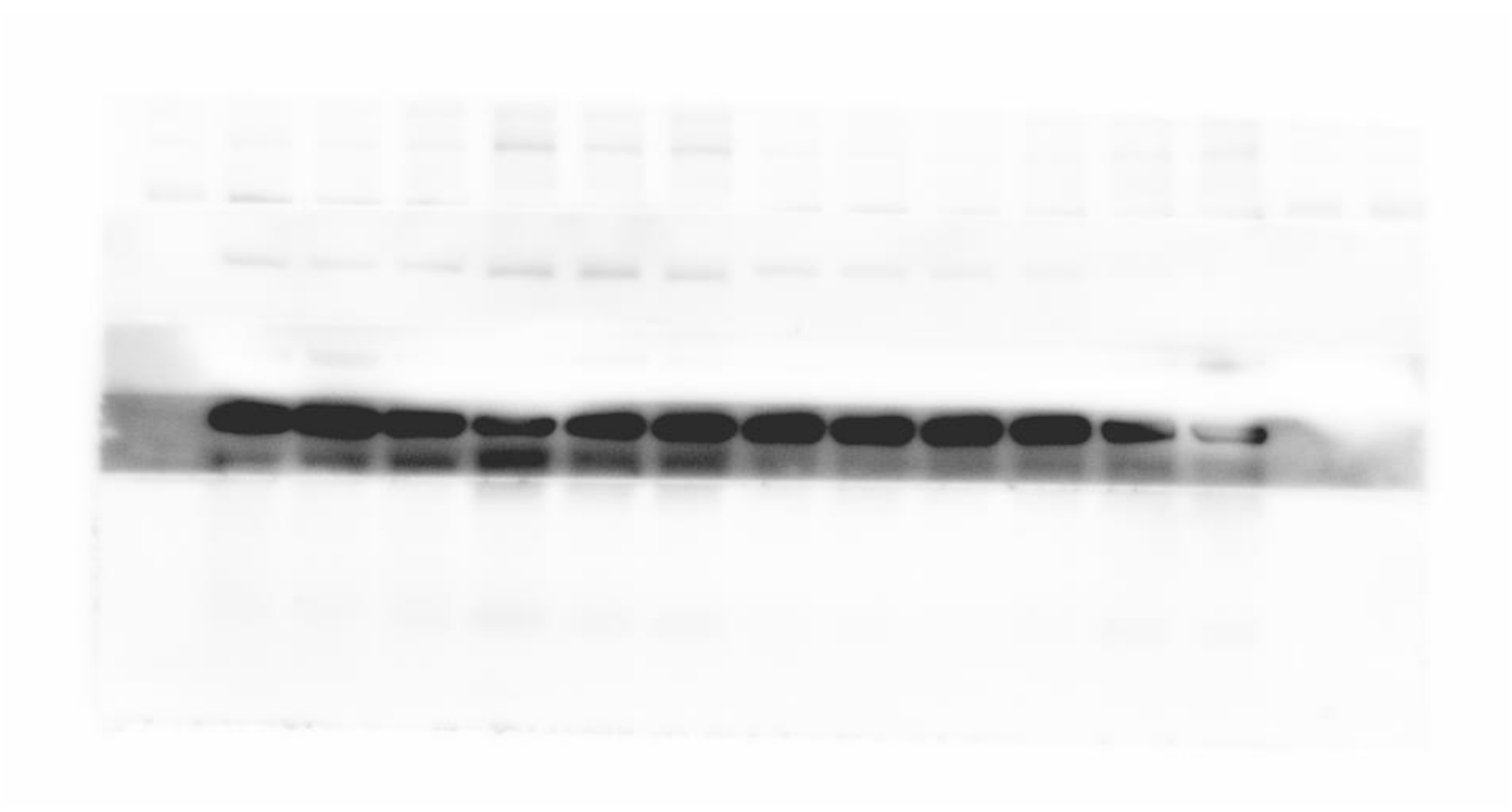

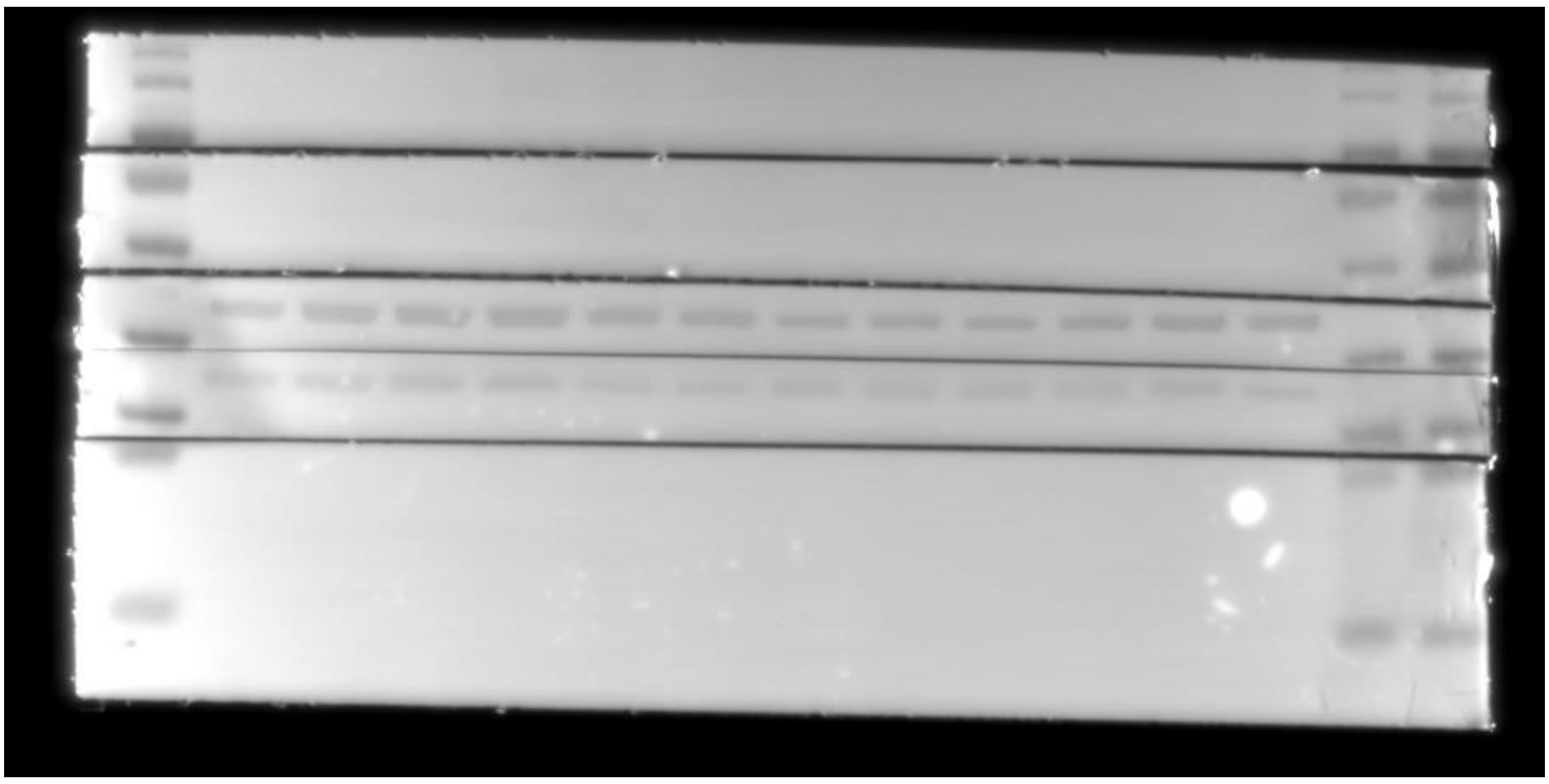

Figure 8C   IL-1β

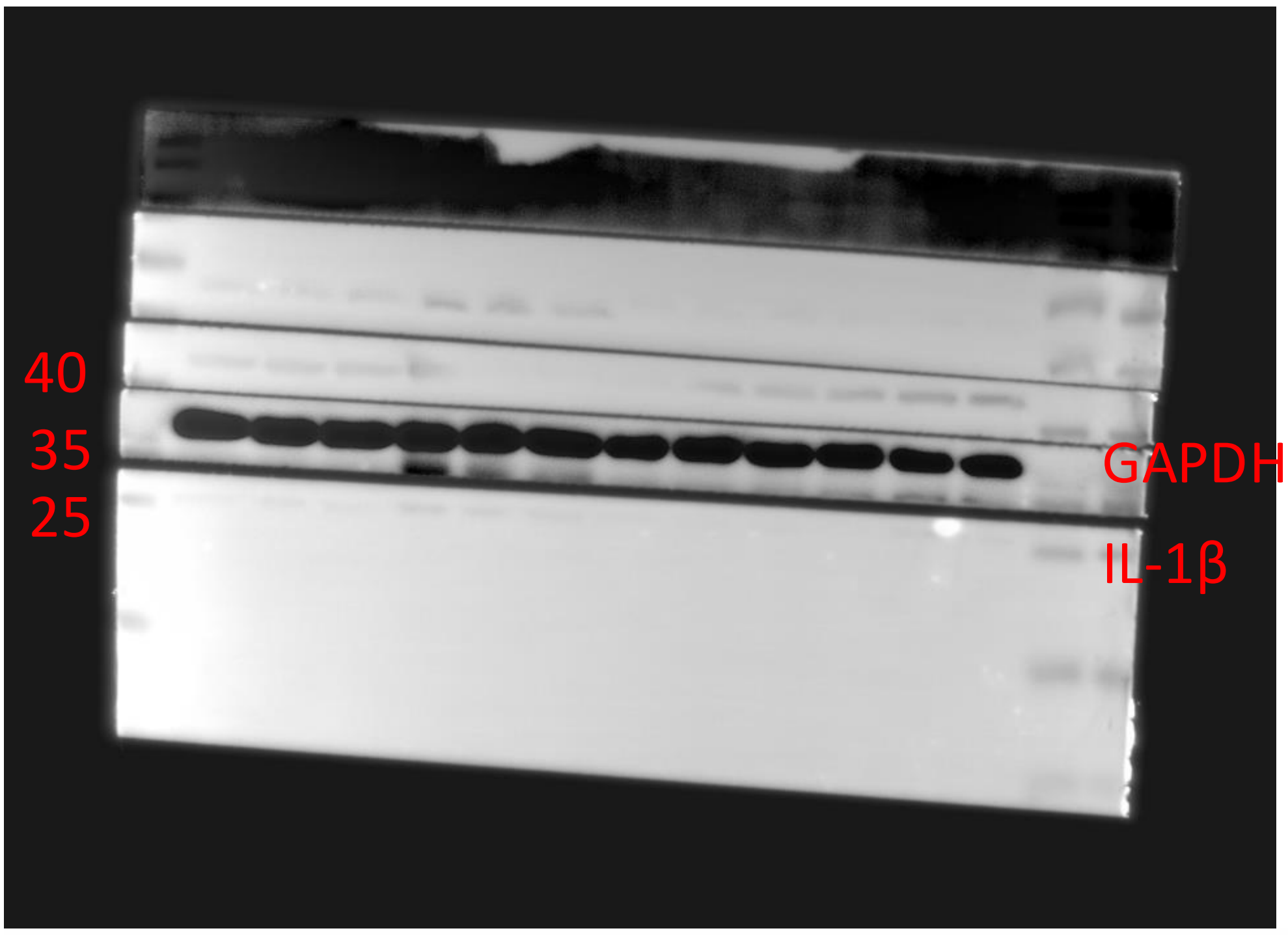

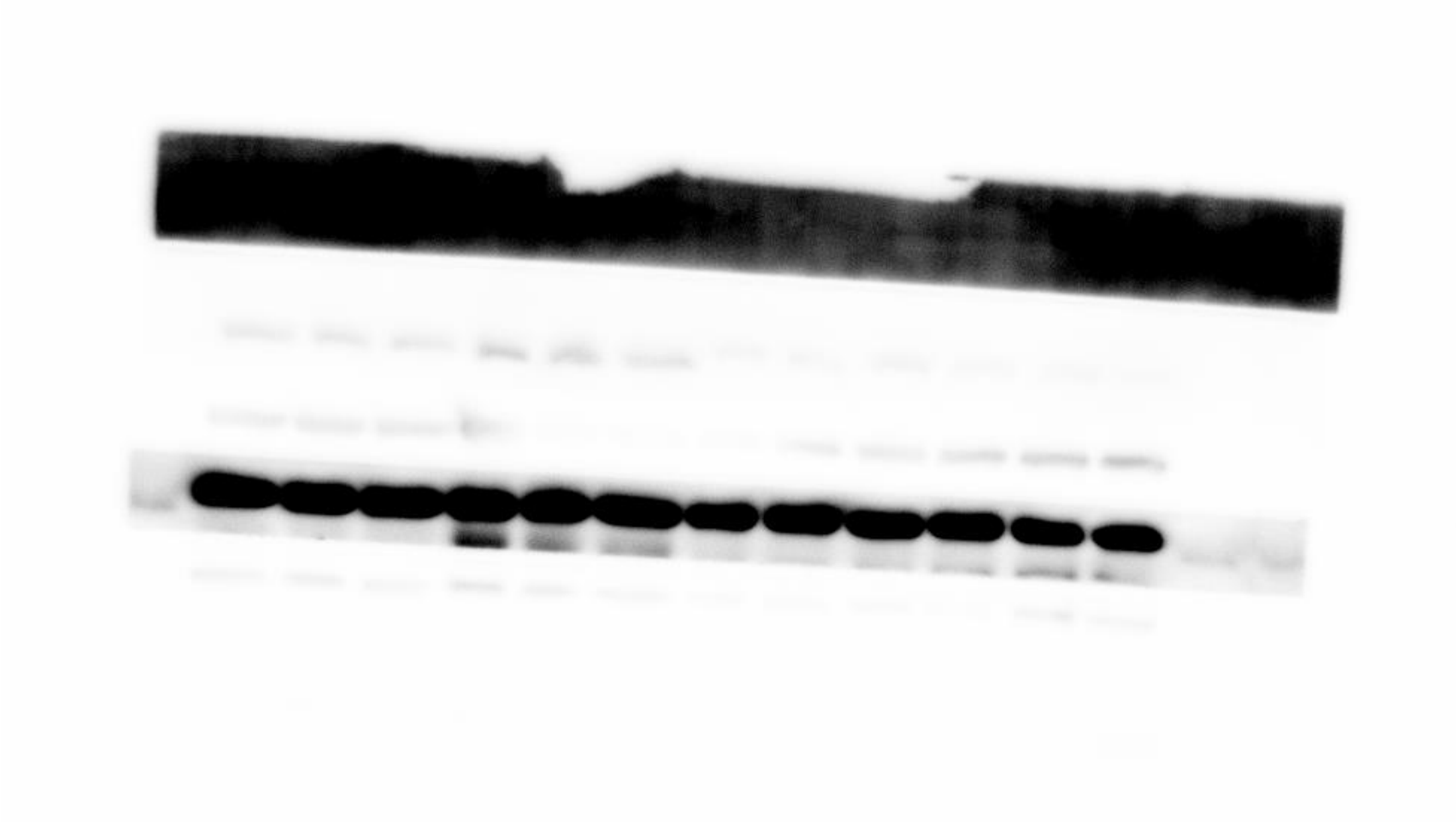

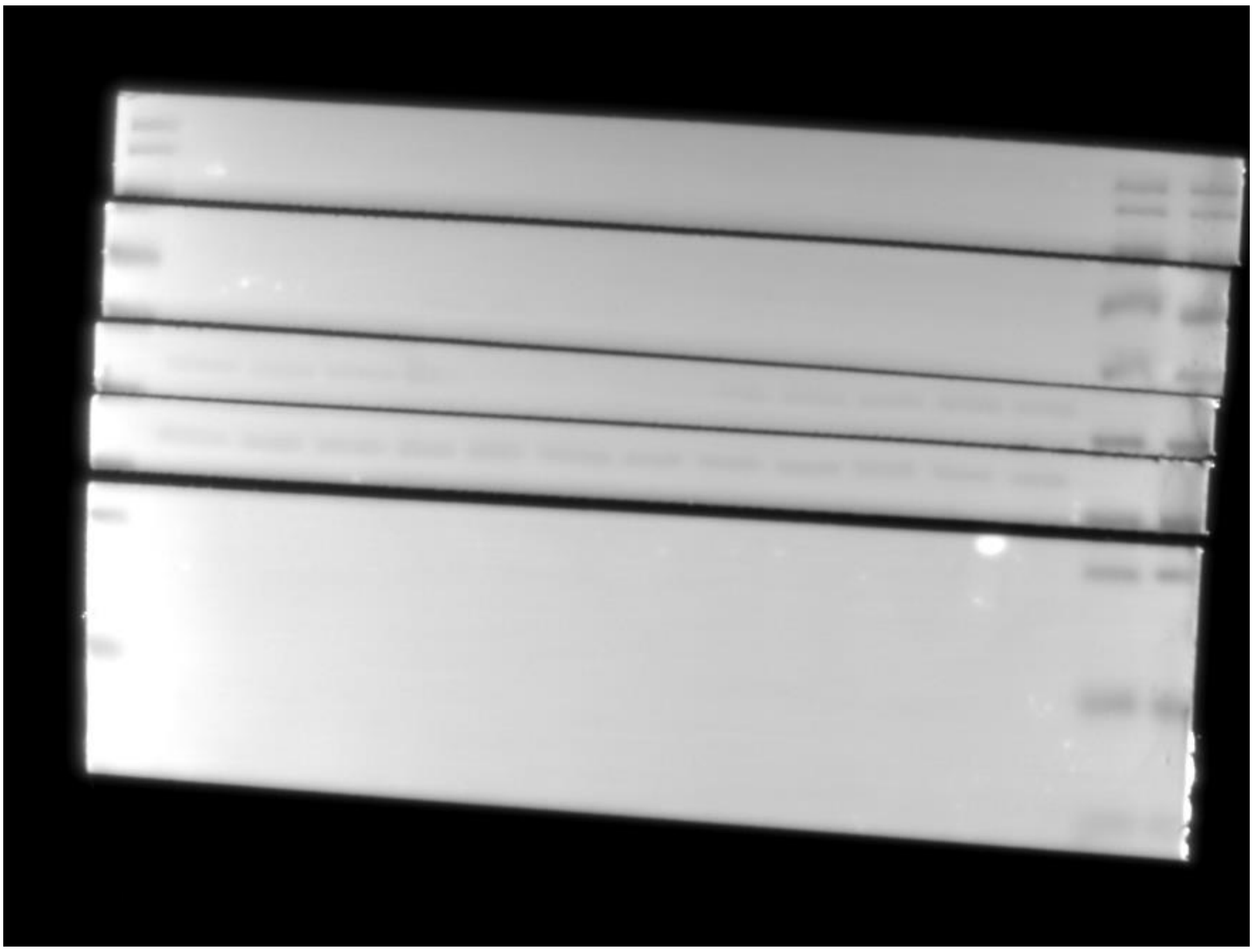

Figure 10 D

MMP9

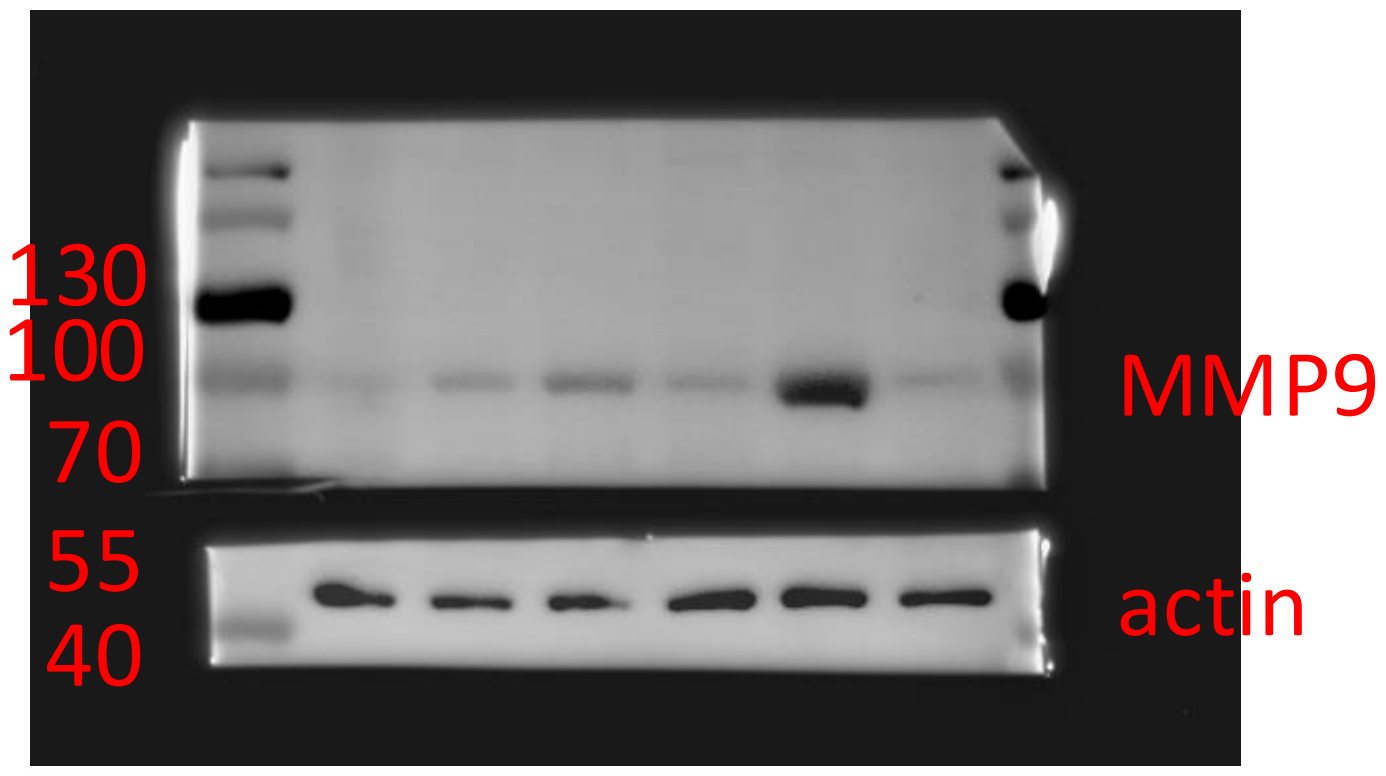

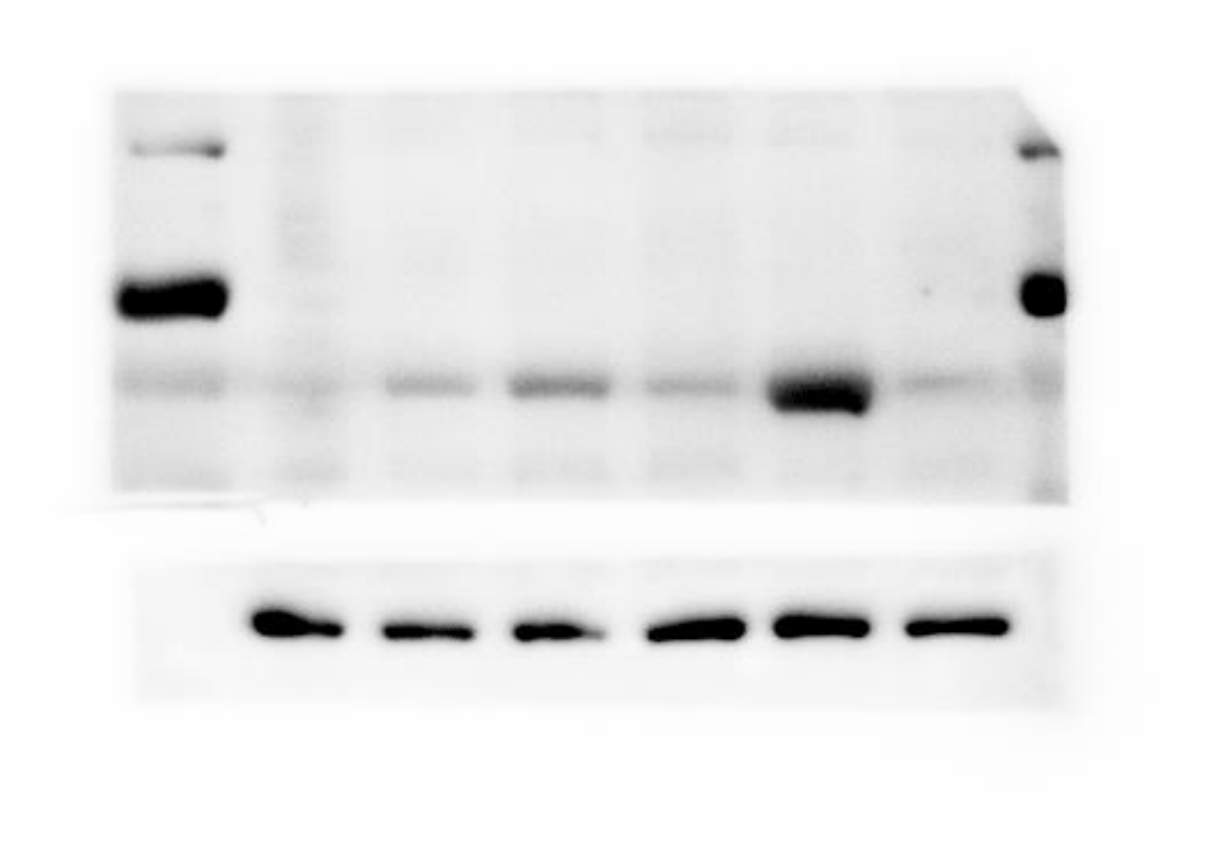

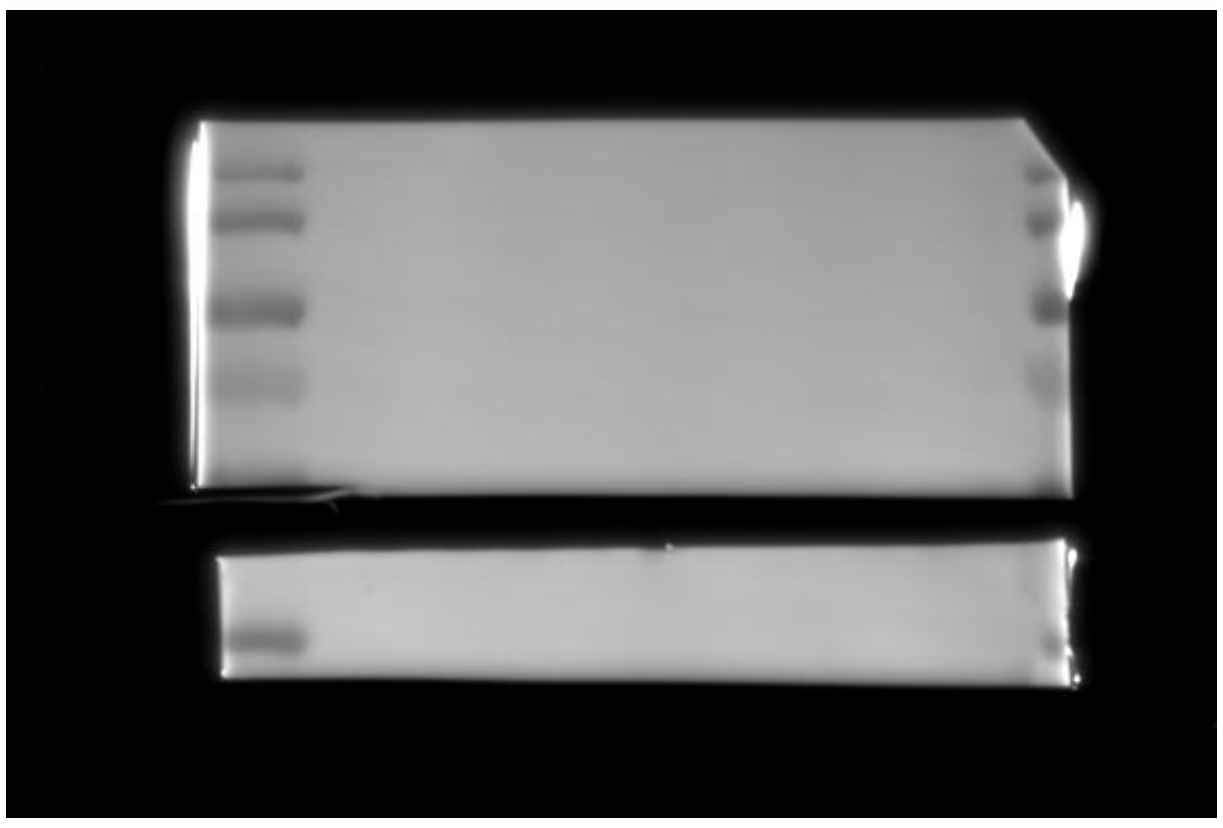

supplementary figure 3C IRF5

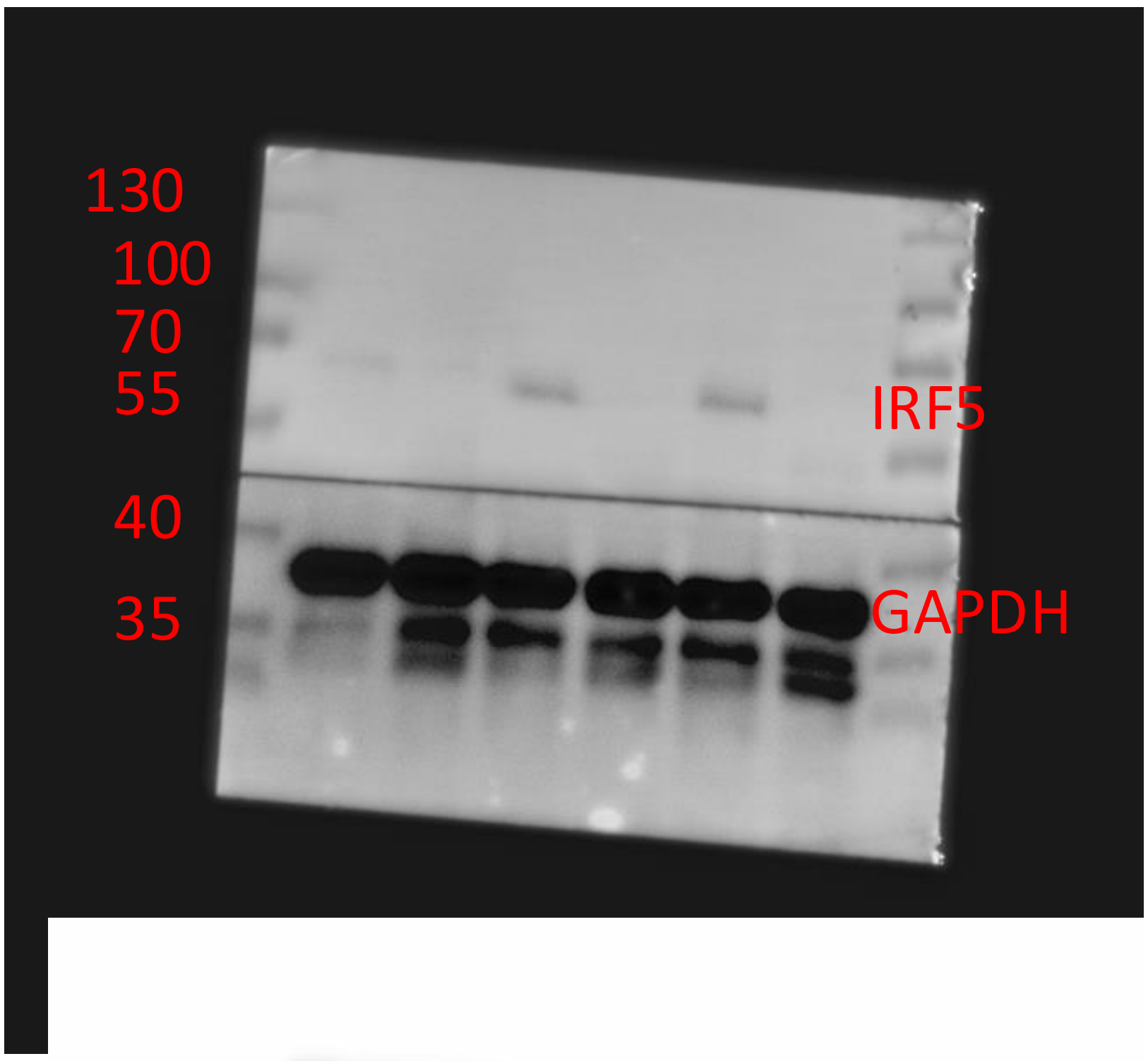

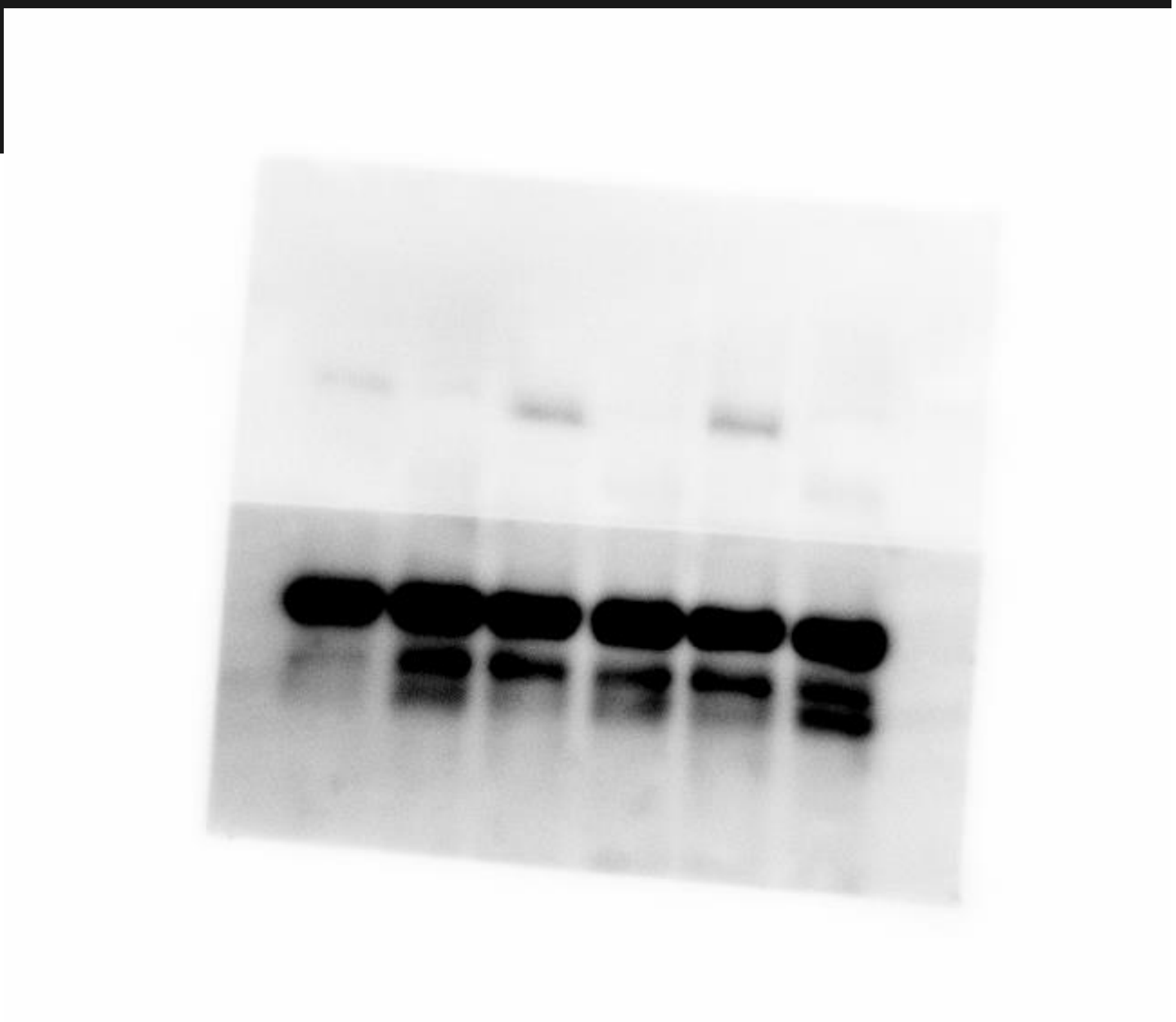

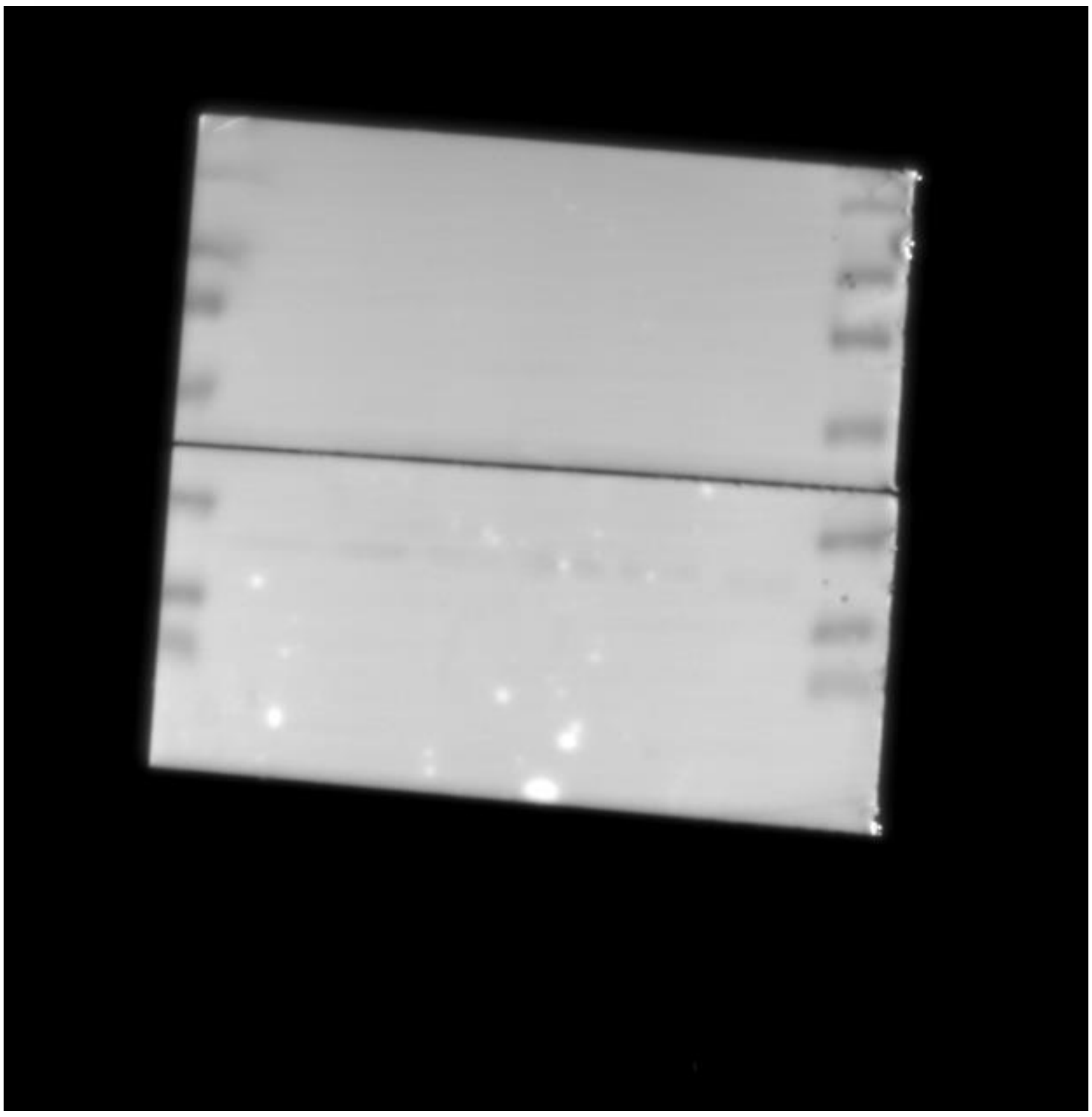

Figure 12 F

MMP9

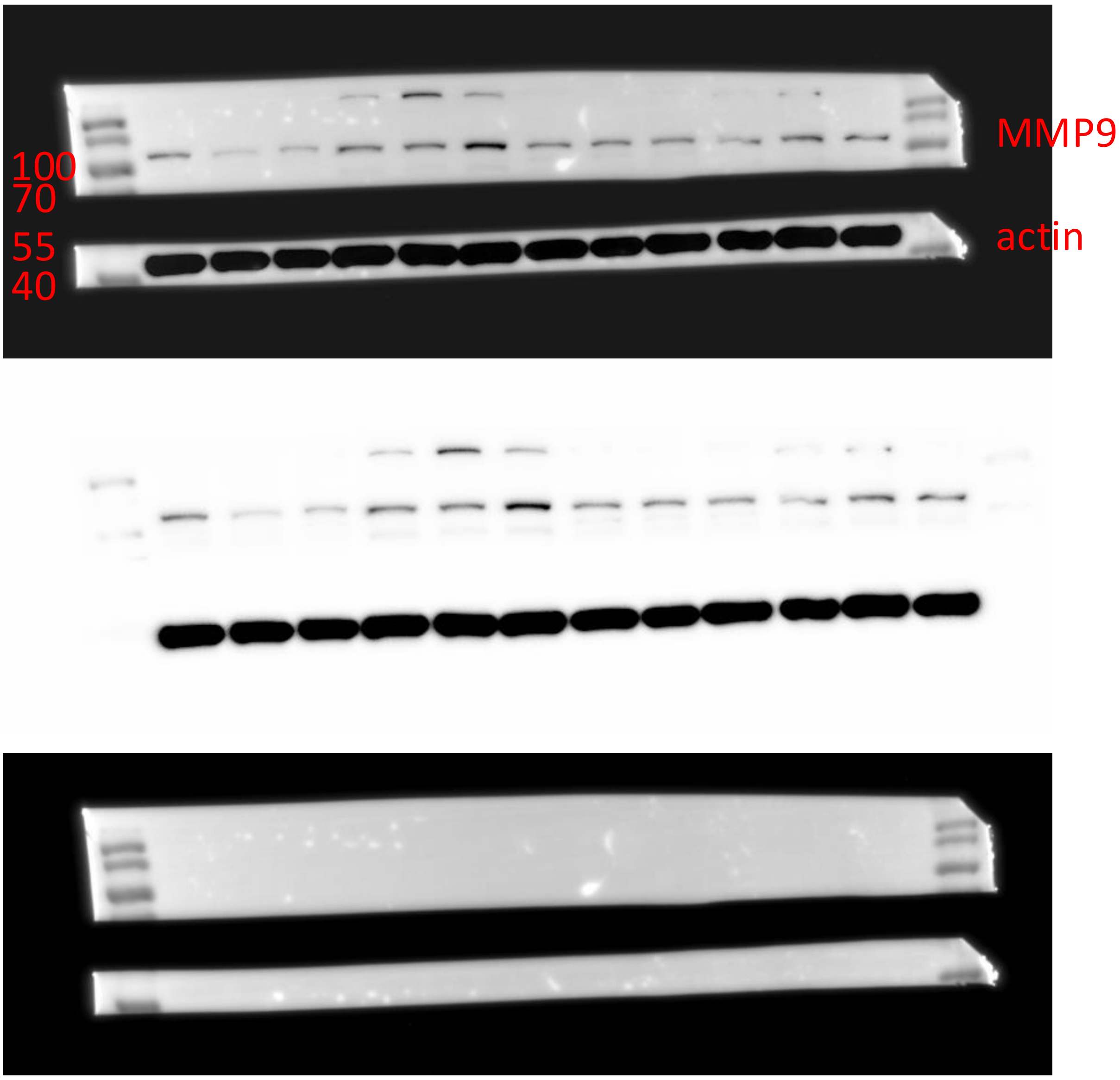

Figure 12F α-SMA

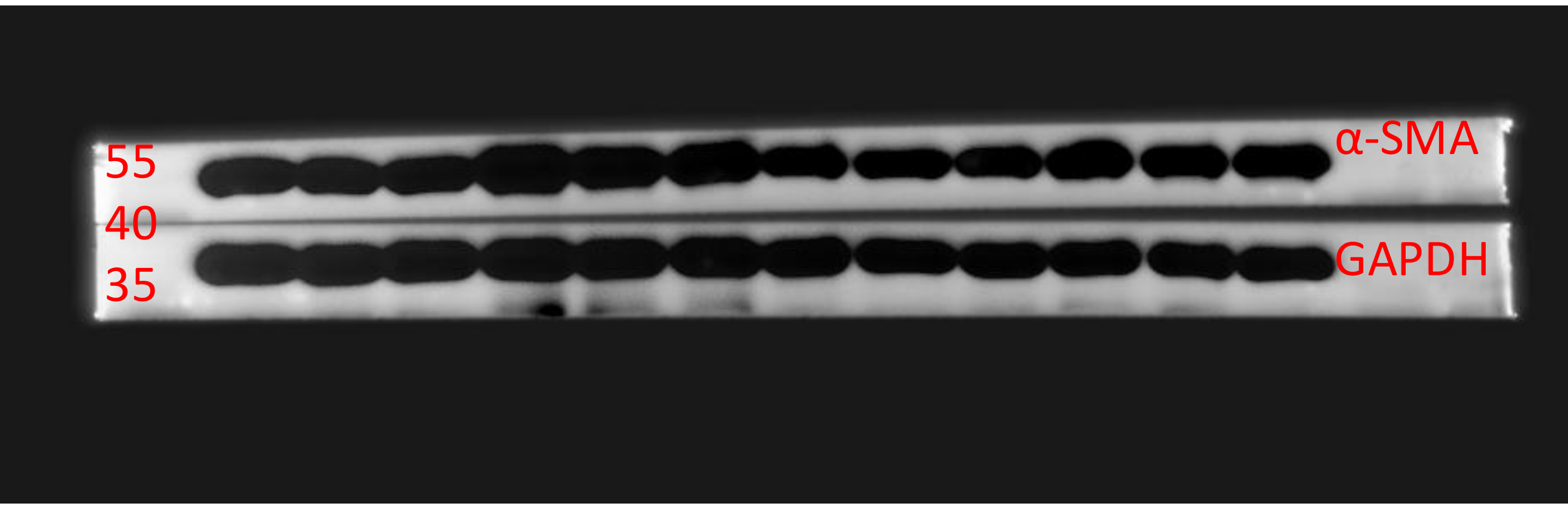

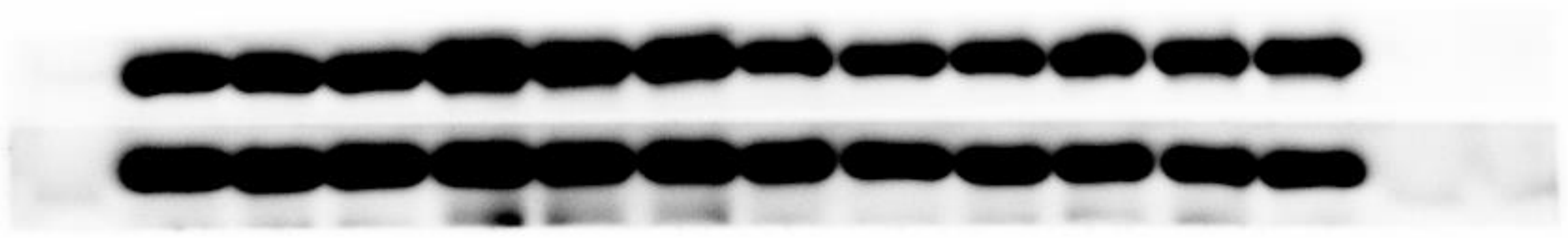

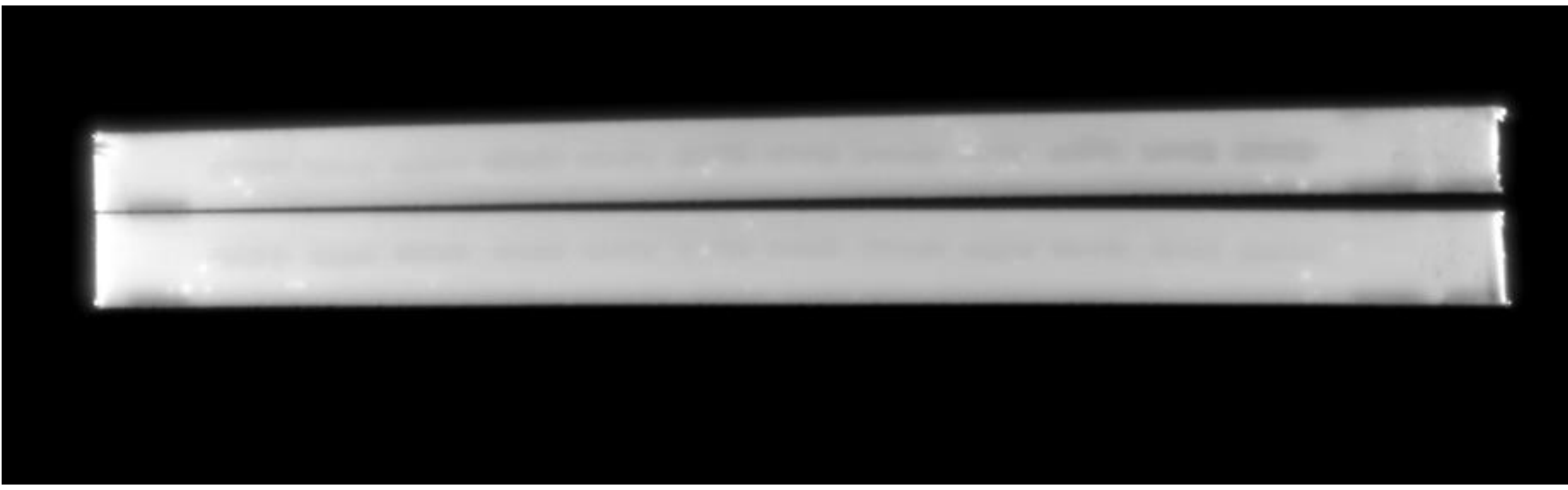

supplementary figure 3A IRF5

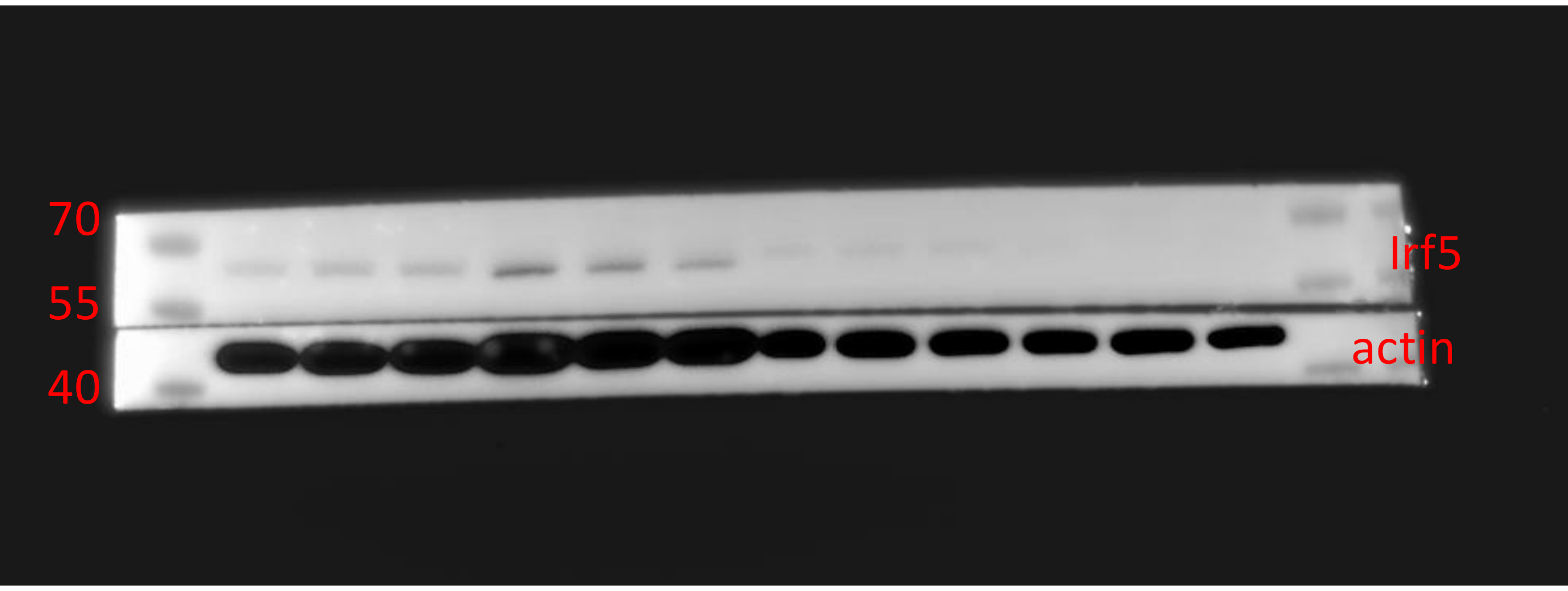

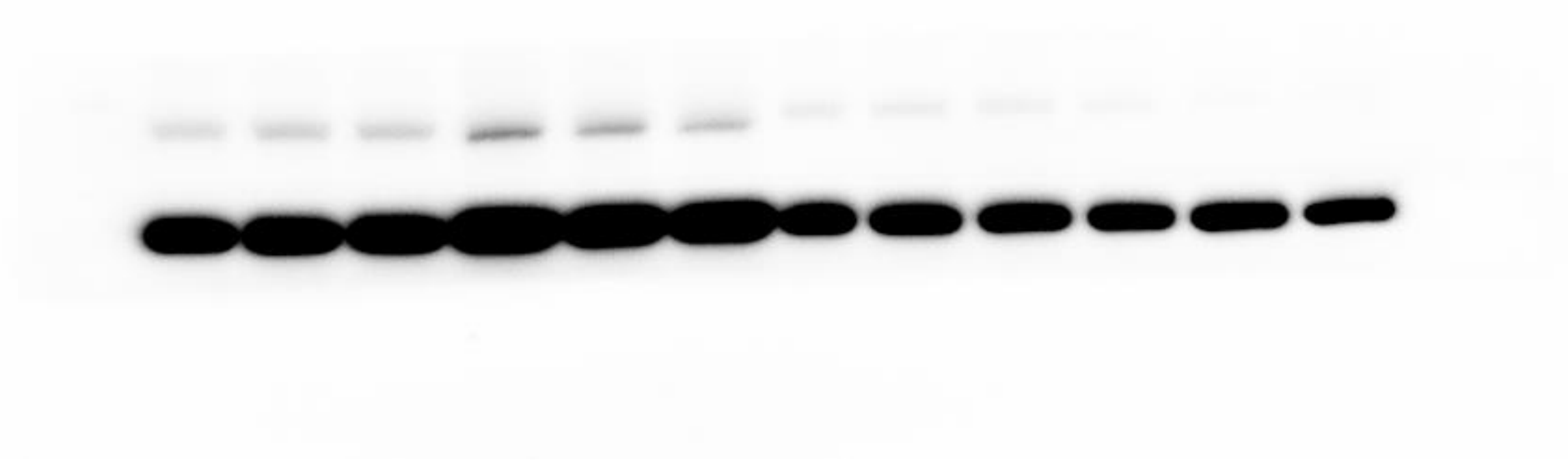

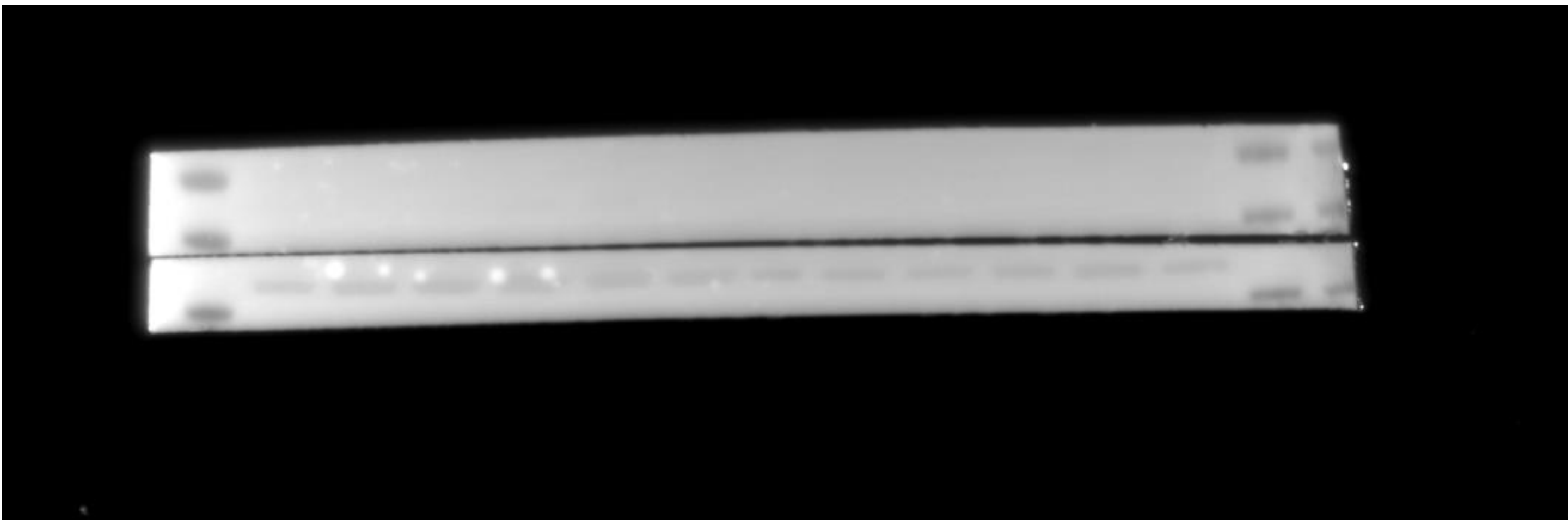

supplementary figure 3D IRF5

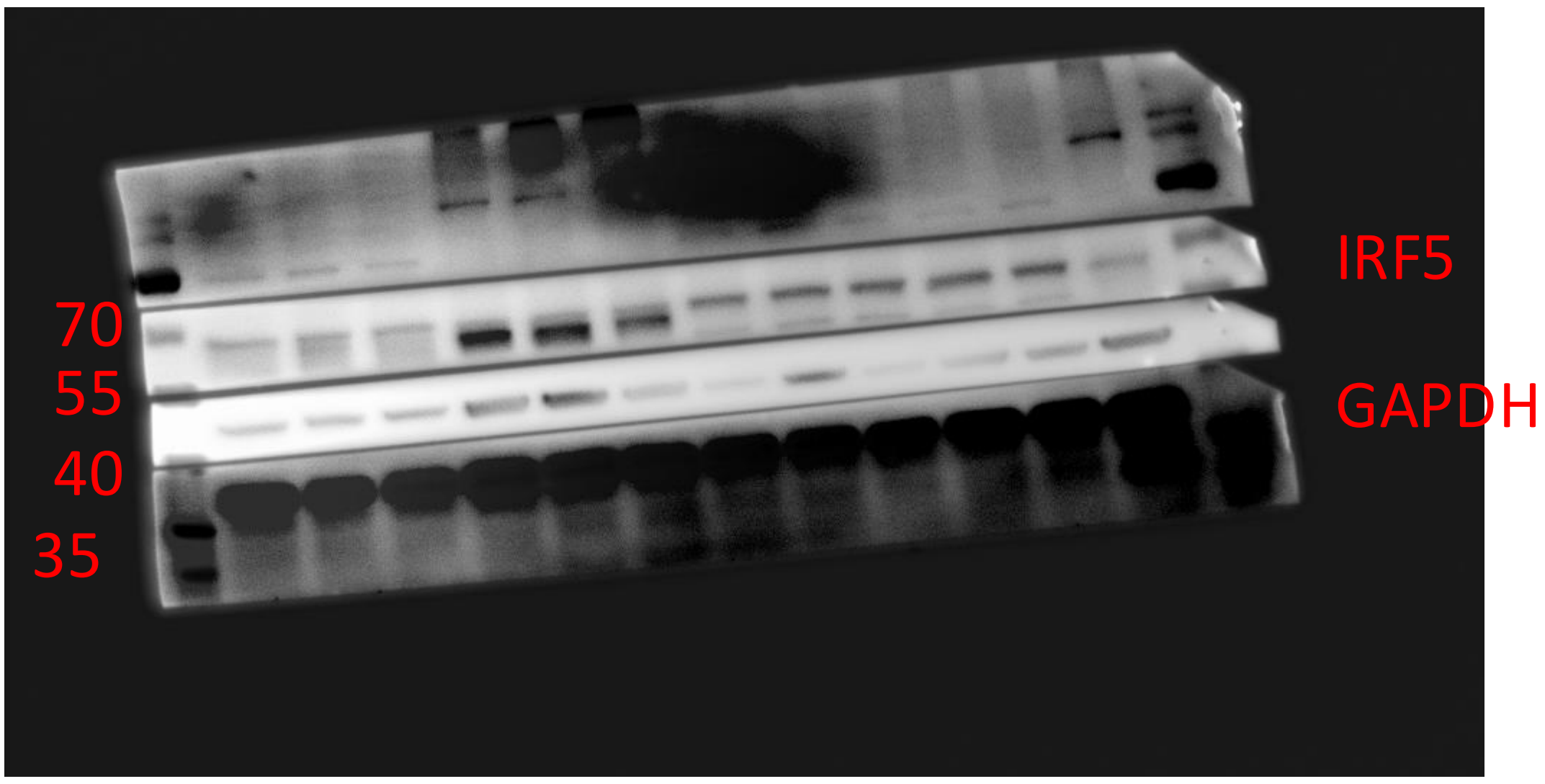

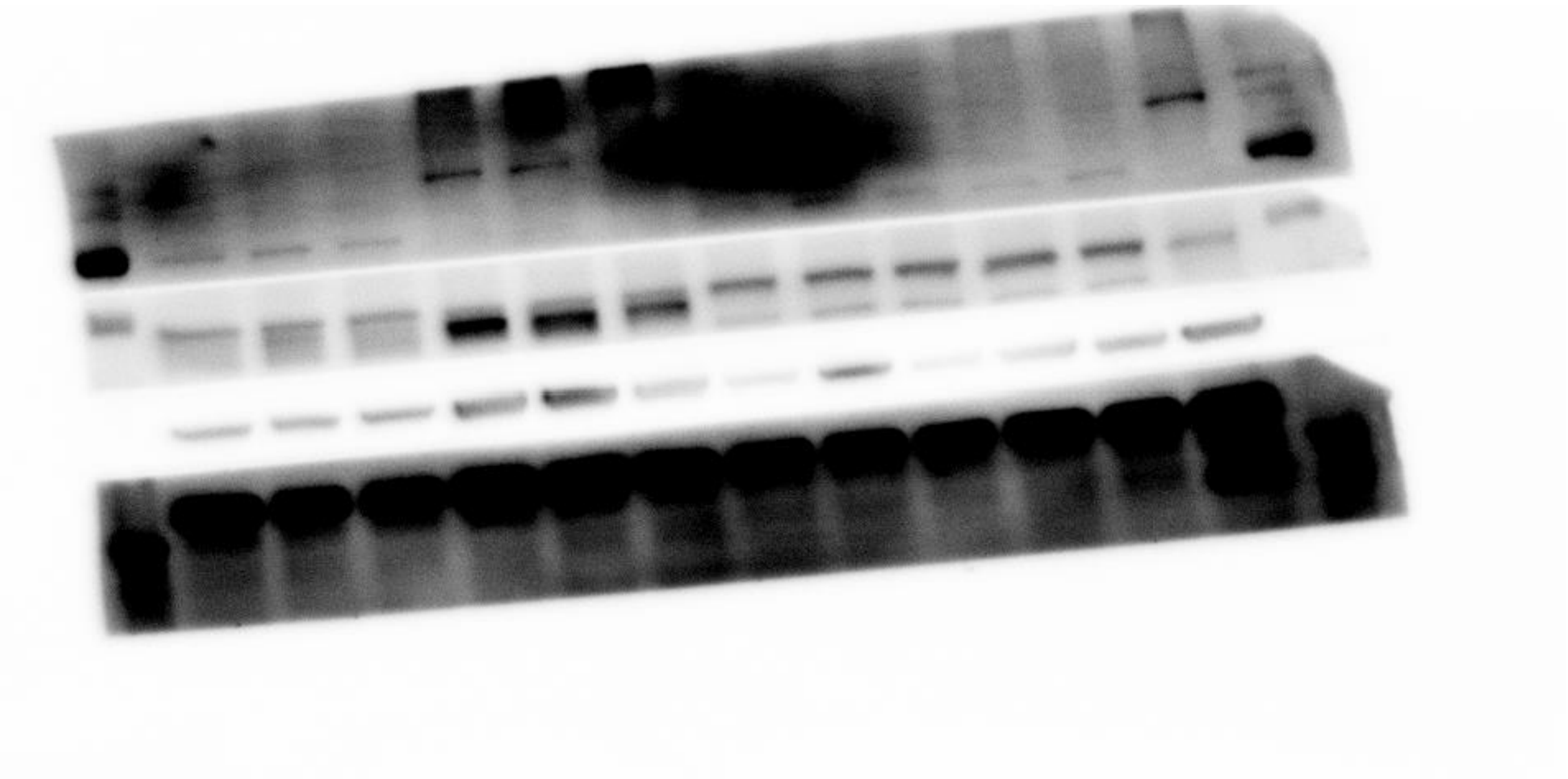

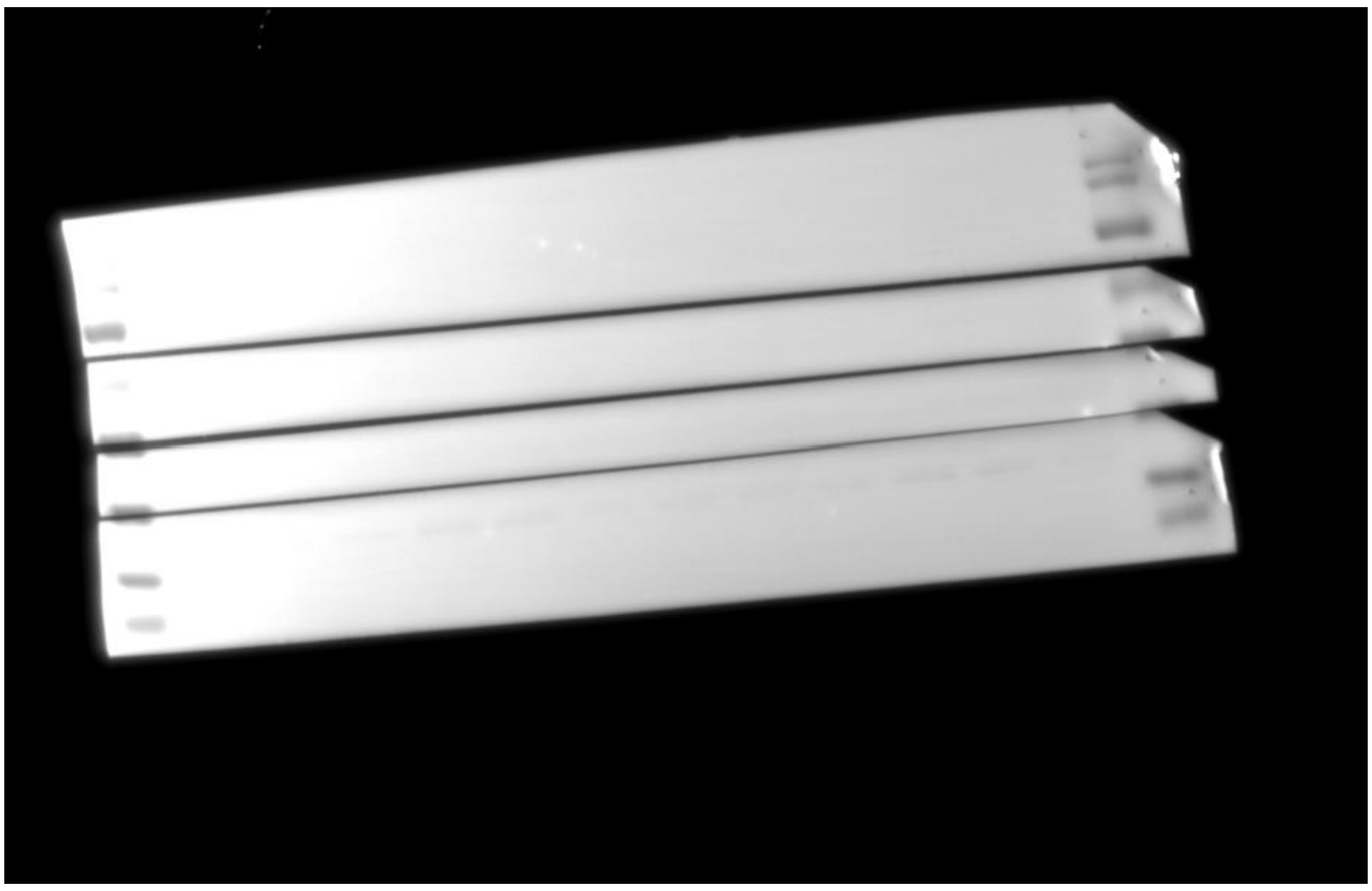

Supplement: Supplementary file 2 [file jciinsight-11-201877-s310.pdf]
