# Supplementary material for: Interferon regulatory factor 5 in myeloid cells promotes obstructive nephropathy-induced renal fibrosis in vivo
Source: JCI Insight. 2026 Aug 6;11(19):e201877. doi: 10.1172/jci.insight.201877 (PMC13726410; doi:10.1172/jci.insight.201877)
Supplement: Supplementary file 3 [file jciinsight-11-201877-s311.pdf]

Supplemental Table 1. Background information on the kidney fibrosis patients and normal controls

Kidney fibrosis patients (n=30)

			Number(n)	Percentage(%)
Age (years/Mean ± SD)	<18	3.0 ± 1.2	4	13.3%
	≥18	60.2 ± 8.7	26	86.7%
Gender	Male		17	56.7%
	Female		13	43.3%
Reason of obstruction	UPJO*		4	13.3%
	Nephrolith		20	66.7%
	Tumor		6	20%
Degree of inflammatory infiltration	Mild		8	26.7%
	Moderate		9	30%
	Severe		13	43.3%
Degree of kidney fibrosis	Mild		6	20%
	Moderate		10	33.3%
	Severe		14	46.7%

*UPJO: Ureteropelvic Junction Obstruction

Normal controls (n=12)

			Number(n)	Percentage(%)
Age (years/Mean ± SD)	<18	2.8 ± 1.4	4	33.3%
	≥18	52.1 ± 16.3	8	66.7%
Gender	Male		5	41.7%
	Female		7	58.3%
