# Supplementary material for: Interferon regulatory factor 5 in myeloid cells promotes obstructive nephropathy-induced renal fibrosis in vivo
Source: JCI Insight. 2026 Aug 6;11(19):e201877. doi: 10.1172/jci.insight.201877 (PMC13726410; doi:10.1172/jci.insight.201877)
Supplement: Supplementary file 4 [file jciinsight-11-201877-s312.pdf]

Supplemental Table S10. The list of primers for RT-qPCR

Gene	Direction	Sequence
Actin	Forward	5'-CAG CCT TCC TTC TTG GGT ATG-3'
	Reverse	5'-GGC ATA GAG GTC TTT ACG GAT G-3'
Acta2	Forward	5'-GTC CCA GAC ATC AGG GAG TAA-3'
	Reverse	5'-TCG GAT ACT TCA GCG TCA GGA-3'
Il-1β	Forward	5'-CTG CAG CTG GAG AGT GTG G-3'
	Reverse	5'-GGG GAA CTC TGC AGA CTC AA-3'
Il-6	Forward	5'-GCC TTC TTG GGA CTG ATG CT-3'
	Reverse	5'-GCC ATT GCA CAA CTC TTT TCT CA-3'
Irf5	Forward	5'-AGA GAC AGG GAA GTA CAC TGA AG-3'
	Reverse	5'-TGG AAG TCA CGG CTT TTG TTA AG-3'
Tgfβ1	Forward	5'-CCA CCT GCA AGA CCA TCG AC-3'
	Reverse	5'-CTG GCG AGC CTT AGT TTG GAC-3'
Tnfα	Forward	5'-CAG GCG GTG CCT ATG TCT C-3'
	Reverse	5'-CGA TCA CCC CGA AGT TCA GTA G-3'
Vimentin	Forward	5'-CAC TAG CCG CAG CCT CTA TTC-3'
	Reverse	5'-GTC CAC CGA GTC TTG AAG CA-3'
