# Supplementary material for: Interferon regulatory factor 5 in myeloid cells promotes obstructive nephropathy-induced renal fibrosis in vivo
Source: JCI Insight. 2026 Aug 6;11(19):e201877. doi: 10.1172/jci.insight.201877 (PMC13726410; doi:10.1172/jci.insight.201877)
Supplement: Supplementary file 5 [file jciinsight-11-201877-s320.pdf]

Supplemental Table S9. The list of antibodies used for immunohistochemistry (IHC), immunofluorescence (IF) and western blot (WB)

Antigen	Company		Host	Reactivity	Dilution		
IRF5	abcam	ab181553	Rabbit	Mouse, Human	IHC 1:500	IF 1:1000	WB 1:1000
IRF5	abcam	ab21689	Rabbit	Mouse, Human	IF 1:2000	WB 1:5000	
IRF5	proteintech	3A4A10	Mouse	Mouse, Huamn	IF 1:500		
α-SMA	eBioscience	ab2572996	Mouse	Mouse, Human	IHC 1:500	IF 1:250	WB 1:100
Vimentin	abcam	ab92547	Rabbit	Mouse, Human	IHC 1:500	IF 1:500	WB 1:1000
CD68	abcam	ab283654	Rabbit	Mouse	IHC 1:100	IF 1:50	
CD20	abcam	ab64088	Rabbit	Mouse, Human	IHC 1:100	IF 1:100	
CD3	abcam	ab16669	Rabbit	Mouse, Human	IHC 1:500	IF 1:200	
MMP9	proteintech	10375-2-AP	Rabbit	Mouse, Human	IHC 1:400	IF 1:200	WB 1:1000
MMP9	Invitrogen	MA5-15886	Mouse	Mouse, Human	IF 1:1000		
GAPDH	Invitrogen	ab10977387	Mouse	Mouse	WB 1:5000		
β-actin	Invitrogen	ab2745831	Rabbit	Mouse	WB 1:10000		
F4/80	proteintech	28463-1-AP	Rabbit	Mouse	WB 1:3000		
TNF-α	proteintech	60291-1-Ig	Mouse	Mouse	WB 1:1000		
